# Tricyclic coumarin sulphonate derivatives with alkaline phosphatase inhibitory effects: *in vitro* and docking studies

**DOI:** 10.1080/14756366.2018.1428193

**Published:** 2018-02-02

**Authors:** Jamshed Iqbal, Mohammed I. El-Gamal, Syeda Abida Ejaz, Joanna Lecka, Jean Sévigny, Chang-Hyun Oh

**Affiliations:** a Centre for Advanced Drug Research, COMSATS Institute of Information Technology, Abbottabad, Pakistan;; b Department of Medicinal Chemistry, College of Pharmacy, University of Sharjah, Sharjah, United Arab Emirates;; c Sharjah Institute for Medical Research, University of Sharjah, Sharjah, United Arab Emirates;; d Department of Medicinal Chemistry, University of Mansoura, Mansoura, Egypt;; e Département de microbiologie-infectiologie et d'immunologie, Faculté de Médecine, Université Laval, Québec, Canada;; f Centre de Recherche du CHU de Québec, Université Laval, Québec, Canada;; g Center for Biomaterials, Korea Institute of Science and Technology, Seoul, Republic of Korea;; h Department of Biomolecular Science, University of Science and Technology, Daejeon, Republic of Korea

**Keywords:** Alkaline phosphatase inhibitor, coumarin, molecular docking, structure-activity relationship, Tricyclic coumarin sulfonate

## Abstract

Tissue-nonspecific alkaline phosphatase (TNAP) is an important isozyme of alkaline phosphatases, which plays different pivotal roles within the human body. Most importantly, it is responsible for maintaining the balanced ratio of phosphate and inorganic pyrophosphate, thus regulates the extracellular matrix calcification during bone formation and growth. The elevated level of TNAP has been linked to vascular calcification and end-stage renal diseases. Consequently, there is a need to search for highly potent and selective inhibitors of alkaline phosphatases (APs) for treatment of disorders associated with the over-expression of APs. Herein, a series of tricyclic coumarin sulphonate **1a-za** with known antiproliferative activity, was evaluated for AP inhibition against human tissue nonspecific alkaline phosphatase (*h*-TNAP) and human intestinal alkaline phosphatase (*h-*IAP). The methylbenzenesulphonate derivative **1f** (IC_50_ = 0.38 ± 0.01 μM) was found to be the most active *h*-TNAP inhibitor. Another 4-fluorobenzenesulphonate derivative **1i** (IC_50_ = 0.45 ± 0.02 μM) was found as the strongest inhibitor of *h*-IAP. Some of the derivatives were also identified as highly selective inhibitors of APs. Detailed structure-activity relationship (SAR) was investigated to identify the functional groups responsible for the effective inhibition of AP isozymes. The study was also supported by the docking studies to rationalise the most possible binding site interactions of the identified inhibitors with the targeted enzymes.

## Introduction

**Figure 1.**
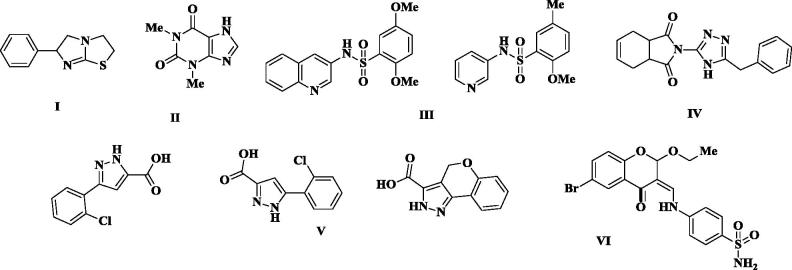
APs inhibitors; (I) Levamisole (II) Theophylline (III) Aryl sulphonamide-based inhibitors (IV) trizole-based inhibitors (V) Pyrazole-based Inhibitors, (VI) coumarinyl sulphonates.

Alkaline phosphatases (APs, E.C. 3.1.3.1) are membrane-bound ecto-enzymes with an extracellular-oriented active site^1^. They belong to the large family of ecto-enzyme known as ecto-nucleotidases, and are involved in various physiological functions within the human body, most importantly in phosphorylation and dephosphorylation reactions^2^. They are also responsible for the hydrolysis and breakdown of wide variety of nucleoside tri- and di-phosphates substrates to their respective monophosphates^3^^,^^4^. They also hydrolyse many other phosphate-containing substrates, such as inorganic pyrophosphate (PPi), glucose phosphate, polyphosphates, phosphomonoesters, and phosphatidates^5^. APs are further categorised into two types, the tissue-specific and tissue-nonspecific alkaline phosphatases (TNAPs). From the tissue-specific AP type, intestinal alkaline phosphatase (IAP) plays a pivotal role in the maintenance of physiological environment of the intestine^6^. Moreover, this isozyme is important for the regulation of bicarbonate secretion balance, duodenal luminal pH, dephosphorylation reaction, and helps in the maintenance of normal gut environment by detoxifying the bacterial toxins^7^. TNAP is widely distributed almost in each body part but is abundantly present in central nervous tissues, mineralising tissues and also in kidney^8^. As compared to the other body tissues, high concentration of TNAP is present in the mineralising tissues such as teeth and bones for the normal teeth and bone formation. But too high concentrations of TNAP in mineralising tissue results in abnormal calcification and mineral deposition^9^. The identification of effective inhibitors of AP isozymes becomes an emerging drug target for the disorders related to the hyper-activity of TNAP and IAP. To date, different inhibitors of APs based on triazole, pyrazoles, coumarin sulphonates, diaryl sulphonamides, and chromones have been identified as effective inhibitors of TNAP and IAP (Figure 1)^10–13^.

**Figure 2.**
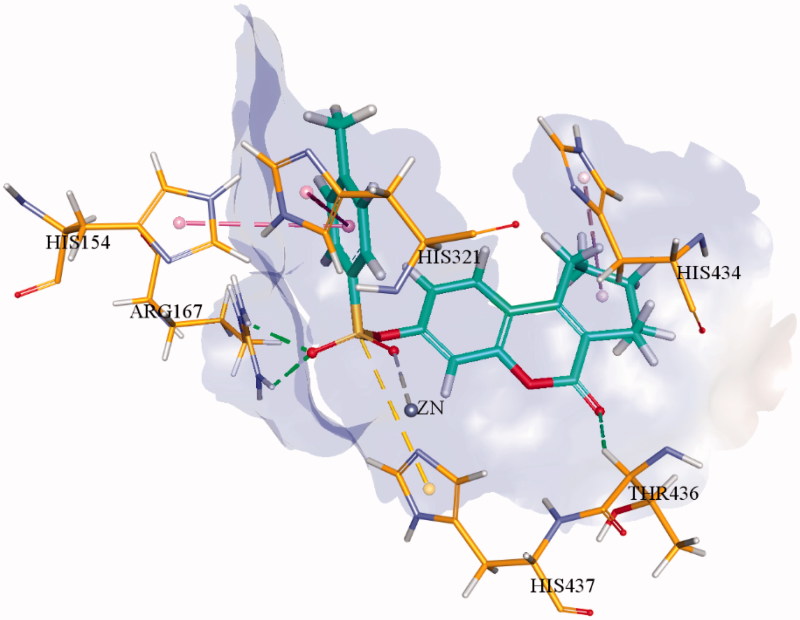
3D binding interactions of most potent inhibitor **1f** within the active site of *h*-TNAP. Hydrogen bonds are shown in green dotted line while π–π interactions are shown in purple dotted line.

Recently, we have reported coumarinyl sulphonates derivatives as novel and potent inhibitors of *h*-TNAP and *h*-IAP^13^. In continuation of our efforts for searching the effective and selective inhibitors, herein we evaluated a series of 27 tricyclic-fused coumarin sulphonate derivatives **1a-za** as inhibitors of APs which were already tested for their antiproliferative and anti-inflammatory activities^14–17^. We assumed that the fused cycloheptane or cyclooctane ring might contribute to stronger affinity than the previously reported bicyclic coumarin sulphonate derivatives through formation of additional hydrophobic interaction(s). Our assumption succeeded as shown in Figure 2. Except two derivatives **1w** and **1 y**, all the other compounds were found as effective AP inhibitors with sub-micromolar or very low micro-molar range IC_50_ values. Further, the most effective inhibitors of either *h*-TNAP or *h*-IAP were selected for the molecular docking studies to rationalise the possible binding interactions with the respective targeted enzymes.

## Experimental

### Synthesis of the target molecules 1a-za

The synthetic procedures, purification methods, and the spectral analysis data have been reported^14–17^. The experimental details as well as representative analysis charts are provided in the supplementary file.

## Bioactivity protocol

### Alkaline phosphatase inhibition assay (*h*-TNAP and *h*-IAP)

The inhibition assay was performed by following the already reported method^18^ and the inhibitory activity was measured at the final concentration of 200 µM in diethanolamine (DEA) buffer as discussed in our published article^19^. The first step of the inhibition assay was the treatment of 10 µl of tested derivatives with 20 µl of *h*-TNAP (46 ng/well) or of *h*-IAP (57 ng/well). The plates were incubated at 37 °C for 5–10 min, and luminescence signals were recorded by microplate reader (BioTek FLx800, Instruments, Inc., Winooski, VT). Then, 20 µl of CDP-star^®^ substrate was added to each reaction mixture and signals were again measured as after-read, after 15–20 min of incubation. The data were scrutinised by PRISM 5.0 (GraphPad, San Diego, CA) software and the inhibitory concentration values (IC_50_ values) were attained as discussed earlier.

### Molecular docking studies

Molecular docking studies of compounds **1f** and **1i**, potent inhibitors of *h*-TNAP and *h*-IAP, respectively, were conducted to investigate the probable binding interactions of these compounds within the active site of the respective target enzyme. Our previously reported models of *h*-TNAP and *h*-IAP were used for docking calculation^20^. Before performing docking studies, protonation and energy minimisation of all enzyme structures were carried out using molecular operating environment (MOE) 2014, 09 software^21^. Structure generation as well as energy minimisation of the effective inhibitors were also carried out via MOE software. After preparation of all the prerequisite compounds and enzymes structures, total 50 independent docking runs for each ligand were executed by MOE docking program. The best scored pose of potent compounds in each target enzyme was selected for evaluation of binding interactions. Binding free energy for each pose was also determined and pose with the lowest free binding energy was selected for further visualisation studies. 3D visualisation of binding interaction between potent compound and respective target was carried out using Discovery studio visualizer v4^22^.

## Results and discussion

### Chemistry

**Scheme 1.**
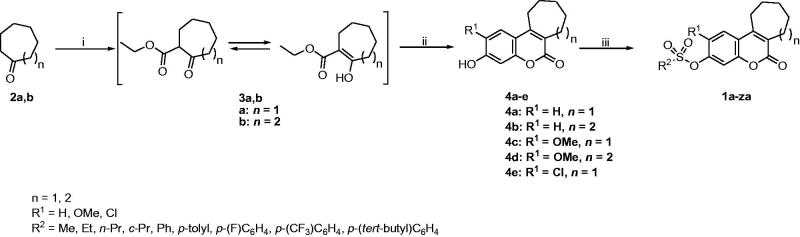
Reagents and conditions: (i) diethyl carbonate, NaH, benzene, reflux, 90% (**3a**, *n* = 1), 85% (**3 b**, *n* = 2); (ii) (substituted) resorcinol, CF_3_COOH, conc. H_2_SO_4_, 0 °C; rt, 3 h; (iii) appropriate sulphonyl chloride derivative, triethylamine, CH_2_Cl_2_, 0 °C; rt, 1 h.

Scheme 1 illustrates the synthetic pathway via which the target compounds **1a-za** were prepared. Cycloheptanone (**2a**) or cyclooctanone (**2b**) was reacted with diethyl carbonate in the presence of NaH in benzene at the reflux temperature to get the ester intermediates **3a,b** that exist in keto-enol tautomers^23^. Subsequent reaction with (substituted) resorcinol in the presence of trifluoroacetic acid and concentrated sulphuric acid led to the production of the tricyclic phenolic intermediates **4a-f**^24^. They were used in the next step as such with no prior purification. In the last step, those phenolic intermediates were treated with the appropriate sulphonyl chloride reagent in the presence of triethylamine as a catalyst to get the target sulphonate products **1a-za**^14–17^. Table 1 illustrates the structure of each target compound.

**Table 1.** Structures of the target compounds **1a-za** along with their yield percentages and *in vitro* inhibitory effects (IC_50_, μM) against *h*-TNAP and *h*-IAP.


					IC_50_ (µM) ± SEM^a^	
Compound No.	R^1^	R^2^	*n*	Yield%	*h*-TNAP	*h*-IAP
**1a**	H	Me	1	95	0.48 ± 0.01	1.98 ± 0.19
**1b**	H	Et	1	92	2.13 ± 0.19	2.71 ± 0.23
**1c**	H	*n*-Pr	1	93	1.52 ± 0.17	0.91 ± 0.06
**1d**	H	Cyclo-Pr	1	85	0.98 ± 0.13	0.96 ± 0.08
**1e**	H	Ph	1	93	8.57 ± 0.73	1.84 ± 0.19
**1f**	H	*p*-Tolyl	1	95	0.38 ± 0.01	1.94 ± 0.11
**1g**	H	*p*-(CF_3_)C_6_H_4_	1	90	1.59 ± 0.12	2.64 ± 0.45
**1h**	H	*p*-(*tert*-butyl)C_6_H_4_	1	95	6.33 ± 0.99	3.92 ± 0.47
**1i**	H	*p*-(F)C_6_H_4_	1	82	1.06 ± 0.11	0.45 ± 0.09
**1j**	H	Et	2	90	1.74 ± 0.14	0.84 ± 0.09
**1k**	H	*n*-Pr	2	88	1.91 ± 0.11	3.37 ± 0.79
**1l**	H	Ph	2	92	1.13 ± 0.21	2.82 ± 0.82
**1m**	H	*p*-Tolyl	2	95	1.59 ± 0.16	2.12 ± 0.23
**1n**	H	*p*-(*tert*-butyl)C_6_H_4_	2	94	1.85 ± 0.13	1.28 ± 0.13
**1o**	OMe	Me	1	85	1.21 ± 0.11	11.80 ± 1.91
**1p**	OMe	Me	2	85	1.49 ± 0.13	1.51 ± 0.17
**1q**	OMe	*n*-Pr	1	95	4.31 ± 0.99	>100
**1r**	OMe	*n*-Pr	2	91	2.60 ± 0.49	0.75 ± 0.07
**1s**	OMe	Ph	1	92	1.26 ± 0.07	3.88 ± 0.32
**1t**	OMe	Ph	2	87	2.68 ± 0.18	0.83 ± 0.06
**1u**	OMe	*p*-Tolyl	1	96	1.65 ± 0.13	0.79 ± 0.05
**1v**	OMe	*p*-Tolyl	2	90	0.64 ± 0.02	1.45 ± 0.04
**1w**	OMe	*p*-(CF_3_)C_6_H_4_	1	87	>100	>100
**1x**	OMe	*p*-(CF_3_)C_6_H_4_	2	85	1.29 ± 0.12	1.63 ± 0.19
**1y**	Cl	Me	1	85	>100	>100
**1z**	Cl	Ph	1	85	5.73 ± 1.06	>100
**1za**	Cl	*p*-Tolyl	1	86	2.22 ± 0.27	1.11 ± 0.01
**Levamisole**	——	——	——	——	20.2 ± 1.90	——
**L-Phenylalanine**	——	——	——	——	——	100 ± 3.15

a IC_50_ is the concentration at which the 50% of the enzyme activity was inhibited. All the values were expressed as IC_50_ ± SEM (standard error mean), *n* = 3.

### Alkaline phosphatase inhibition

All synthesised tricyclic coumarin sulphonate derivatives **1a-za** were tested for their potential to inhibit *h*-TNAP and *h*-IAP. Except two compounds (**1w** and **1 y**), all compounds were found to be active against both isozymes of AP with IC_50_ values in the lower micro-molar range. Compounds showed their *h*-TNAP inhibitory potential in the range of IC_50_ = 0.38 ± 0.01 to 8.57 ± 0.73 μM as compared to standard levamisole (IC_50_ = 20.21 ± 1.9 μM), and *h*-IAP inhibitory activity in the range of IC_50_ = 0.45 ± 0.09 to 11.8 ± 1.91 μM as compared to standard L-phenylalanine (IC_50_ = 100.1 ± 3.15 μM) (Table 1).

### Structure-activity relationship (SAR)

In order to find out the comprehensive structure-activity relationship for AP inhibition, all tricyclic-fused coumarin sulphonates **1a-za** were divided into two categories, on the basis of fused cycloheptane and cyclooctane ring. Moreover, the detailed structure-selectivity relationship was also rationalised on the basis of effect of unsubstituted, and chloro- and methoxy-substituted derivatives on the inhibition of both APs. Compounds **1q** and **1z** were found as selective inhibitors of *h*-TNAP while none of the derivative was found as a selective inhibitor of IAP.

Among the tested derivatives, compound **1f** expressed maximum inhibitory effects on *h*-TNAP and compound **1i** depicted the strongest potency against *h*-IAP. From the unsubstituted derivatives, except compound **1c**, **1g**, and **1m**, the other target derivatives expressed varying inhibitory effects on *h-*TNAP. Compounds **1f, 1a**, and **1d** possessing *p*-toluene sulphonate, methane sulphonate, and cyclopropane substitution, respectively, depicted higher inhibition of *h*-TNAP than compounds **1c**, **1g**, and **1j-m**. Among the cyclooctane-fused derivatives, compound **1l** with benzenesulphonate substitution demonstrated the strongest inhibitory potential on *h*-TNAP (IC_50_ ± SEM = 1.13 ± 0.21 µM). Compounds **1c** with the fused cycloheptane ring and compound **1j** with the fused cyclooctane ring were found as stronger inhibitors of *h*-IAP with the inhibitory values of IC_50_ ± SEM = 0.91 ± 0.06 and 0.84 ± 0.09 µM, respectively. Among the cyclooctane-fused derivatives, compound **1j** with ethanesulphonate substitution demonstrated the strongest inhibitory effect on *h*-IAP (IC_50_ ± SEM = 0.84 ± 0.09 µM). From the cycloheptane-fused derivatives, compound **1c** with propanesulphonate substitution demonstrated the strongest inhibitory potential on *h*-IAP (IC_50_ ± SEM = 0.91 ± 0.06 µM). Although more lipophilic side chain was substituted in **1c**, the inhibitory effect of this derivative was found less because of the presence of seven-membered fused ring. Moreover, from the obtained results this may be rationalised that the presence of more lipophilic side chain in compound **1c** may form stronger hydrophobic interactions with the target enzyme.

The inhibitory effect by other derivatives; **1h** and **1n** with *tert*-butylbenzene suphonate moiety expressed the dual inhibition of both isozymes of APs. The derivative **1n** depicted more inhibitory potential because of the presence of fused eight membered ring (*h*-TNAP; IC_50_±SEM = 1.85 ± 0.13 µM, *h*-IAP; IC_50_±SEM = 1.28 ± 0.13 µM). In comparison to **1h**, the replacement of *tert*-butylbenzene with flouorobenzenesulphonate results in the most potent inhibitor of *h*-IAP i.e. **1i**. Compound **1i** was identified as the strongest inhibitor of *h*-IAP with and inhibitory value of IC_50_±SEM = 0.45 ± 0.09 µM. The increased potency might be due to the presence of most electronegative halogen.

On the other hand, the chloro- and methoxy-substituted derivatives were found more active against both APs than the unsubstituted analogues. With reference to *h*-TNAP, compound **1o** (IC_50_±SEM = 1.21 ± 0.11 µM) was found more potent as compared to compound **1q** (IC_50_±SEM = 4.31 ± 0.99 µM), **1p** (IC_50_±SEM = 1.49 ± 0.13 µM), and **1r** (IC_50_±SEM = 2.60 ± 0.49 µM) but with reference to *h*-IAP, **1p** (IC_50_±SEM = 1.51 ± 0.17 µM) was found more better than **1o** (IC_50_±SEM = 11.8 ± 1.91 µM) and **1r** (IC_50_±SEM = 0.75 ± 0.07 µM) was found better than **1q** (IC_50_ ± SEM = >100 µM). It means that the presence of functional group is playing more important role on the inhibitory effects. The derivatives with less carbon number were found more potent that the propane derivative. But, with detailed SAR it was found that the heptane ring was favourable for TNAP, while octanone derivative was found potent towards IAP. When the activity of **1o** was compared with **1r** and **1p**, it was observed that the reduced inhibitory effect of both the derivatives on TNAP was due to the presence of *n*-propane substitution in case of **1r**, and the presence of fused cyclooctane ring in case of **1p**.

In comparison to **1o**, compound **1s** having benzenesulphonate substitution was found almost as an equipotent inhibitor of TNAP with the inhibitory value of 1.26 ± 0.07 µM. The other derivative i.e. **1t** having the same substitution of benzenesulphonate as in compound **1s**, but with the cyclooctane ring, a reduced inhibitory effect on TNAP was observed with an increased inhibitory effect on IAP isozymes (*h*-TNAP; (IC_50_±SEM = 2.68 ± 0.18 µM, *h*-IAP; IC_50_±SEM = 0.83 ± 0.06 µM). The derivatives **1w** and **1x** having the same substitution at R^1^ and R^2^ positions but with cycloheptane and cyclooctane ring in **1w** and **1x,** respectively. When the SAR of these two derivatives was considered, it was observed that cycloheptane derivative **1w** was found less reactive than that of the cyclooctane derivative **1x** which inhibited both isozymes effectively. This inhibitory effect confirms that like **1p**, the presence of more carbon number in the derivative is responsible for the more inhibition.

An interesting inhibition of IAP was also exhibited by the aromatic derivative **1u** and **1za** processing cycloheptane ring along with *p*-tosylate substitution at R^2^ position but different substitution at R^1^ position. The derivative with electron-donating group i.e. OMe in case of **1u** (IC_50_±SEM = 0.79 ± 0.05 µM) was found more active inhibitor as compared to the derivative **1za** (IC_50_±SEM = 1.11 ± 0.01 µM) with electron-withdrawing substitution. These two derivative also exert some inhibitory potential on *h*-TNAP, but less (**1u**; IC_50_±SEM = 1.65 ± 0.13 µM, **1za**; IC_50_±SEM = 2.22 ± 0.27 µM) in comparison to *h*-IAP. The other derivatives **1 y** and **1z** with methanesulphonate and benzenesulphonate substitution were found inactive towards *h*-IAP. Moreover, it was found that the aromatic sulphonate derivatives **1s**, **1u**, and **1za** depicted stronger inhibitory effects on *h*-IAP than *h*-TNAP.

The results obtained here are in consistent with our previously reported work. The 2-methoxy- or 2-chloro- substituted derivatives possessing the fused cycloheptane ring were found stronger inhibitors of *h*-TNAP than the corresponding analogues having fused cyclooctane ring. Similarly, an inverse effect was observed towards *h*-IAP. It can be suggested from the obtained data that the derivatives with more lipophilic side chains led to the more inhibition of *h*-IAP and vice versa in case of *h*-TNAP. This lipophilic side chains might be involved in some hydrophobic interactions within the pocket of the receptor site and increase the affinity of such derivatives for the targeted enzyme and ultimately accounts for the enhanced *h*-IAP inhibition.

### Molecular docking studies

**Figure 3.**
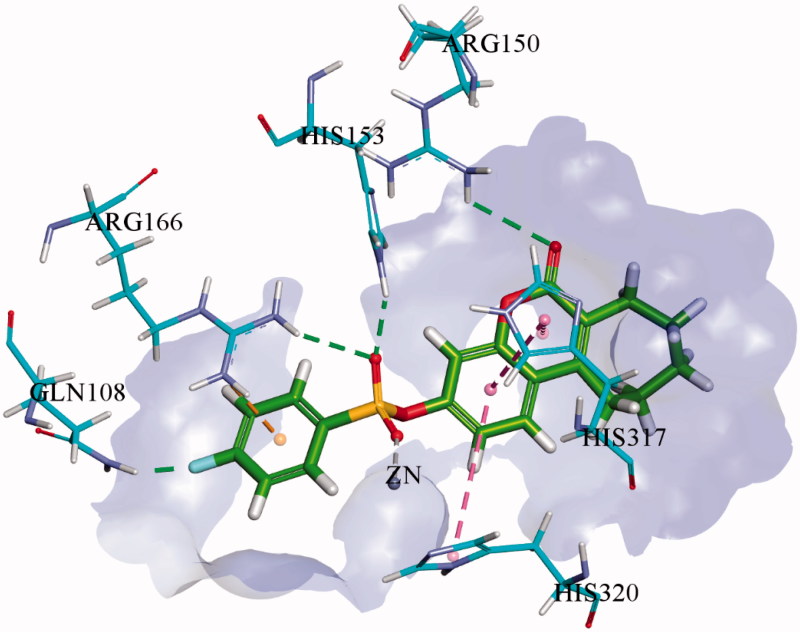
3D binding interactions of most potent inhibitor **1i** within the active site of *h*-IAP. Hydrogen bonds are shown in green dotted line while π–π interactions are shown in purple dotted line.

Molecular docking studies of the most potent derivatives i.e. **1f** and **1i** were carried out within the respective target enzyme and their most probable binding interactions within the modelled enzymes are depicted in Figures 2 and 3. Detailed analysis of binding interaction of compound **1f** within *h*-TNAP revealed that oxygen atom in both sulphonate group and carbonyl group are responsible for making three strong hydrogen bonds with amino acid residues. Two hydrogen bonds were formed by oxygen of sulphonate group with amino groups of Arg167 while the third hydrogen bond was formed by carbonyl oxygen in coumarin ring with amino acid Thr436. Additional to hydrogen bonding, second oxygen atom of the sulphonate group also formed direct interaction with the Zn^+2^ ion within the active site of *h*-TNAP. The excellent *h*-TNAP inhibition activity of compound **1f** can be related with this direct binding interaction of sulphonate oxygen with the Zn^+2^ ion of the active site. Furthermore, two π–π interactions were formed by benzene ring adjacent to sulphonate group with amino acid residues His154 and His321. One π-alkyl interaction was also exhibited by the seven-membered ring fused with the coumarin ring and His434 as shown in Figure 2. This interaction may account for the stronger affinity and potency of this tricyclic compound compared to the previously reported bicyclic coumarin sulphonate derivatives^13^. Detailed binding interaction of **1i** with *h*-IAP is shown in Figure 3. In comparison to docking interaction of compound **1f** with *h*-TNAP, compound **1i** formed four hydrogen bonds with different amino acids of *h*-IAP. Oxygen atom in sulphonate group of compound **1i** also formed two hydrogen and one metal interaction. These two hydrogen bonds were formed by one oxygen atom in sulphonate group with amino acid residue of Arg166 and His143. While other oxygen of sulphonate group formed interaction with Zn^+2^ ion. Carbonyl oxygen in coumarin ring and fluorine atom in compound **1i** also formed hydrogen bond with amino acid residues Arg150 and Gln108 as shown in Figure 3. Additional to hydrogen bonding, two π–π interaction were also formed by benzene ring of coumarin ring with amino acid residue of His317 and His320. Metal interaction of sulphonate group with metal ion is analogous to previously reported interaction of sulphonamides with different APs^13^. π-alkyl interaction between seven-membered ring adjacent to coumarin ring was not shown as in **1i**. Docking of the inactive derivatives **1q** and **1w** showed weaker affinity and fewer bonds formed, which supports the SAR discussion. The figures are given in the supplementary file.

## Conclusions

Tricyclic-fused coumarin sulphonates **1a-za** were evaluated for their effective inhibitory potential against *h*-TNAP and *h*-IAP. Except **1w** and **1 y** derivatives, all the other compounds exhibited excellent inhibitory potential against both *h*-TNAP and *h*-IAP. Methylbenzenesulphonate derivative **1f** (IC_50_ = 0.38 ± 0.01 μM) was found to be the most active *h*-TNAP inhibitor. Another 4-fluorobenzenesulphonate derivative **1i** (IC_50_ = 0.45 ± 0.02 μM) was found as the strongest inhibitor of *h*-IAP. Detailed SAR studies and molecular docking studies were performed to identify the importance of functional groups within the structures and to explore the possible binding site interactions, respectively. Moreover, previously these derivatives have been reported for their antiproliferative effects against different cell lines including, leukaemia, CNS, prostate, renal, colon, lung cancer, melanoma, ovarian, and breast cancers. From that data we got **1i** and **1 m** as the strongest antiproliferative derivatives against colon cancer cell line along with the maximum anti-inflammatory effects. Here in this study, we have identified these compounds as stronger inhibitors of *h*-IAP isozymes. This can be rationalised that the IAP, responsible for the maintenance of intestinal homeostasis, might be over-expressed in the colon cancer cell lines. Moreover, the over-expression of *h*-TNAP can be associated with the lung cancer, melanoma, ovarian, breast cancers, and renal cancer. Therefore, the obtained results synergise with the previously reported data. It can be suggested that the potent inhibitors of both *h*-TNAP and *h*-IAP can be selected for further exploration at molecular level.

## Supplementary Material

IENZ_1428193_Supplementary_Material.pdf
