# Supplementary material for: Tricyclic coumarin sulphonate derivatives with alkaline phosphatase inhibitory effects: in vitro and docking studies
Source: J Enzyme Inhib Med Chem. 2018 Feb 2;33(1):479–84. doi: 10.1080/14756366.2018.1428193 (PMC6009858; doi:10.1080/14756366.2018.1428193)
Supplement: IENZ_1428193_Supplementary_Material.pdf [file IENZ_A_1428193_SM8934.pdf]

Tricyclic coumarin sulfonate derivatives with alkaline phosphatase inhibitory effects: *In vitro* and docking studies

Jamshed Iqbal [1,$], Mohammed I. El-Gamal [2,3,4,$], Syeda Abida Ejaz [1], Joanna Lecka [5,6], Jean Sévigny [5,6], and Chang-Hyun Oh [7,8,*]

[1] Centre for Advanced Drug Research, COMSATS Institute of Information Technology, Abbottabad 22060, Pakistan

[2] Department of Medicinl Chemistry, College of Pharmacy, University of Sharjah, Sharjah 27272, United Arab Emirates.

[3] Sharjah Institute for Medical Research, University of Sharjah, Sharjah 27272, United Arab Emirates.

[4] Department of Medicinal Chemistry, Faculty of Pharmacy, University of Mansoura, Mansoura 35516, Egypt.

[5] Département de microbiologie-infectiologie et d'immunologie, Faculté de Médecine, Université Laval, Québec, QC, G1V 0A6, Canada

[6] Centre de Recherche du CHU de Québec – Université Laval, Québec, QC, G1V 4G2, Canada

[7] Center for Biomaterials, Korea Institute of Science and Technology, PO Box 131, Cheongryang, Seoul 130-650, Republic of Korea.

[8] Department of Biomolecular Science, University of Science and Technology, 113 Gwahangno, Yuseong-gu, Daejeon 305-333, Republic of Korea.

[*] Corresponding author: Tel.: +82 2 958 5160; fax: +82 2 958 5308; E-mail address: choh@kist.re.kr; Address: Center for Biomaterials, Korea Institute of Science and Technology, PO Box 131, Cheongryang, Seoul 130-650, Republic of Korea [C.-H. Oh].

[$] Both Jamshed Iqbal and Mohammed I. El-Gamal are first authors.

Supplementary data	Page No.
Synthetic experimental procedures...………………………………………………	2-14
Representative ^{1}H NMR, ^{13}C NMR, and MS spectra...……………..…………….	15-100
Docking poses of the inactive derivatives **1q** and **1w**..	101

Synthetic experimental procedures

General

The target compounds were purified by column chromatography using silica gel (0.040-0.063 mm, 230-400 mesh) and technical grade solvents. The melting points were obtained on a Walden Precision Apparatus Electrothermal 9300 apparatus and are uncorrected. Nuclear magnetic resonance (NMR) spectroscopy was performed using a Bruker ARX-300, 300 MHz (Bruker Bioscience, Billerica, MA, USA) and a Bruker ARX-400, 400 MHz (Bruker Bioscience, Billerica, MA, USA) with TMS as an internal standard. Purities of the target compounds (>96%) were determined by elemental analysis. LC-MS analysis was conducted using the following system: Waters 2998 photodiode array detector, Waters 3100 mass detector, Waters SFO system fluidics organizer, Waters 2545 binary gradient module, Waters reagent manager, Waters 2767 sample manager, Sunfire^{TM} C18 column (4.6 x 50 mm, 5 μm particle size); Solvent gradient = 95% A at 0 min, 1% A at 5 min; solvent A: 0.035% trifluoroacetic acid (TFA) in water; solvent B: 0.035% TFA in CH_3OH; flow rate = 3.0 mL/min; the area under curve (AUC) was calculated using Waters MassLynx 4.1 software. Unless otherwise noted, all solvents and reagents were commercially available and used without further purification.

Synthesis of ethyl 2-oxocycloheptanecarboxylate (3a) and ethyl 2-oxocyclooctanecarboxylate (3b)

A 250 mL two-neck, round-bottomed flask equipped with a magnetic stirrer was fitted with a 50 mL pressure-equalizing constant-rate dropping funnel and a condenser. To the flask, sodium hydride (4.5 g, 112 mmol, 60% dispersion in mineral oil) was added. The mineral oil was removed by washing the dispersion four times with 20 mL portions of dry benzene under nitrogen atmosphere. The benzene was removed with a pipette after the sodium hydride was allowed to settle. After removal of most of the mineral oil, 60 mL of dry benzene was added to the sodium hydride, followed by diethyl carbonate (6.5 g, 55 mmol), this mixture was heated to reflux, and a solution of cycloheptanone (**2a**) or cyclooctanone (**2b**) (30 mmol) in 10 mL of dry benzene was added dropwise over a period of 3-4 h. After the addition was completed, this

mixture was allowed to reflux until the evolution of hydrogen gas ceases (15-20 min). The reaction mixture was allowed to cool to room temperature, and 10 mL of glacial acetic acid was added dropwise, a heavy pasty solid separated. Then ice-cold water (about 100 mL) was added dropwise, and the stirring was continued until all the solid material has dissolved. The benzene layer was separated, and the aqueous layer was extracted with benzene (3 x 50 mL). The combined benzene extracts were washed three times with cold water (3 x 50 mL). The organic layer was dried over anhydrous sodium sulfate, and the solvent was evaporated under reduced pressure. The title compounds were purified by flash column chromatography. The products were existing in equilibrating mixtures of the keto and enol tautomers.

*Compound **3a**:* it was purified by flash column chromatography (silica gel, hexanes:ethyl acetate 15:1 v/v); yield: 90%; yellow oil; ^{1}H NMR ($CDCl_3$, 400 MHz) δ 12.76 (brs, 1H), 4.21-4.15 (m, 2H), 3.55-3.52 (m, 2H), 2.63-2.58 (m, 2H), 2.48-2.36 (m, 1H), 2.08 (t, 1H, J = 2.5 Hz), 1.94-1.85 (m, 4H), 1.62-1.61 (m, 2H), 1.49-1.44 (m, 2H), 1.32-1.23 (m, 4H); ^{13}C NMR ($CDCl_3$, 100 MHz) δ 208.8, 179.4, 173.0, 170.4, 101.5, 77.5, 77.2, 76.9, 60.9, 58.8, 43.0, 35.3, 31.9, 29.6, 27.9, 27.5, 27.3, 24.6, 24.3, 14.0; LC-MS: 185.1 (M^+ + 1).

*Compound **3b**:* it was purified by flash column chromatography (silica gel, hexanes:ethyl acetate 10:1 v/v); yield: 85%; yellow oil; ^{1}H NMR ($CDCl_3$, 400 MHz) δ 12.60 (brs, 1H), 4.23-4.12 (m, 3H), 2.41-2.33 (m, 4H), 2.15-2.08 (m, 1H), 1.93-1.86 (m, 1H), 1.75-1.65 (m, 3H), 1.57-1.45 (m, 8H), 1.30 (t, 3H, J = 7.1 Hz), 1.24 (t, 3H, J = 7.1 Hz); ^{13}C NMR ($CDCl_3$, 100 MHz) δ 212.2, 176.0, 172.9, 170.1, 99.2, 61.1, 60.1, 57.0, 41.7, 32.3, 29.9, 28.9, 28.7, 27.0, 26.5, 26.0, 25.5, 25.2, 24.5, 23.8, 14.3, 14.0; LC-MS: 199.1 (M^+ + 1).

Synthesis of the hydroxyl intermediates 4a-e

Resorcinol or 4-methoxyresorcinol or 4-chlororesorcinol (11.0 mmol) was dissolved in hot ethyl 2-oxocycloheptanecarboxylate (**3a**) or ethyl 2-oxocyclooctanecarboxylate (**3b**) (11.0

mmol). To this stirred mixture at ice-water temperature was added dropwise a mixture of trifluoroacetic acid (1.7 mL, 22.0 mmol) and conc. sulfuric acid (2.2 mL, 22.0 mmol) at such a rate that the reaction temperature was kept below 10ºC (about 30 min). The reaction mixture was then allowed to warm to room temperature and then stirred for an additional 3 h before being quenched cautiously with ice-water. The product was then extracted with ethyl acetate (3 x 20 mL), and the combined organic layer extracts were washed with brine (3 x 25 mL) and dried over anhydrous sodium sulfate. The organic solvent was evaporated under reduced pressure, and the product was used in the next step without further purification.

Synthesis of the target cycloalkane-fused coumarin sulfonates 1a-za

A solution of the appropriate intermediate hydroxyl compound **4a-e** (0.1 mmol) and triethylamine (0.0165 mL, 0.2 mmol) in dry dichloromethane (5 mL) was cooled in an ice bath. A solution of the appropriate sulfonyl chloride derivative (0.11 mmol) in dry dichloromethane (2 mL) was added dropwise at the same temperature. The reaction mixture was allowed to warm to room temperature, and the stirred for an additional 1 h. The reaction mixture was washed with brine (3 x 5 mL) and dried over anhydrous sodium sulfate. The organic solvent was evaporated under reduced pressure, and the product was purified by flash column chromatography.

*6-Oxo-6,7,8,9,10,11-hexahydrocyclohepta[c]chromen-3-yl methanesulfonate (**1a**)*

It was purified by flash column chromatography (silica gel, hexanes:ethyl acetate 5:1 v/v); mp: 172-3 ºC; ^{1}H NMR ($CDCl_3$, 400 MHz) δ 7.72 (d, 1H, *J* = 9.4 Hz), 7.27-7.24 (m, 2H), 3.22 (s, 3H), 2.97-2.91 (m, 4H), 1.92 (d, 2H, *J* = 5.4 Hz), 1.70-1.62 (m, 4H); ^{13}C NMR ($CDCl_3$, 100 MHz) δ 161.4, 153.1, 152.8, 150.0, 129.2, 125.5, 119.1, 118.0, 110.6, 37.9, 31.9, 28.2, 26.9, 25.5, 24.9; LC-MS: 309.1 (M^+ + 1); elemental analysis ($C_{15}H_{16}O_5S$): calculated C: 58.43%, H: 5.23%, S: 10.40%; found: C: 58.30%, H: 5.43%, S: 10.62%.

*6-Oxo-6,7,8,9,10,11-hexahydrocyclohepta[c]chromen-3-yl ethanesulfonate (**1b**)*

It was purified by flash column chromatography (silica gel, hexanes:ethyl acetate 4:1 v/v); mp: 169-72 ºC; ^{1}H NMR ($CDCl_3$, 400 MHz) δ 7.71 (t, 1H, *J* = 4.6 Hz), 7.24-7.23 (m, 2H), 3.35 (q, 2H, *J* = 7.4 Hz), 2.96-2.90 (m, 4H), 1.91 (d, 2H, *J* = 5.5 Hz), 1.70-1.62 (m, 4H), 1.58 (t, 3H, *J* = 7.4 Hz); ^{13}C NMR ($CDCl_3$, 100 MHz) δ 161.4, 153.1, 152.8, 150.0, 129.0, 125.4, 118.9, 117.9, 110.7, 45.6, 1.9, 28.2, 26.9, 25.5, 24.9, 8.2; LC-MS: 323.1 (M^+ + 1); elemental analysis ($C_{16}H_{18}O_5S$): calculated C: 59.61%, H: 5.63%, S: 9.95%; found: C: 59.52%, H: 5.48%, S: 10.06%.

*6-Oxo-6,7,8,9,10,11-hexahydrocyclohepta[c]chromen-3-yl propane-1-sulfonate (**1c**)*

It was purified by flash column chromatography (silica gel, hexanes:ethyl acetate 5:1 v/v); mp: 133-5 ºC; ^{1}H NMR ($CDCl_3$, 300 MHz) δ 7.72-7.68 (m, 1H), 7.22-7.21 (m, 2H), 3.32-3.25 (m, 2H), 2.95-2.89 (m, 4H), 2.06-1.98 (m, 2H), 1.91 (d, 2H, *J* = 6.0 Hz), 1.69-1.61 (m, 4H), 1.17-1.12 (m, 3H); ^{13}C NMR ($CDCl_3$, 75 MHz) δ 161.4, 153.1, 150.1, 129.0, 125.4, 118.9, 118.1, 110.6, 52.7, 31.9, 28.3, 26.9, 25.5, 24.9, 17.4, 12.9; LC-MS: 337.1 (M^+ + 1); elemental analysis ($C_{17}H_{20}O_5S$): calculated C: 60.70%, H: 5.99%, S: 9.53%; found: C: 60.92%, H: 5.84%, S: 9.48%.

*6-Oxo-6,7,8,9,10,11-hexahydrocyclohepta[c]chromen-3-yl cyclopropanesulfonate (**1d**)*

It was purified by flash column chromatography (silica gel, hexanes:ethyl acetate 5:1 v/v); mp: 128-30 ºC; ^{1}H NMR ($CDCl_3$, 400 MHz) δ 7.71 (d, 1H, *J* = 8.4 Hz), 7.27-7.25 (m, 2H), 3.33 (q, 1H, *J* = 7.1 Hz), 2.97-2.92 (m, 4H), 2.69-2.63 (m, 1H), 1.93 (brs, 2H), 1.71-1.63 (m, 4H), 1.25-1.18 (m, 4H); ^{13}C NMR ($CDCl_3$, 100 MHz) δ 161.5, 153.0, 152.8, 150.6, 129.0, 125.3, 118.9, 118.3, 110.8, 41.5, 31.9, 28.2, 26.9, 25.5, 24.9, 14.0, 6.4; LC-MS: 335.1 (M^+ + 1); elemental analysis ($C_{17}H_{18}O_5S$): calculated C: 61.06%, H: 5.43%, S: 9.59%; found: C: 60.89%, H: 5.18%, S: 9.78%.

*6-Oxo-6,7,8,9,10,11-hexahydrocyclohepta[c]chromen-3-yl benzenesulfonate (**1e**)*

It was purified by flash column chromatography (silica gel, hexanes:ethyl acetate 7:1 v/v then switching to hexanes:ethyl acetate 5:1 v/v); mp: 125-8 ºC; ^{1}H NMR ($CDCl_3$, 400 MHz) δ 7.86 (d, 2H, *J* = 7.8 Hz), 7.71 (t, 2H, *J* = 7.4 Hz), 7.63 (d, 1H, *J* = 8.9 Hz), 7.56 (t, 3H, *J* = 7.6 Hz), 2.93-2.87 (m, 4H), 1.90 (d, 2H, *J* = 5.5 Hz), 1.68-1.58 (m, 4H); ^{13}C NMR ($CDCl_3$, 100 MHz) δ 161.4, 152.9, 152.8, 150.5, 135.0, 134.7, 129.4, 129.0, 128.4, 125.2, 118.9, 118.4, 110.7, 31.8, 28.2, 26.8, 25.4, 24.8; LC-MS: 371.1 (M^+ + 1); elemental analysis ($C_{20}H_{18}O_5S$): calculated C: 64.85%, H: 4.90%, S: 8.66%; found: C: 64.62%, H: 4.98%, S: 8.90%.

*6-Oxo-6,7,8,9,10,11-hexahydrocyclohepta[c]chromen-3-yl 4-methylbenzenesulfonate (**1f**)*

It was purified by flash column chromatography (silica gel, hexanes:ethyl acetate 10:1 v/v then switching to hexanes:ethyl acetate 7:1 v/v); mp: 149-52 ºC; ^{1}H NMR ($CDCl_3$, 400 MHz) δ 7.73 (d, 2H, *J* = 7.2 Hz), 7.64-7.62 (m, 1H), 7.35-7.32 (m, 2H), 7.06 (d, 1H, *J* = 8.8 Hz), 6.82 (s, 1H), 2.94-2.88 (m, 4H), 2.47 (s, 3H), 1.91-1.89 (m, 2H), 1.69-1.60 (m, 4H); ^{13}C NMR ($CDCl_3$, 100 MHz) δ 161.5, 152.9, 152.8, 150.6, 146.0, 132.0, 130.0, 128.9, 128.5, 125.2, 118.8, 118.6, 110.7, 31.9, 28.2, 26.8, 25.5, 24.8, 21.8; LC-MS: 385.1 (M^+ + 1); elemental analysis ($C_{21}H_{20}O_5S$): calculated C: 65.61%, H: 5.24%, S: 8.34%; found: C: 65.38%, H: 5.08%, S: 8.58%.

6-Oxo-6,7,8,9,10,11-hexahydrocyclohepta[c]chromen-3-yl 4-(trifluoromethyl)

*benzenesulfonate (**1g**)*

It was purified by flash column chromatography (silica gel, hexanes:ethyl acetate 10:1 v/v); mp: 137-40 ºC; ^{1}H NMR ($CDCl_3$, 300 MHz) δ 8.03 (d, 2H, *J* = 9.0 Hz), 7.85 (d, 2H, *J* = 9.0 Hz), 7.66 (d, 1H, *J* = 9.0 Hz), 7.04-6.95 (m, 2H), 2.95-2.88 (m, 4H), 1.93-1.88 (m, 2H), 1.72-1.59 (m, 4H); ^{13}C NMR ($CDCl_3$, 75 MHz) δ 161.3, 152.9, 152.7, 150.1, 138.7, 136.4, 136.1, 129.3, 129.0, 126.6, 125.4, 119.2, 118.1, 110.7, 31.9, 28.2, 26.9, 25.4, 24.8; LC-MS: 439.1 (M^+ + 1); elemental analysis ($C_{21}H_{17}F_3O_5S$): calculated C: 57.53%, H: 3.91%, S: 7.31%; found: C: 57.44%, H: 3.88%, S: 7.42%.

*6-Oxo-6,7,8,9,10,11-hexahydrocyclohepta[c]chromen-3-yl 4-(tert-butyl) benzenesulfonate (**1h**)*

It was purified by flash column chromatography (silica gel, hexanes:ethyl acetate 20:1 v/v then switching to hexanes:ethyl acetate 15:1 v/v); mp: 94-7 ºC; ^{1}H NMR ($CDCl_3$, 400 MHz) δ 7.79 (d, 2H, *J* = 8.5 Hz), 7.64 (d, 1H, *J* = 8.9 Hz), 7.56 (d, 2H, *J* = 8.5 Hz), 7.08 (dd, 1H, *J* = 2.1 Hz, 8.8 Hz), 6.90 (d, 1H, *J* = 2.2 Hz), 2.91 (dt, 4H, *J* = 10.6 Hz, 9.8 Hz), 1.90 (q, 2H, *J* = 6.0 Hz), 1.69-1.59 (m, 4H), 1.36 (s, 9H); ^{13}C NMR ($CDCl_3$, 100 MHz) δ 161.5, 158.8, 152.9, 152.8, 150.7, 132.2, 128.9, 128.3, 126.4, 125.1, 118.8, 118.5, 110.7, 35.4, 31.9, 31.0, 28.2, 26.8, 25.5, 24.9; LC-MS: 427.2 (M^+ + 1); elemental analysis ($C_{24}H_{26}O_5S$): calculated C: 67.58%, H: 6.14%, S: 7.52%; found: C: 67.80%, H: 6.03%, S: 7.57%.

*6-Oxo-6,7,8,9,10,11-hexahydrocyclohepta[c]chromen-3-yl 4-fluorobenzenesulfonate (**1i**)*

It was purified by flash column chromatography (silica gel, hexanes:ethyl acetate 30:1 v/v then switching to hexanes:ethyl acetate 25:1 v/v); mp: 147-9 ºC; ^{1}H NMR ($CDCl_3$, 400 MHz) δ 7.91-7.88 (m, 2H), 7.64 (d, 1H, *J* = 8.8 Hz), 7.26-7.22 (m, 2H), 7.03 (dd, 1H, *J* = 2.3 Hz, 8.8 Hz), 6.89 (d, 1H, *J* = 2.2 Hz), 2.94-2.88 (m, 4H), 1.90 (q, 2H, *J* = 6.0 Hz), 1.69-1.60 (m, 4H); ^{13}C NMR ($CDCl_3$, 100 MHz) δ 161.4, 152.8, 152.7, 150.4, 131.4, 131.3, 129.1, 125.3, 119.0, 118.3, 117.0, 116.8, 110.7, 31.8, 28.2, 26.8, 25.4, 24.8; LC-MS: 389.1 (M^+ + 1); elemental analysis ($C_{20}H_{17}FO_5S$): calculated C: 61.85%, H: 4.41%, S: 8.25%; found: C: 61.60%, H: 4.50%, S: 8.35%.

*6-Oxo-7,8,9,10,11,12-hexahydro-6H-cycloocta[c]chromen-3-yl ethanesulfonate (**1j**)*

It was purified by flash column chromatography (silica gel, hexanes:ethyl acetate 7:1 v/v then switching to hexanes:ethyl acetate 4:1 v/v); mp: 157-60 ºC; ^{1}H NMR ($CDCl_3$, 400 MHz) δ 7.65 (d, 1H, *J* = 9.4 Hz), 7.26-7.22 (m, 2H), 3.35 (q, 2H, *J* = 7.4 Hz), 2.98 (t, 2H, *J* = 4.6 Hz), 2.81 (t, 2H, *J* = 3.9 Hz), 1.81-1.73 (m, 4H), 1.59-1.44 (m, 7H); ^{13}C NMR ($CDCl_3$, 100 MHz) δ 160.8, 153.3, 149.8, 149.5, 127.2, 125.6, 118.4, 118.1, 110.6, 45.6, 29.7, 29.1, 26.9, 26.6, 26.4, 25.9,

8.3; LC-MS: 337.1 (M^+ + 1); elemental analysis ($C_{17}H_{20}O_5S$): calculated C: 60.70%, H: 5.99%, S: 9.53%; found: C: 60.44%, H: 5.81%, S: 9.66%.

*6-Oxo-7,8,9,10,11,12-hexahydro-6H-cycloocta[c]chromen-3-yl propane-1-sulfonate (**1k**)*

It was purified by flash column chromatography (silica gel, hexanes:ethyl acetate 7:1 v/v); mp: 114-7 ºC; ^{1}H NMR ($CDCl_3$, 400 MHz) δ 7.65 (d, 1H, *J* = 5.9 Hz), 7.25-7.22 (m, 2H), 3.31-3.24 (m, 2H), 2.99 (t, 2H, *J* = 6.0 Hz), 2.82 (t, 2H, *J* = 5.8 Hz), 2.09-1.98 (m, 2H), 1.82-1.73 (m, 4H), 1.53-1.44 (m, 4H), 1.15 (t, 3H, *J* = 7.4 Hz); ^{13}C NMR ($CDCl_3$, 100 MHz) δ 160.8, 153.3, 149.9, 149.6, 127.2, 125.6, 120.9, 118.1, 110.6, 52.7, 29.7, 29.1, 26.9, 26.6, 26.4, 25.9, 17.4, 12.8; LC-MS: 351.1 (M^+ + 1); elemental analysis ($C_{18}H_{22}O_5S$): calculated C: 61.69%, H: 6.33%, S: 9.15%; found: C: 61.48%, H: 6.45%, S: 9.08%.

*6-Oxo-7,8,9,10,11,12-hexahydro-6H-cycloocta[c]chromen-3-yl benzenesulfonate (**1l**)*

It was purified by flash column chromatography (silica gel, hexanes:ethyl acetate 10:1 v/v then switching to hexanes:ethyl acetate 6:1 v/v); mp: 108-11 ºC; ^{1}H NMR ($CDCl_3$, 400 MHz) δ 7.88 (d, 2H, *J* = 8.0 Hz), 7.72 (t, 1H, *J* = 7.0 Hz), 7.57 (t, 3H, *J* = 7.6 Hz), 7.04 (dd, 1H, *J* = 2.1 Hz, 8.8 Hz), 6.85 (d, 1H, *J* = 2.1 Hz), 2.96 (t, 2H, *J* = 6.8 Hz), 2.79 (t, 2H, *J* = 6.1 Hz), 1.81-1.72 (m, 4H), 1.52-1.44 (m, 4H); ^{13}C NMR ($CDCl_3$, 100 MHz) δ 160.8, 153.0, 150.3, 149.6, 135.1, 134.7, 129.4, 128.4, 127.2, 125.5, 118.5, 118.4, 110.8, 29.6, 29.1, 26.9, 26.6, 26.4, 25.9; LC-MS: 385.1 (M^+ + 1); elemental analysis ($C_{21}H_{20}O_5S$): calculated C: 65.61%, H: 5.24%, S: 8.34%; found: C: 65.28%, H: 5.12%, S: 8.58%.

*6-Oxo-7,8,9,10,11,12-hexahydro-6H-cycloocta[c]chromen-3-yl 4-methylbenzenesulfonate (**1m**)*

It was purified by flash column chromatography (silica gel, hexanes:ethyl acetate 12:1 v/v then switching to hexanes:ethyl acetate 6:1 v/v); mp: 107-10 ºC; ^{1}H NMR ($CDCl_3$, 400 MHz) δ 7.74 (d, 2H, *J* = 8.2 Hz), 7.58 (d, 1H, *J* = 8.8 Hz), 7.36-7.29 (m, 2H), 7.05 (dd, 1H, *J* = 1.9 Hz, 8.8

Hz), 6.82 (d, 1H, *J* = 1.9 Hz), 2.95 (t, 2H, *J* = 5.7 Hz), 2.78 (t, 2H, *J* = 5.0 Hz), 2.47 (s, 3H), 1.80-1.71 (m, 4H), 1.50-1.42 (m, 4H); ^{13}C NMR ($CDCl_3$, 100 MHz) δ 160.9, 152.9, 150.4, 149.7, 146.0, 132.0, 130.1, 128.4, 127.1, 125.4, 118.7, 118.3, 110.8, 29.6, 29.1, 26.9, 26.6, 26.4, 25.8, 21.8; LC-MS: 399.2 (M^+ + 1); elemental analysis ($C_{22}H_{22}O_5S$): calculated C: 66.31%, H: 5.56%, S: 8.05%; found: C: 66.09%, H: 5.49%, S: 8.12%.

*6-Oxo-7,8,9,10,11,12-hexahydro-6H-cycloocta[c]chromen-3-yl 4-(tert-butyl) benzenesulfonate (**1n**)*

It was purified by flash column chromatography (silica gel, hexanes:ethyl acetate 20:1 v/v then switching to hexanes:ethyl acetate 15:1 v/v); mp: 166-9 ºC; ^{1}H NMR ($CDCl_3$, 400 MHz) δ 7.81 (d, 2H, *J* = 8.5 Hz), 7.59-7.56 (m, 3H), 7.07 (dd, 1H, *J* = 2.2 Hz, 8.8 Hz), 6.91 (d, 1H, *J* = 2.2 Hz), 2.96 (t, 2H, *J* = 6.4 Hz), 2.79 (t, 2H, *J* = 6.0 Hz), 1.83-1.69 (m, 4H), 1.52-1.43 (m, 4H), 1.36 (s, 9H); ^{13}C NMR ($CDCl_3$, 75 MHz) δ 160.9, 158.8, 153.0, 150.5, 149.7, 132.2, 128.3, 127.1, 126.5, 125.4, 118.6, 118.3, 110.7, 35.4, 31.0, 29.7, 29.1, 26.9, 26.6, 26.4, 25.9; LC-MS: 441.2 (M^+ + 1); elemental analysis ($C_{25}H_{28}O_5S$): calculated C: 68.16%, H: 6.41%, S: 7.28%; found: C: 67.84%, H: 6.60%, S: 7.13%.

*2-Methoxy-6-oxo-6,7,8,9,10,11-hexahydrocyclohepta[c]chromen-3-yl methanesulfonate (**1o**)*

It was purified by flash column chromatography (silica gel, hexanes:ethyl acetate 10:1 v/v then switching to 3.5:1 v/v); yield: 85%; ^{1}H NMR ($CDCl_3$, 400 MHz) δ 7.30 (s, 1H), 7.17 (s, 1H), 3.79 (s, 3H), 3.25 (s, 3H), 2.94-2.90 (m, 4H), 1.95-1.90 (m, 2H), 1.73-1.62 (m, 4H); ^{13}C NMR ($CDCl_3$, 100 MHz) δ 161.5, 152.3, 148.4, 146.5, 139.7, 129.6, 119.2, 113.3, 107.0, 56.7, 38.8, 31.8, 28.4, 26.9, 25.5, 24.9; LC-MS: 339.0 (M^+ + 1); elemental analysis ($C_{16}H_{18}O_6S$): calculated C: 56.79%, H: 5.36%, S: 9.47%; found: C: 56.83%, H: 5.30%, S: 9.60%.

*2-Methoxy-6-oxo-7,8,9,10,11,12-hexahydro-6H-cycloocta[c]chromen-3-yl methanesulfonate (**1p**)*

It was purified by flash column chromatography (silica gel, hexanes:ethyl acetate 10:1 v/v then switching to 3.5:1 v/v); yield: 85%; ^{1}H NMR ($CDCl_3$, 300 MHz) δ 7.30 (s, 1H), 7.11 (s, 1H), 3.96 (s, 3H), 3.26 (s, 3H), 2.98 (t, 2H, *J* = 6.0 Hz), 2.82 (t, 2H, *J* = 6.0 Hz), 1.84-1.74 (m, 4H), 1.53-1.44 (m, 4H); ^{13}C NMR ($CDCl_3$, 75 MHz) δ 161.0, 149.0, 148.5, 146.7, 139.6, 127.9, 118.7, 113.4, 107.1, 56.7, 38.8, 29.7, 29.1, 27.0, 26.8, 26.6, 25.9; LC-MS: 353.0 (M^+ + 1); elemental analysis ($C_{17}H_{20}O_6S$): calculated C: 57.94%, H: 5.72%, S: 9.10%; found: C: 57.69%, H: 5.87%, S: 8.90%.

*2-Methoxy-6-oxo-6,7,8,9,10,11-hexahydrocyclohepta[c]chromen-3-yl propane-1-sulfonate (**1q**)*

It was purified by flash column chromatography (silica gel, hexanes:ethyl acetate 10:1 v/v then switching to 6:1 v/v); yield: 95%; ^{1}H NMR ($CDCl_3$, 400 MHz) δ 7.29 (s, 1H), 7.16 (s, 1H), 3.96 (s, 3H), 3.35-3.31 (m, 2H), 2.94-2.89 (m, 4H), 2.08-2.00 (m, 2H), 1.95-1.91 (m, 2H), 1.73-1.67 (m, 2H), 1.64-1.58 (m, 2H), 1.13 (t, 3H, *J* = 7.5 Hz); ^{13}C NMR ($CDCl_3$, 100 MHz) δ 161.6, 152.5, 148.5, 146.5, 139.8, 129.4, 119.0, 113.1, 107.0, 56.7, 31.8, 28.4, 26.9, 25.5, 24.9, 17.4, 12.9; LC-MS: 367.0 (M^+ + 1); elemental analysis ($C_{18}H_{22}O_6S$): calculated C: 59.00%, H: 6.05%, S: 8.75%; found: C: 58.65%, H: 6.31%, S: 8.53%.

*2-Methoxy-6-oxo-7,8,9,10,11,12-hexahydro-6H-cycloocta[c]chromen-3-yl propane-1-sulfonate (**1r**)*

It was purified by flash column chromatography (silica gel, hexanes:ethyl acetate 10:1 v/v then switching to 6:1 v/v); yield: 91%; ^{1}H NMR ($CDCl_3$, 400 MHz) δ 7.29 (s, 1H), 7.10 (s, 1H), 3.95 (s, 3H), 3.34 (t, 2H, *J* = 7.7 Hz), 2.97 (t, 2H, *J* = 6.4 Hz), 2.80 (t, 2H, *J* = 6.0 Hz), 2.10-1.97 (m, 2H), 1.86-1.73 (m, 4H), 1.53-1.44 (m, 4H), 1.12 (t, 3H, *J* = 6.9 Hz); ^{13}C NMR ($CDCl_3$, 100 MHz) δ 161.0, 149.2, 146.6, 139.6, 127.6, 121.8, 118.4, 113.1, 107.1, 56.7, 53.6, 29.7, 29.0,

26.9, 26.7, 26.5, 25.8, 17.4, 12.9; LC-MS: 381.0 (M^+ + 1); elemental analysis ($C_{19}H_{24}O_6S$): calculated C: 59.98%, H: 6.36%, S: 8.43%; found: C: 59.67%, H: 6.62%, S: 8.30%.

*2-Methoxy-6-oxo-6,7,8,9,10,11-hexahydrocyclohepta[c]chromen-3-yl benzenesulfonate (**1s**)*

It was purified by flash column chromatography (silica gel, hexanes:ethyl acetate 7:1 v/v then switching to 5:1 v/v); yield: 92%; ^{1}H NMR ($CDCl_3$, 300 MHz) δ 7.92-7.89 (m, 2H), 7.73-7.68 (m, 1H), 7.58-7.53 (m, 3H), 7.04 (d, 2H, *J* = 3.0 Hz), 3.70 (s, 3H), 2.91-2.88 (m, 4H), 1.90 (q, 2H, *J* = 6.0 Hz), 1.70-1.59 (m, 4H); ^{13}C NMR ($CDCl_3$, 75 MHz) δ 161.7, 152.5, 148.9, 146.2, 140.0, 136.0, 134.4, 129.4, 129.1, 128.5, 119.1, 112.6, 106.8, 56.4, 31.8, 28.4, 26.9, 25.5, 24.9; LC-MS: 401.0 (M^+ + 1); elemental analysis ($C_{21}H_{20}O_6S$): calculated C: 62.99%, H: 5.03%, S: 8.01%; found: C: 62.71%, H: 4.90%, S: 8.12%.

*2-Methoxy-6-oxo-7,8,9,10,11,12-hexahydro-6H-cycloocta[c]chromen-3-yl benzenesulfonate (**1t**)*

It was purified by flash column chromatography (silica gel, hexanes:ethyl acetate 10:1 v/v then switching to hexane:ethyl acetate 5:1 v/v); yield: 87%; ^{1}H NMR ($CDCl_3$, 400 MHz) δ 7.93 (d, 2H, *J* = 7.5 Hz), 7.71 (t, 1H, *J* = 7.5 Hz), 7.59-7.55 (m, 2H), 7.00 (brs, 2H), 3.72 (s, 3H), 2.94 (t, 2H, *J* = 6.3 Hz), 2.81 (t, 2H, *J* = 5.9 Hz), 1.81-1.73 (m, 4H), 1.53-1.44 (m, 4H); ^{13}C NMR ($CDCl_3$, 100 MHz) δ 161.1, 149.2, 149.0, 146.4, 139.9, 134.4, 129.3, 129.1, 128.9, 128.5, 127.7, 118.5, 112.6, 107.0, 56.4, 29.7, 29.0, 26.9, 26.7, 26.5, 25.9; LC-MS: 415.0 (M^+ + 1); elemental analysis ($C_{22}H_{22}O_6S$): calculated C: 63.75%, H: 5.35%, S: 7.74%; found: C: 63.89%, H: 5.15%, S: 7.85%.

*2-Methoxy-6-oxo-6,7,8,9,10,11-hexahydrocyclohepta[c]chromen-3-yl 4-methylbenzenesulfonate (**1u**)*

It was purified by flash column chromatography (silica gel, hexanes:ethyl acetate 10:1 v/v then switching to 6:1 v/v); yield: 96%; ^{1}H NMR ($CDCl_3$, 300 MHz) δ 7.80-7.74 (m, 2H), 7.36-7.25

(m, 2H), 7.06-6.94 (m, 2H), 3.76 (s, 3H), 2.91-2.88 (m, 4H), 2.48 (s, 3H), 1.89 (t, 2H, *J* = 6.0 Hz), 1.68-1.61 (m, 4H); ^{13}C NMR ($CDCl_3$, 75 MHz) δ 162.3, 161.7, 152.6, 149.0, 146.2, 145.7, 140.2, 133.0, 129.8, 129.4, 128.5, 119.0, 112.4, 106.9, 56.5, 31.9, 28.4, 26.9, 25.5, 24.9, 21.8; LC-MS: 415.0 (M^+ + 1); elemental analysis ($C_{22}H_{22}O_6S$): calculated C: 63.75%, H: 5.35%, S: 7.74%; found: C: 63.54%, H: 5.43%, S: 7.81%.

*2-Methoxy-6-oxo-7,8,9,10,11,12-hexahydro-6H-cycloocta[c]chromen-3-yl 4-methylbenzenesulfonate (**1v**)*

It was purified by flash column chromatography (silica gel, hexanes:ethyl acetate 10:1 v/v then switching to hexane:ethyl acetate 5:1 v/v); yield: 90%; ^{1}H NMR ($CDCl_3$, 400 MHz) δ 7.80 (d, 2H, *J* = 8.0 Hz), 7.68-7.66 (m, 1H), 7.36-7.33 (m, 2H), 7.02 (s, 1H), 6.94 (s, 1H), 3.76 (s, 3H), 2.95 (t, 2H, *J* = 6.0 Hz), 2.81 (t, 2H, *J* = 5.6 Hz), 2.48 (s, 3H), 1.81-1.73 (m, 4H), 1.53-1.44 (m, 4H); ^{13}C NMR ($CDCl_3$, 100 MHz) δ 161.1, 149.3, 145.6, 140.0, 133.1, 129.8, 129.5, 127.5, 122.0, 118.4, 112.4, 107.0, 56.5, 29.7, 29.0, 26.9, 26.7, 26.5, 25.9, 21.8; LC-MS: 429.2 (M^+ + 1); elemental analysis ($C_{23}H_{24}O_6S$): calculated C: 64.47%, H: 5.65%, S: 7.48%; found: C: 64.20%, H: 5.46%, S: 7.75%.

*2-Methoxy-6-oxo-6,7,8,9,10,11-hexahydrocyclohepta[c]chromen-3-yl 4-(trifluoromethyl)benzenesulfonate (**1w**)*

It was purified by flash column chromatography (silica gel, hexanes:ethyl acetate 10:1 v/v then switching to 6:1 v/v); yield: 87%; ^{1}H NMR ($CDCl_3$, 400 MHz) δ 8.05 (d, 2H, *J* = 7.7 Hz), 7.83 (d, 2H, *J* = 7.6 Hz), 7.16 (s, 1H), 7.04 (s, 1H), 3.64 (s, 3H), 2.89 (brs, 4H), 1.91 (d, 2H, *J* = 4.0 Hz), 1.69-1.61 (m, 4H); ^{13}C NMR ($CDCl_3$, 100 MHz) δ 161.5, 152.3, 148.4, 146.3, 139.4, 136.0, 135.7, 129.7, 129.1, 126.2, 126.1, 119.3, 112.8, 106.7, 56.1, 31.8, 28.4, 26.9, 25.4, 24.8; LC-MS: 469.1 (M^+ + 1); elemental analysis ($C_{22}H_{19}F_3O_6S$): calculated C: 56.41%, H: 4.09%, S: 6.84%; found: C: 56.27%, H: 4.22%, S: 6.60%.

*2-Methoxy-6-oxo-7,8,9,10,11,12-hexahydro-6H-cycloocta[c]chromen-3-yl 4-(trifluoromethyl)benzenesulfonate (**1x**)*

It was purified by flash column chromatography (silica gel, hexanes:ethyl acetate 10:1 v/v then switching to 6:1 v/v); yield: 85%; ^{1}H NMR ($CDCl_3$, 400 MHz) δ 8.07 (d, 2H, *J* = 8.1 Hz), 7.84 (d, 2H, *J* = 8.2 Hz), 7.14 (s, 1H), 6.99 (s, 1H), 3.65 (s, 3H), 2.94 (t, 2H, *J* = 6.0 Hz), 2.81 (t, 2H, *J* = 5.8 Hz), 1.81-1.74 (m, 4H), 1.53-1.44 (m, 4H); ^{13}C NMR ($CDCl_3$, 100 MHz) δ 160.9, 149.0, 148.5, 146.4, 139.7, 139.4, 136.1, 135.7, 129.1, 128.0, 126.2, 118.8, 112.8, 106.8, 56.2, 29.6, 29.0, 26.9, 26.7, 26.6, 25.8; LC-MS: 483.1 (M^+ + 1); elemental analysis ($C_{23}H_{21}F_3O_6S$): calculated C: 57.26%, H: 4.39%, S: 6.65%; found: C: 57.01%, H: 4.22%, S: 6.82%.

*2-Chloro-6-oxo-6,7,8,9,10,11-hexahydrocyclohepta[c]chromen-3-yl methanesulfonate (**1y**)*

It was purified by flash column chromatography (silica gel, hexanes:ethyl acetate 10:1 v/v then switching to 3.5:1 v/v); yield: 85%; ^{1}H NMR ($CDCl_3$, 300 MHz) δ 7.76 (s, 1H), 7.43 (s, 1H), 3.30 (s, 3H), 2.92-2.90 (m, 4H), 1.93-1.91 (m, 2H), 1.70-1.61 (m, 4H); ^{13}C NMR ($CDCl_3$, 75 MHz) δ 162.3, 151.6, 151.3, 145.7, 130.4, 125.6, 122.6, 120.0, 113.0, 39.1, 31.8, 28.3, 27.0, 25.4, 24.8; LC-MS: 343.1 (M^+ + 1); elemental analysis ($C_{15}H_{15}ClO_5S$): calculated C: 52.56%, H: 4.41%, S: 9.35%; found: C: 52.18%, H: 4.62%, S: 9.57%.

*2-Chloro-6-oxo-6,7,8,9,10,11-hexahydrocyclohepta[c]chromen-3-yl benzenesulfonate (**1z**)*

It was purified by flash column chromatography (silica gel, hexanes:ethyl acetate 13:1 v/v then switching to 10:1 v/v); yield: 85%; ^{1}H NMR ($CDCl_3$, 300 MHz) δ 7.94 (d, 2H, *J* = 6.0 Hz), 7.86-7.66 (m, 4H), 7.18 (s, 1H), 2.91-2.89 (m, 4H), 1.92-1.90 (m, 2H), 1.66-1.62 (m, 6H); ^{13}C NMR ($CDCl_3$, 75 MHz) δ 161.0, 151.8, 151.1, 146.2, 135.4, 134.9, 130.2, 129.5, 129.4, 128.6, 125.5, 123.4, 119.7, 112.5, 31.8, 28.3, 27.0, 25.4, 24.8; LC-MS: 405.0 (M^+ + 1); elemental analysis ($C_{20}H_{17}ClO_5S$): calculated C: 59.33%, H: 4.23%, S: 7.92%; found: C: 59.20%, H: 4.40%, S: 7.74%.

*2-Chloro-6-oxo-6,7,8,9,10,11-hexahydrocyclohepta[c]chromen-3-yl 4-methylbenzenesulfonate (**1za**)*

It was purified by flash column chromatography (silica gel, hexanes:ethyl acetate 13:1 v/v then switching to hexane:ethyl acetate 10:1 v/v); yield: 86%; ^{1}H NMR ($CDCl_3$, 300 MHz) δ 7.81 (d, 2H, *J* = 9.0 Hz), 7.66 (s, 1H), 7.37 (d, 2H, *J* = 9.0 Hz), 7.15 (s, 1H), 2.89 (q, 4H, *J* = 6.0 Hz), 2.48 (s, 3H), 1.95-1.87 (m, 2H), 1.71-1.57 (m, 4H); ^{13}C NMR ($CDCl_3$, 75 MHz) δ 161.0, 151.8, 151.0, 146.4, 146.3, 132.4, 130.1, 129.9, 128.6, 128.5, 125.5, 123.5, 119.6, 112.4, 31.8, 28.3, 27.0, 25.4, 24.8, 21.8; LC-MS: 418.9 (M^+ + 1); elemental analysis ($C_{21}H_{19}ClO_5S$): calculated C: 60.21%, H: 4.57%, S: 7.65%; found: C: 60.14%, H: 4.82%, S: 7.48%.

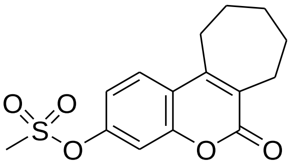

1a

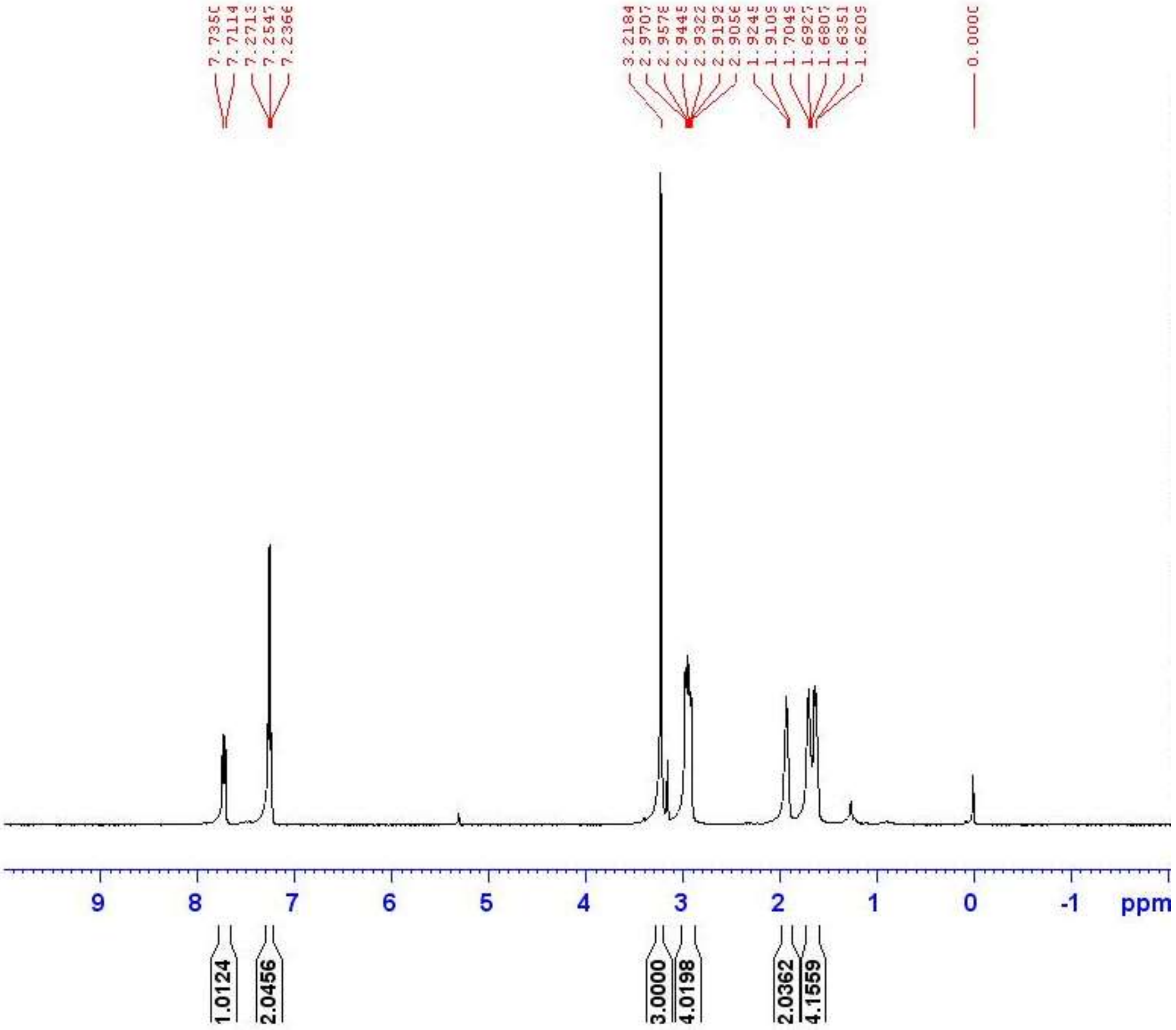
7.7350
7.7114
7.2713
7.2547
7.2366
3.2184
2.9707
2.9578
2.9445
2.9322
2.9192
2.9056
1.9249
1.9109
1.7049
1.6927
1.6807
1.6351
1.6209
0.0000
9 8 7 6 5 4 3 2 1 0 -1 ppm
1.0124
2.0456
3.0000
4.0198
2.0362
4.1559

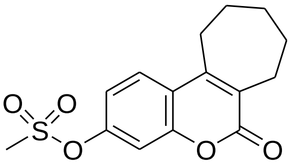

1a

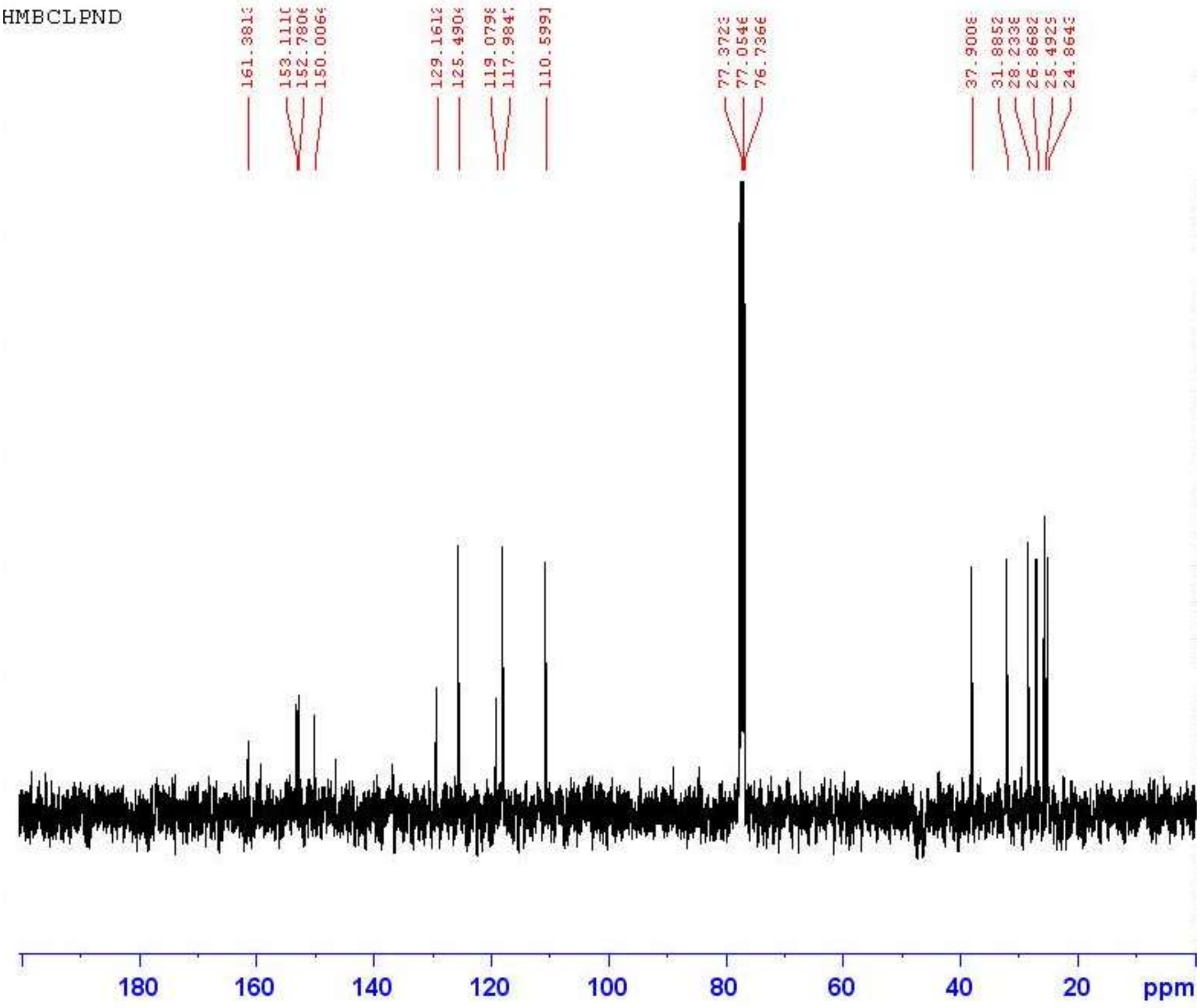
HMBCLPND
161.3813
153.1110
152.7806
150.0064
129.1612
125.4904
119.0798
117.9847
110.5991
77.3723
77.0546
76.7366
37.9006
31.8852
28.2336
26.8682
25.4925
24.8643
180
160
140
120
100
80
60
40
20
ppm

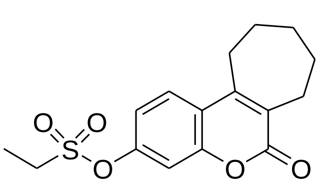
O
S
O
O
O
O

1b

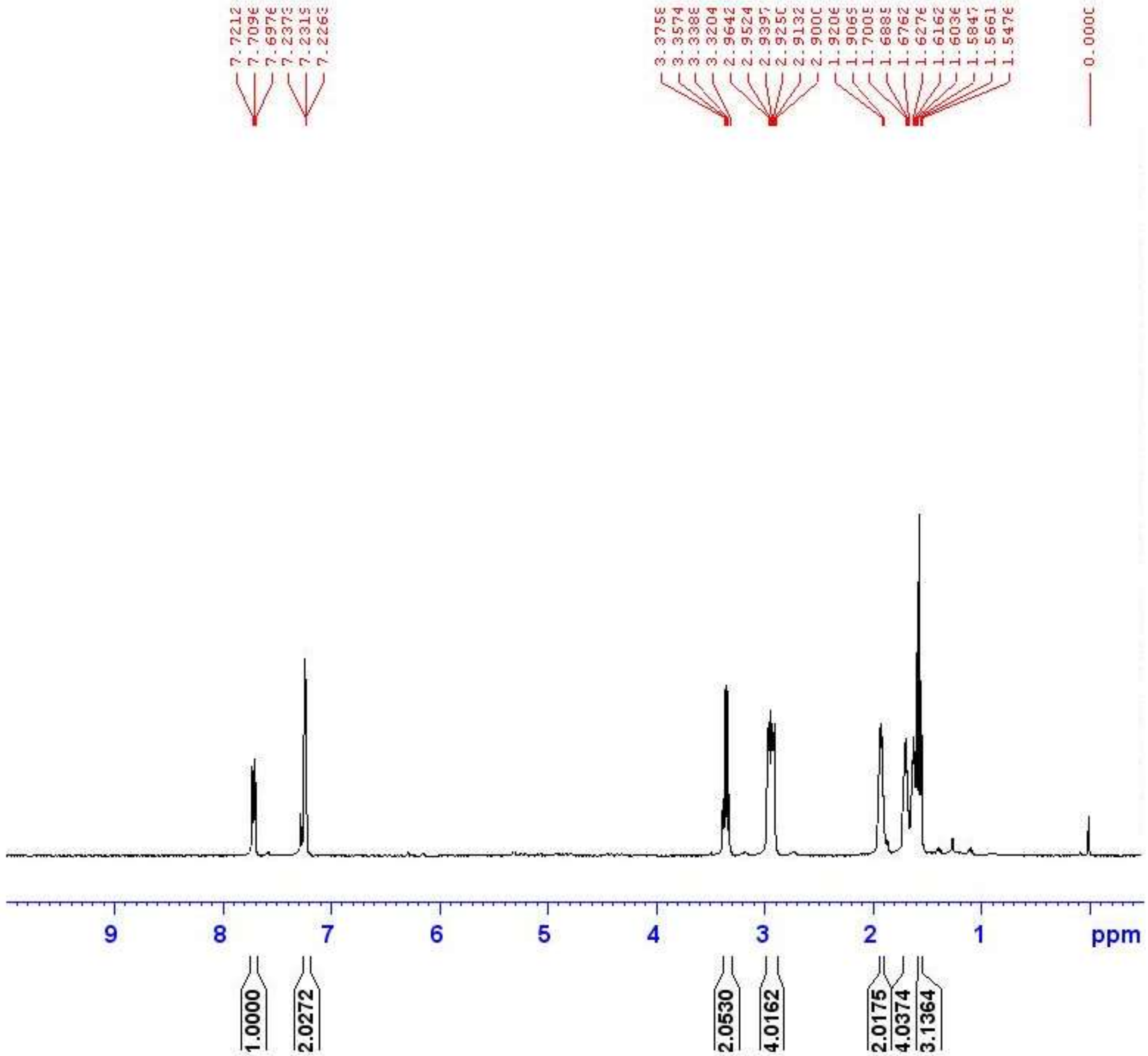
9
8
7
6
5
4
3
2
1
ppm
1.0000
2.0272
2.0530
4.0162
2.0175
4.0374
3.1364

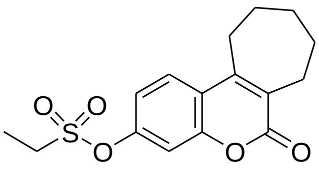

O
O
S
O
O
O

1b

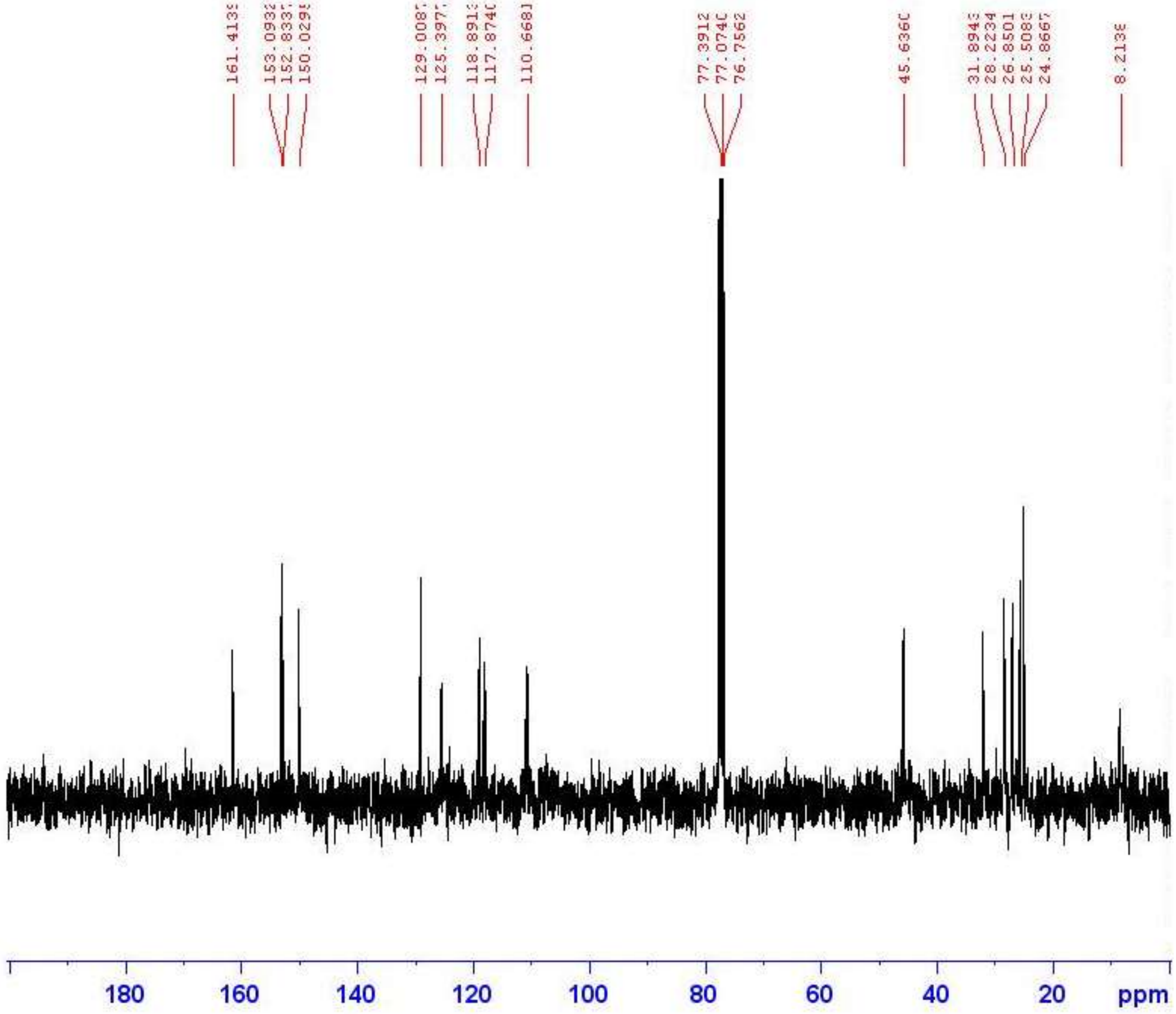

161.413
153.093
152.833
150.029
129.008
125.397
118.891
117.874
110.668
77.3912
77.074
76.7562
45.636
31.894
28.2234
26.8501
25.508
24.866
8.213
180
160
140
120
100
80
60
40
20
ppm

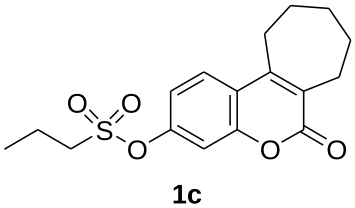
O
S
O
O
O
O

1c

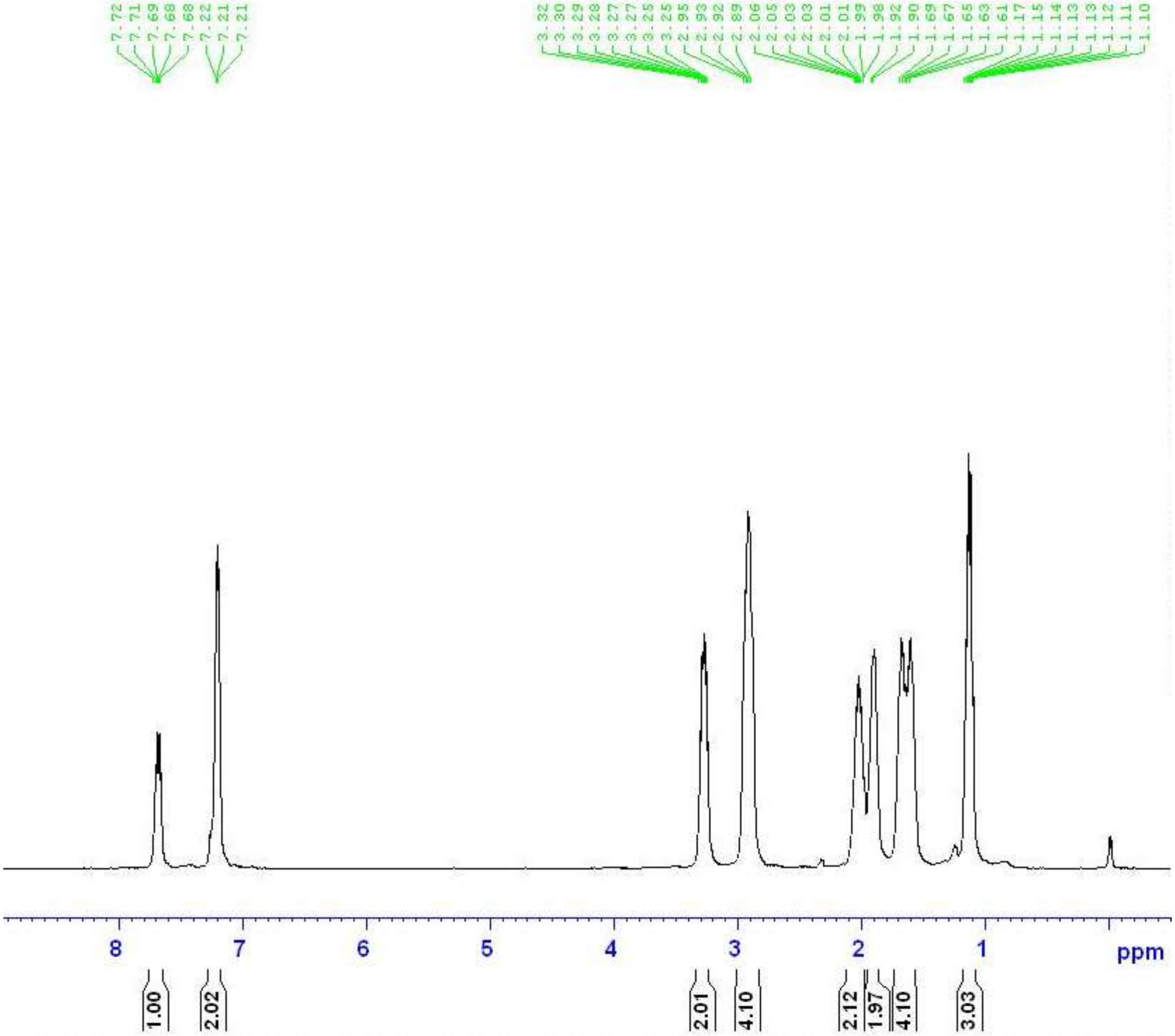
7.72
7.71
7.69
7.68
7.22
7.21
7.21
3.32
3.30
3.29
3.28
3.27
3.27
3.25
3.25
2.95
2.93
2.92
2.89
2.06
2.05
2.03
2.03
2.01
2.01
1.99
1.98
1.92
1.90
1.69
1.67
1.65
1.63
1.61
1.17
1.15
1.14
1.13
1.13
1.12
1.11
1.10
8
7
6
5
4
3
2
1
ppm
1.00
2.02
2.01
4.10
2.12
1.97
4.10
3.03

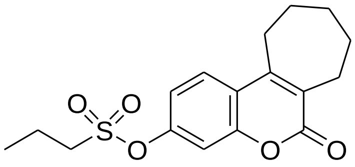

1c

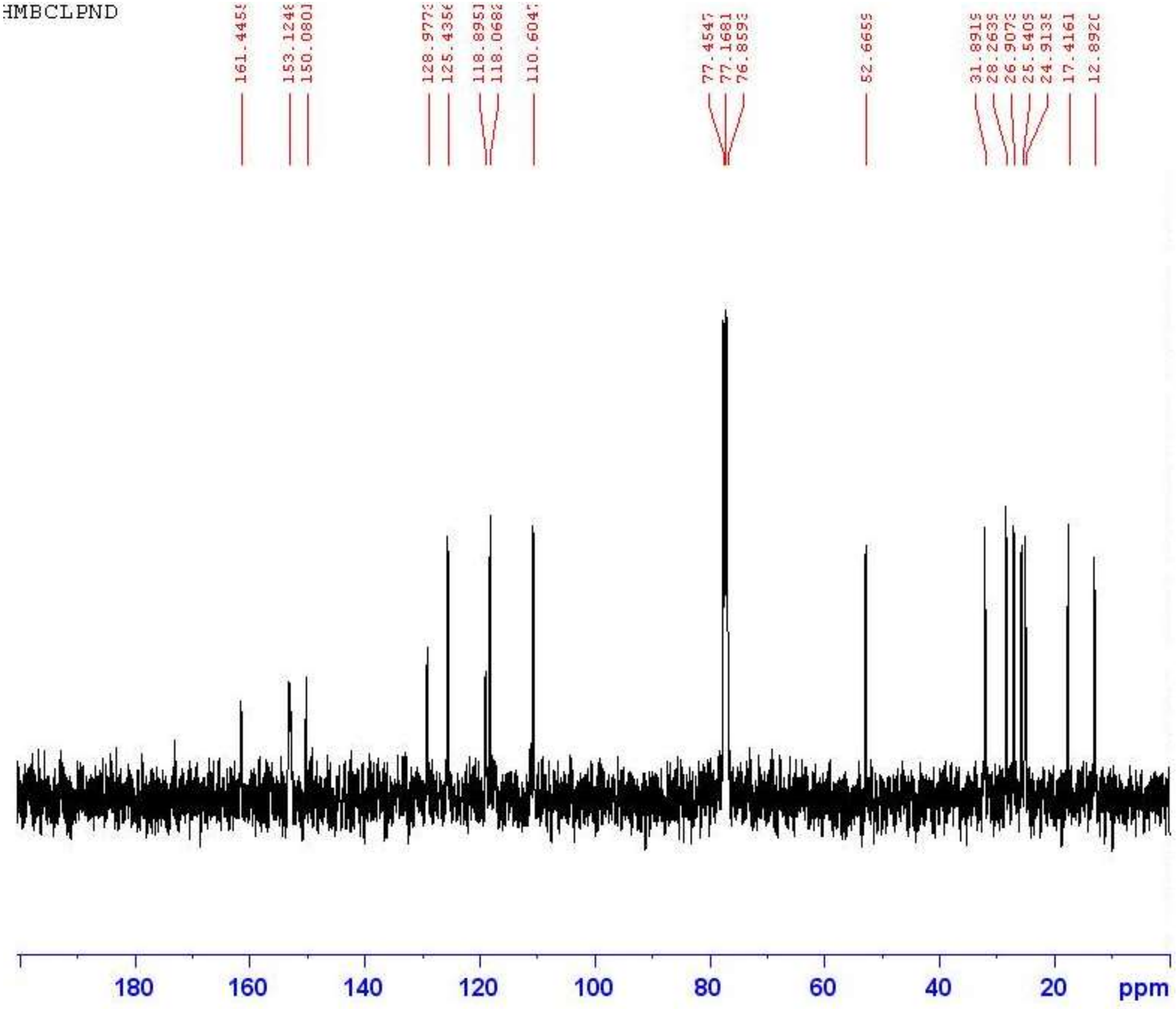
HMBCLPND
161.4455
153.1248
150.0801
128.9773
125.4356
118.8951
118.0682
110.6047
77.4547
77.1681
76.8593
52.6655
31.8915
28.2635
26.9075
25.5405
24.9135
17.4161
12.8920
180
160
140
120
100
80
60
40
20
ppm

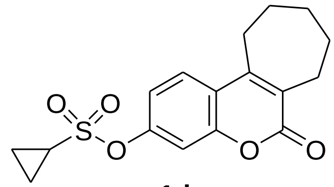

1d

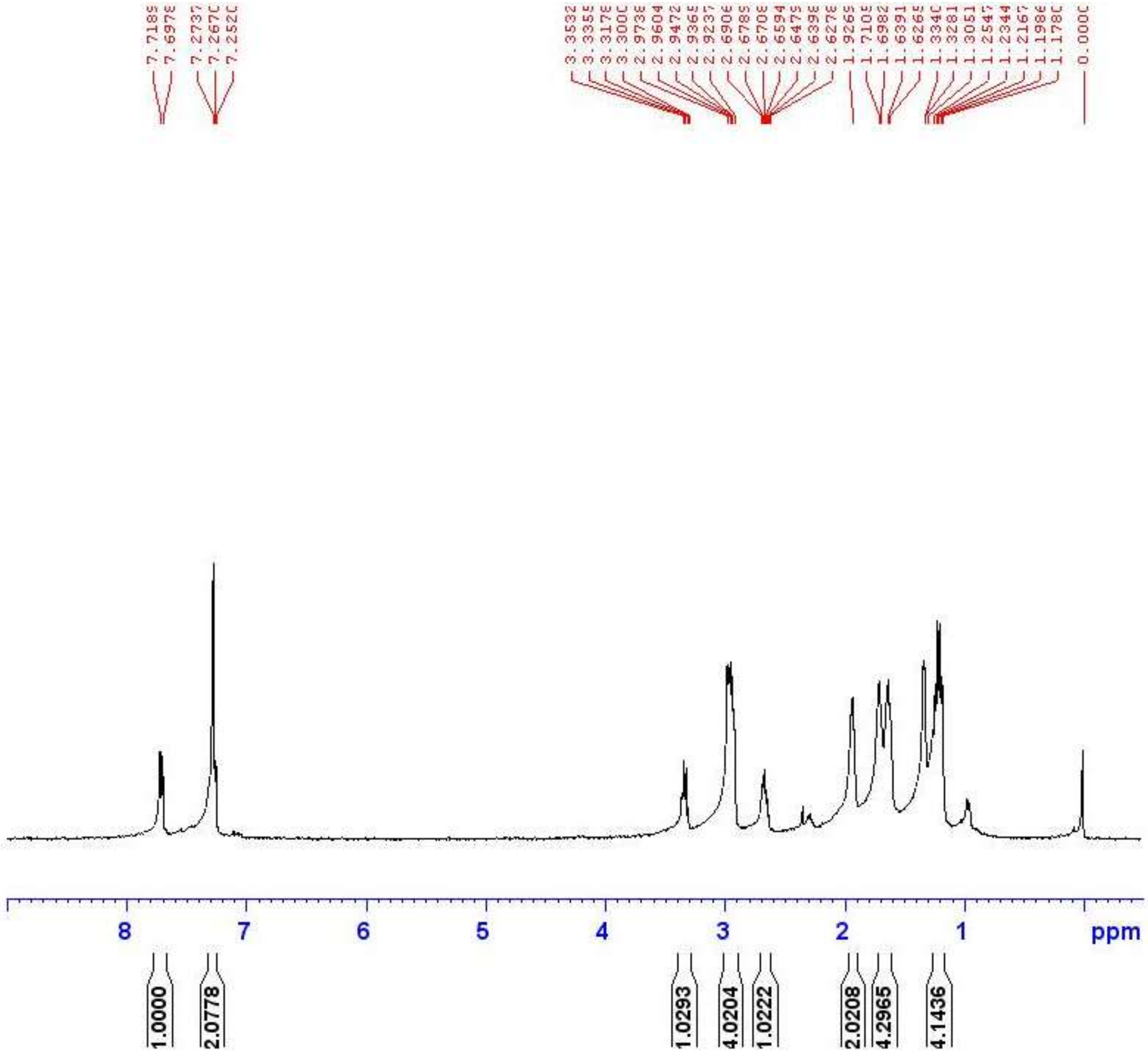
8
7
6
5
4
3
2
1
ppm
1.0000
2.0778
1.0293
4.0204
1.0222
2.0208
4.2965
4.1436

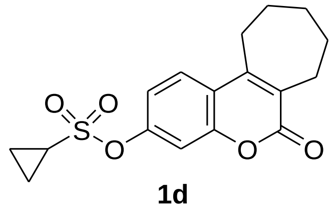

1d

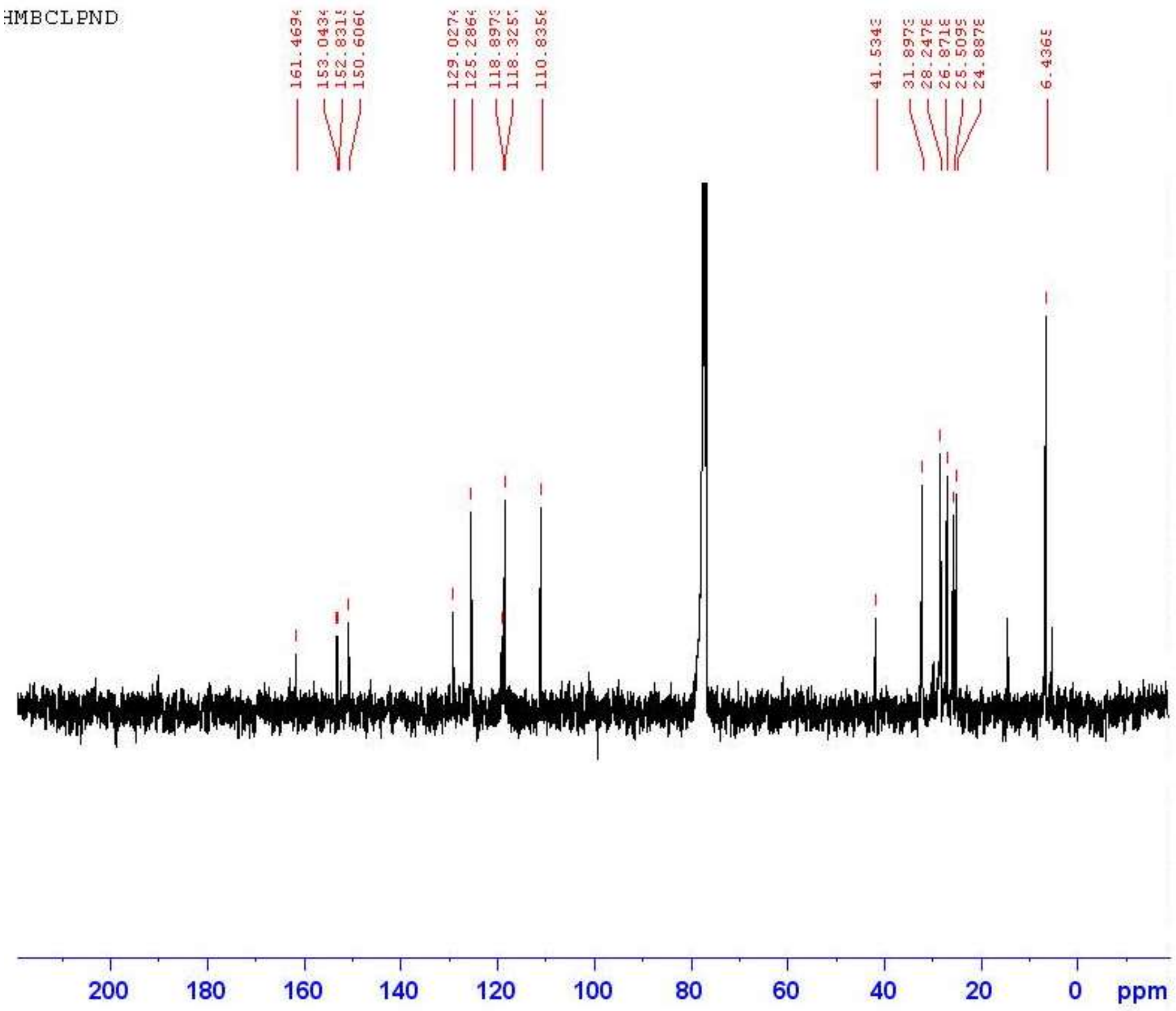
HMBCLPND
161.4694
153.0434
152.8315
150.6060
129.0274
125.2864
118.8973
118.3257
110.8356
41.5343
31.8973
28.2478
26.8718
25.5099
24.8878
6.4369
200
180
160
140
120
100
80
60
40
20
0
ppm

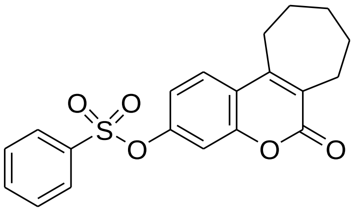

1e

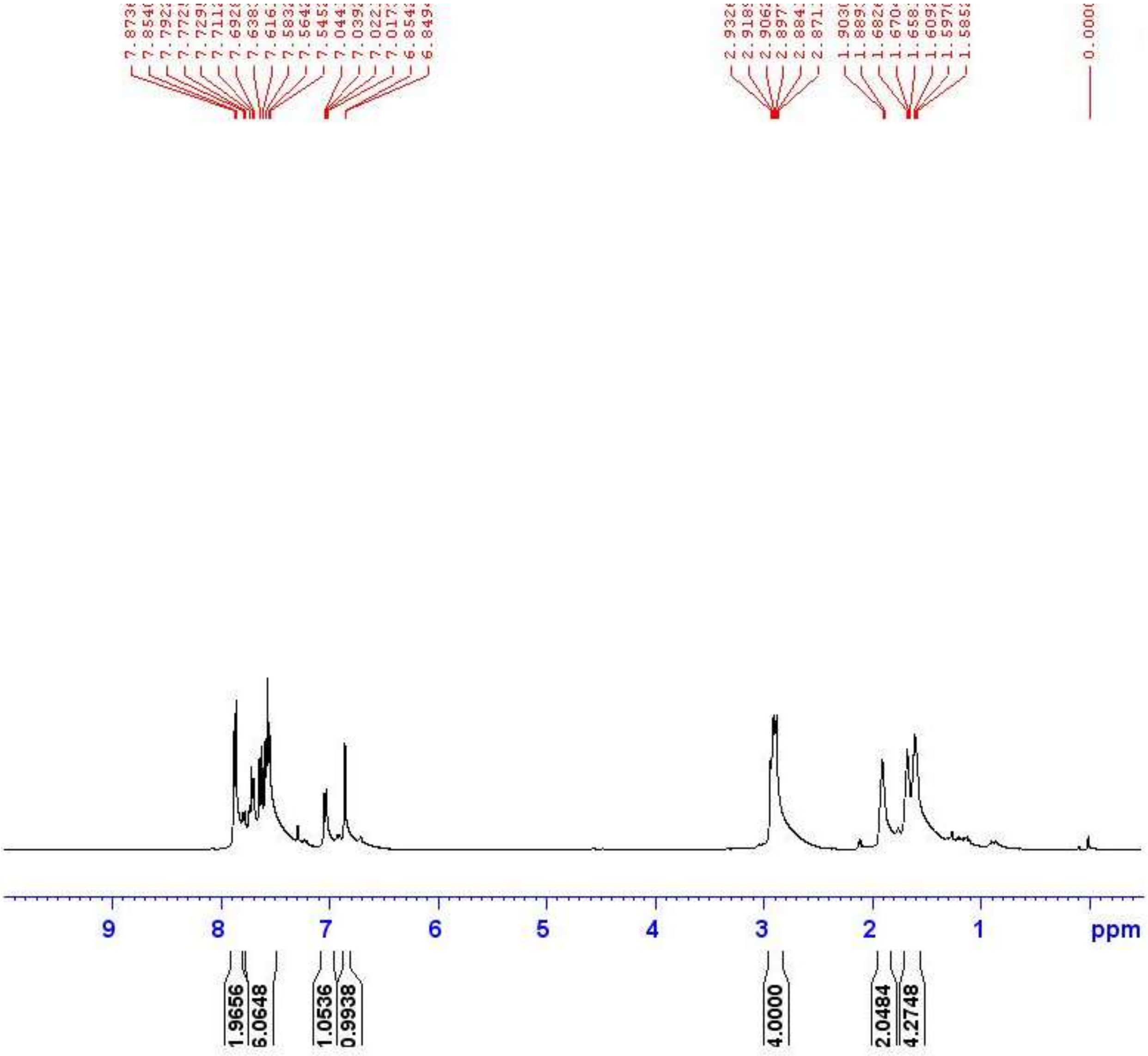

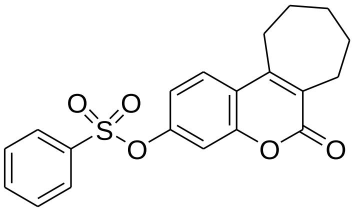

1e

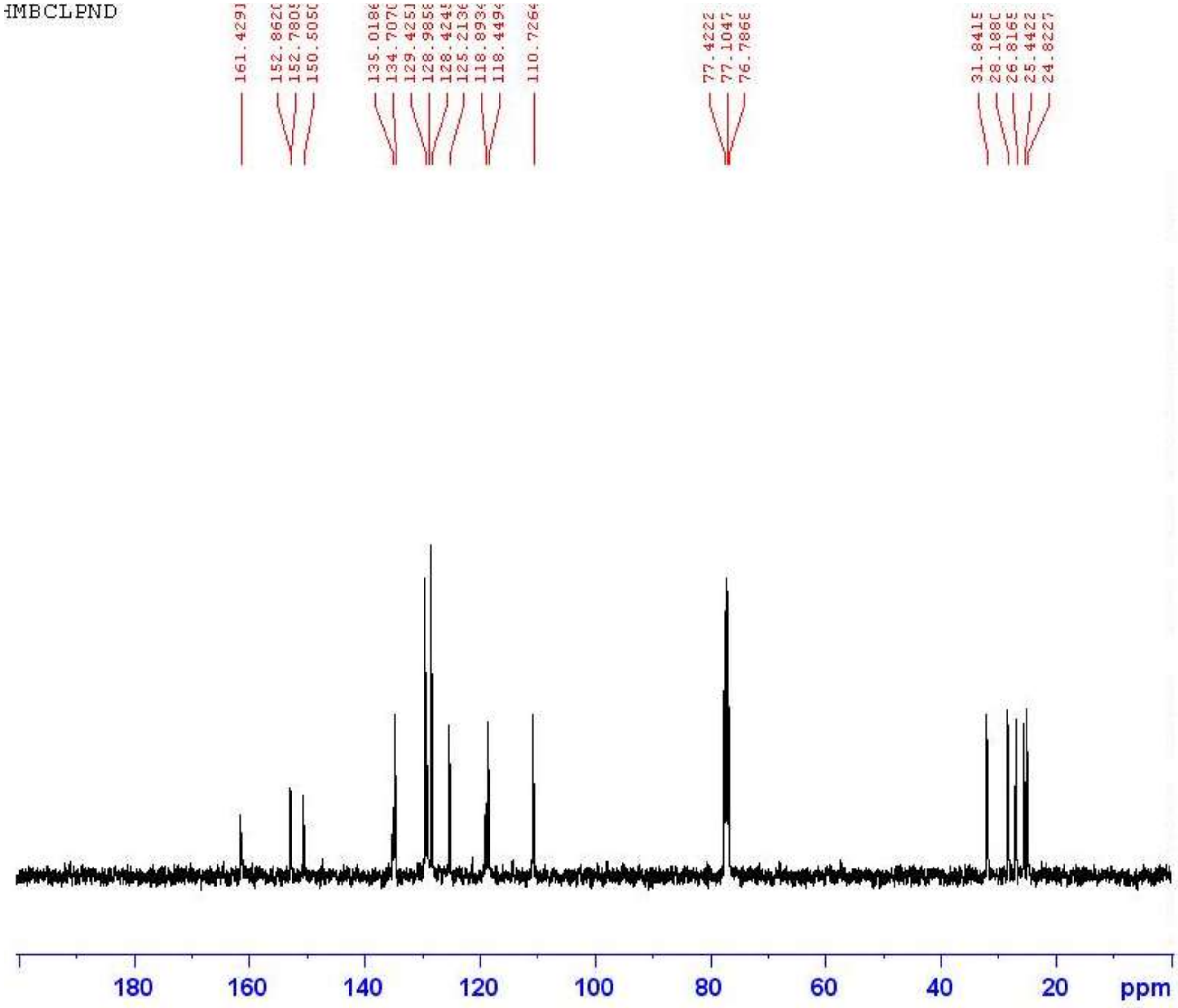
HMBCLPND
161.4293
152.8620
152.7805
150.5050
135.0186
134.7070
129.4251
128.9858
128.4245
125.2136
118.8934
118.4494
110.7264
77.4222
77.1047
76.7868
31.8415
28.1880
26.8165
25.4422
24.8227
180
160
140
120
100
80
60
40
20
ppm

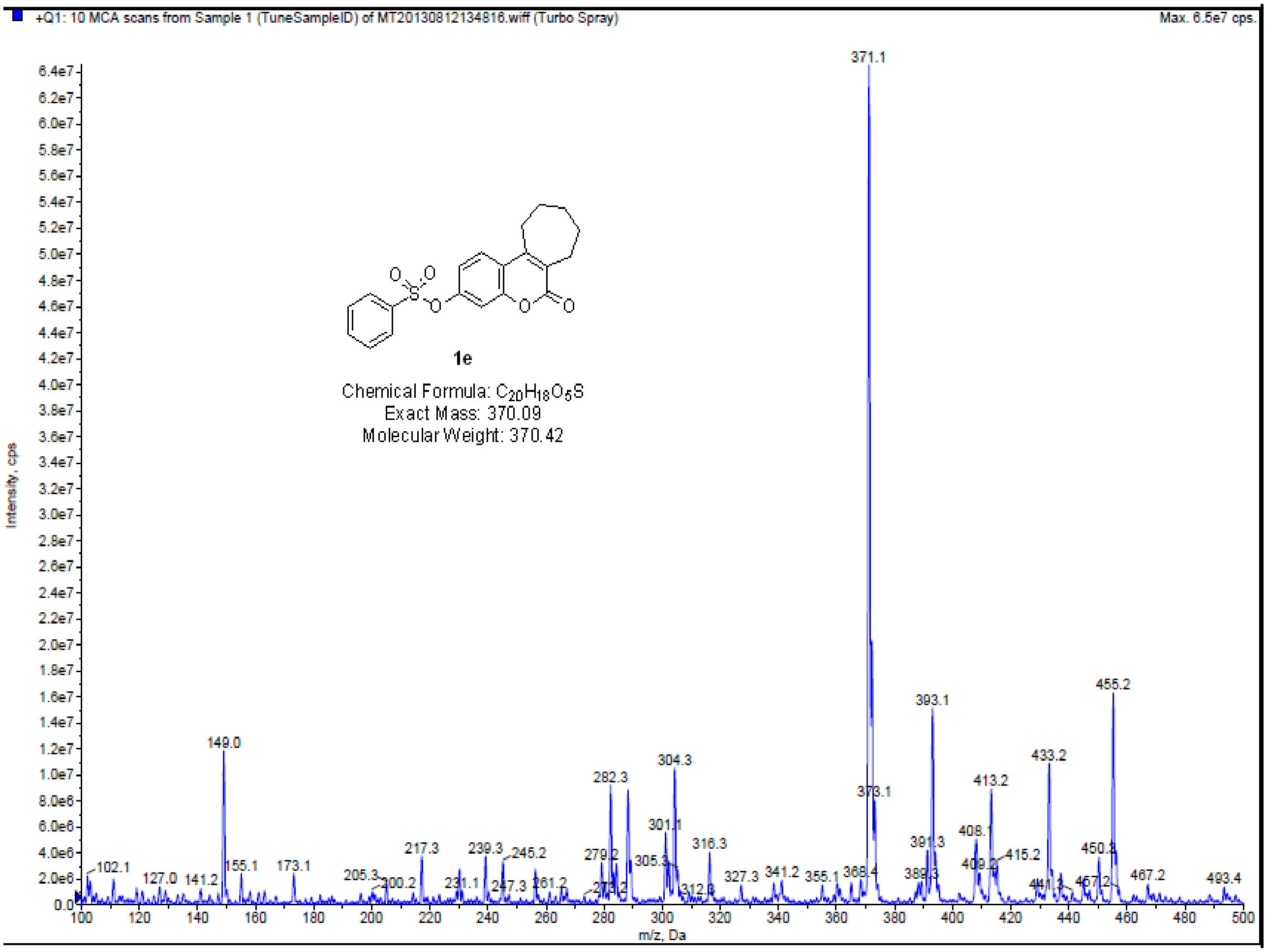
+Q1: 10 MCA scans from Sample 1 (TuneSampleID) of MT20130812134816.wiff (Turbo Spray)
Max. 6.5e7 cps.
Intensity, cps
m/z, Da
371.1
393.1
455.2
433.2
413.2
149.0
304.3
282.3
1e
Chemical Formula: $C_{20}H_{18}O_5S$
Exact Mass: 370.09
Molecular Weight: 370.42

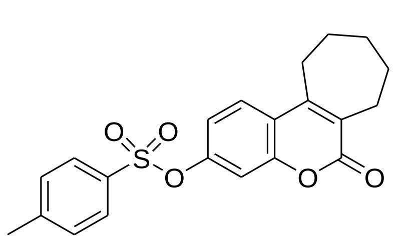

1f

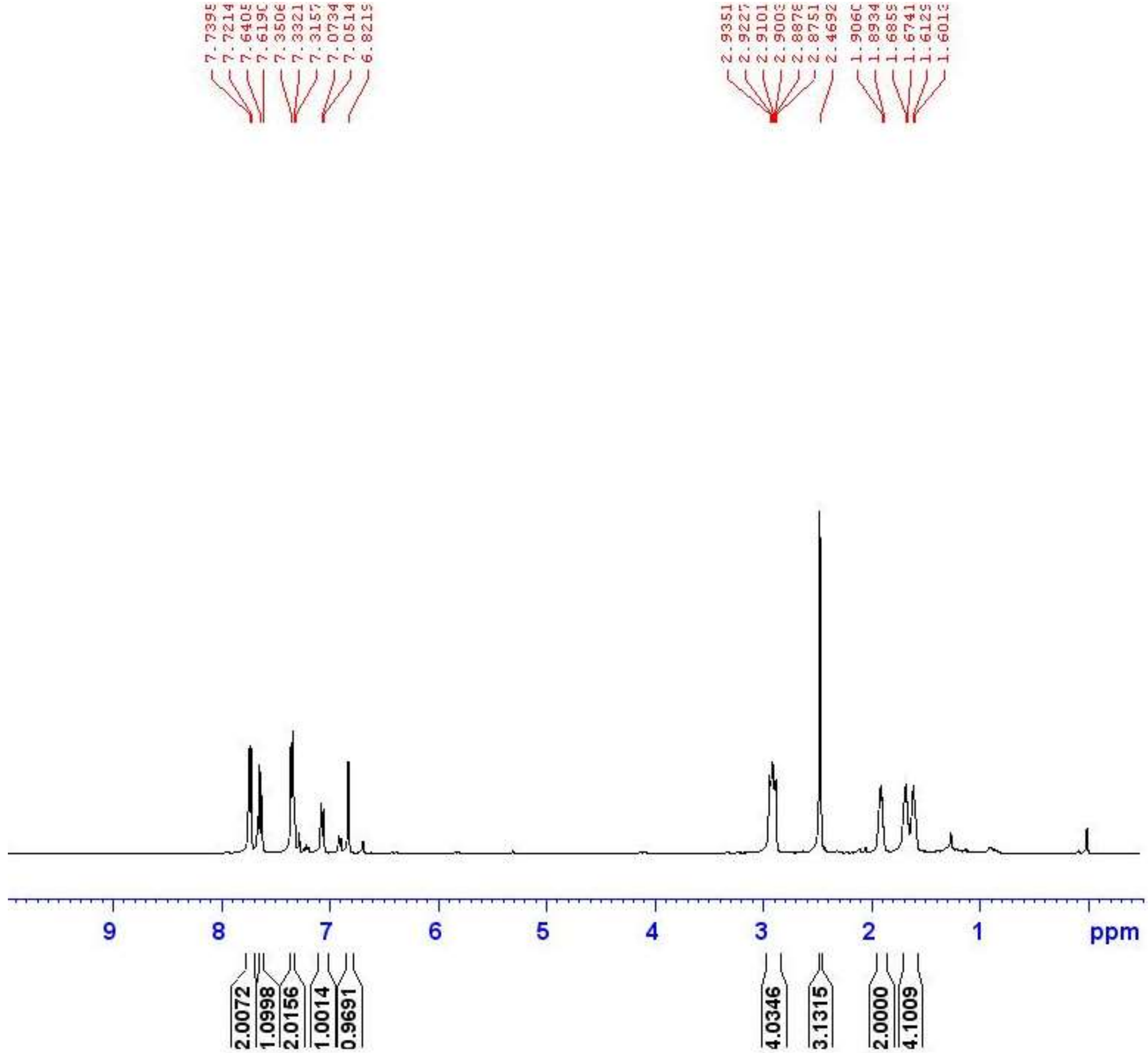
7.7395
7.7214
7.6405
7.6190
7.3506
7.3321
7.3157
7.0734
7.0514
6.8219
2.9351
2.9227
2.9101
2.9003
2.8878
2.8751
2.4692
1.9060
1.8934
1.6859
1.6741
1.6129
1.6013
9
8
7
6
5
4
3
2
1
ppm
2.0072
1.0998
2.0156
1.0014
0.9691
4.0346
3.1315
2.0000
4.1009

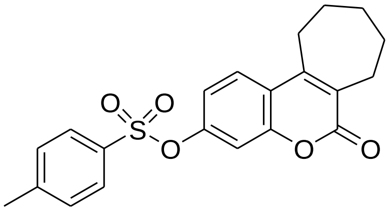
O
O
S
O
O
O
O

1f

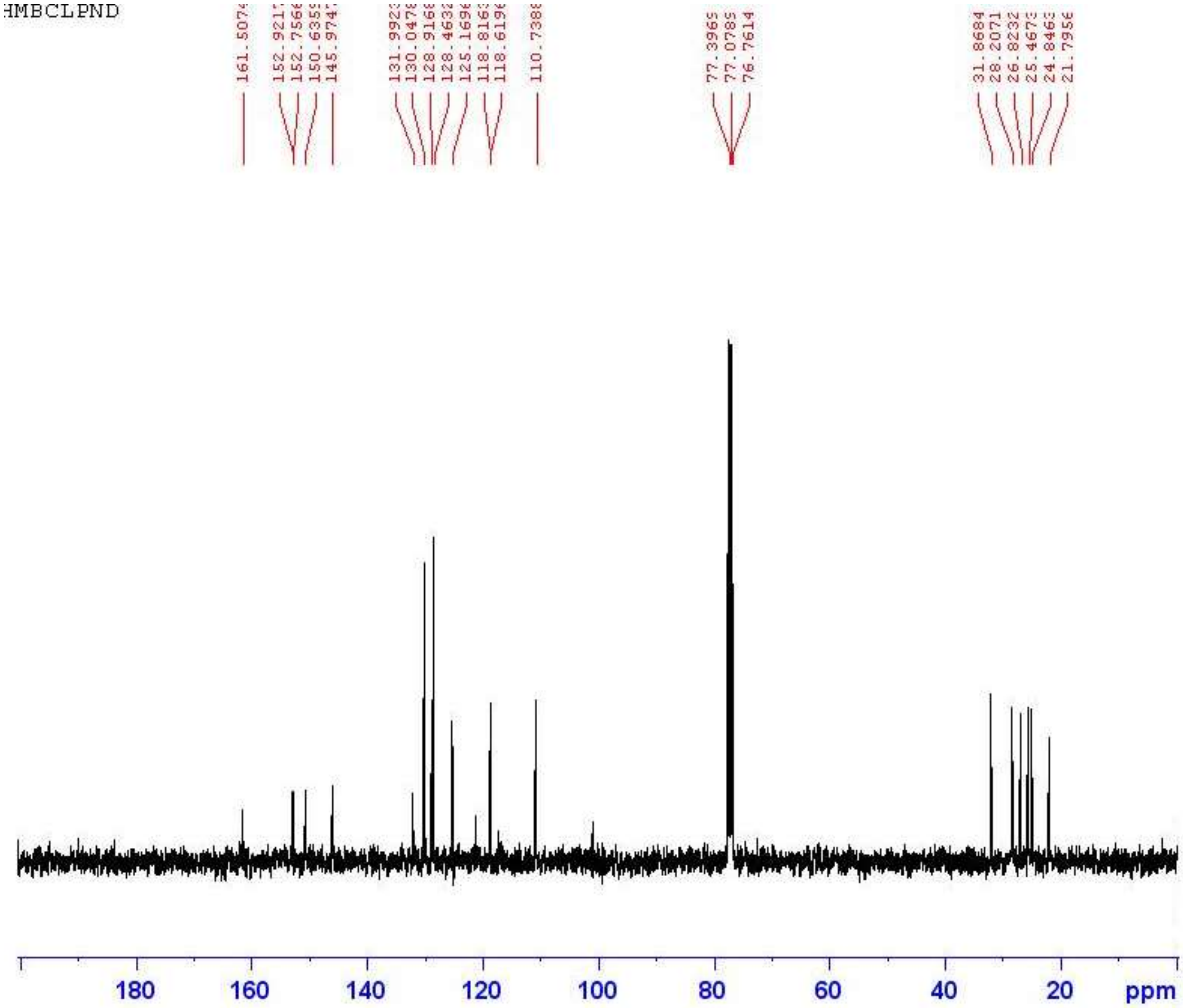
HMBCLPND
161.5074
152.9217
152.7566
150.6359
145.9747
131.9923
130.0478
128.9168
128.4632
125.1696
118.8163
118.6196
110.7388
77.3965
77.0789
76.7614
31.8684
28.2071
26.8232
25.4673
24.8463
21.7956
180
160
140
120
100
80
60
40
20
ppm

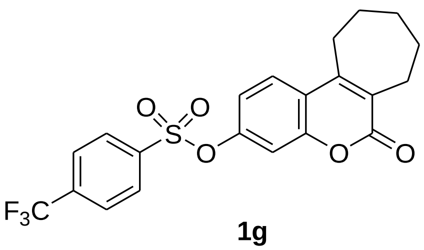
O
S
O
O
O
O
F3C

1g

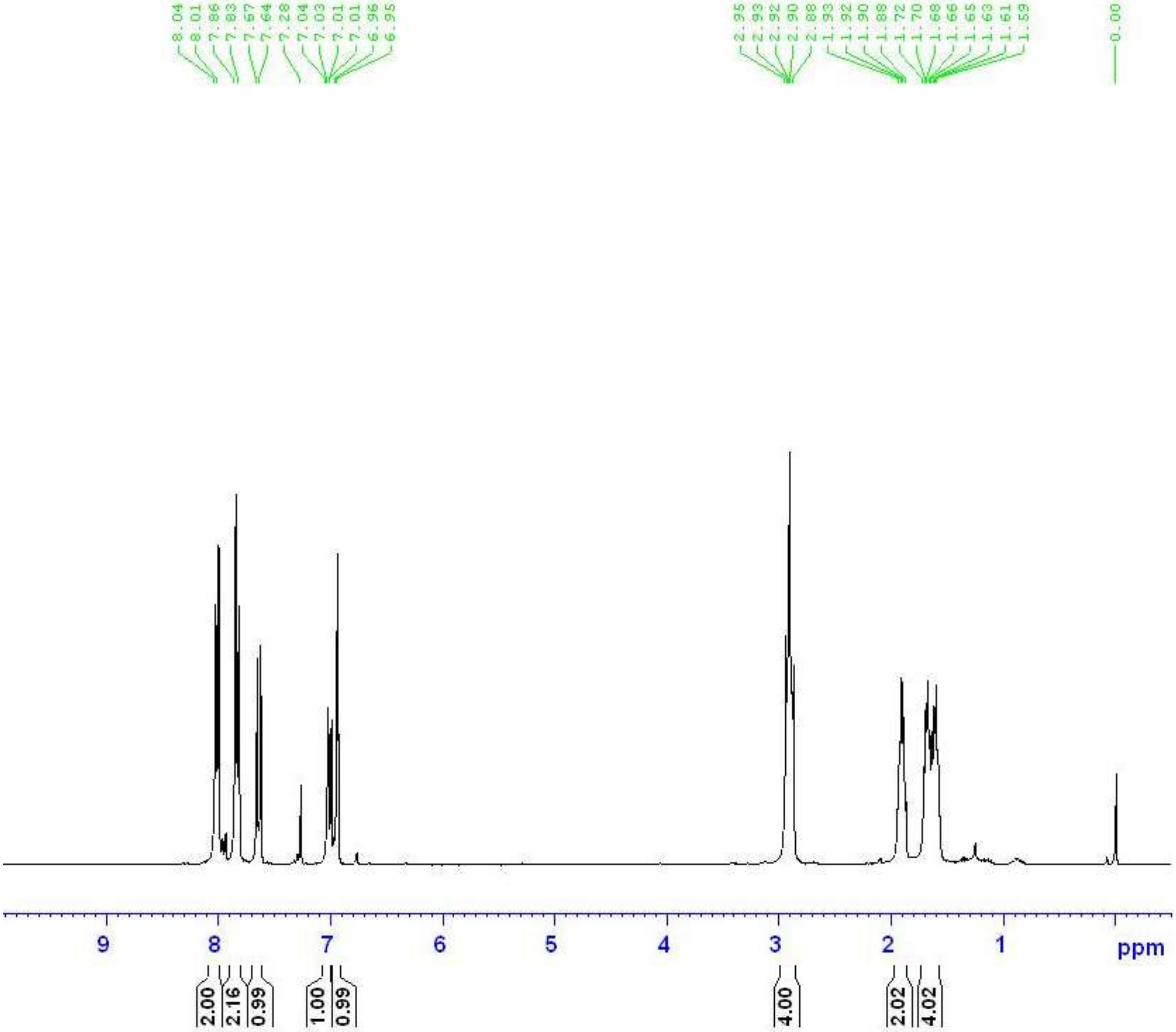
8.04
8.01
7.86
7.83
7.67
7.64
7.28
7.04
7.03
7.01
7.01
6.96
6.95
2.95
2.93
2.92
2.90
2.88
1.93
1.92
1.90
1.88
1.72
1.70
1.68
1.66
1.65
1.63
1.61
1.59
0.00
9
8
7
6
5
4
3
2
1
ppm
2.00
2.16
0.99
1.00
0.99
4.00
2.02
4.02

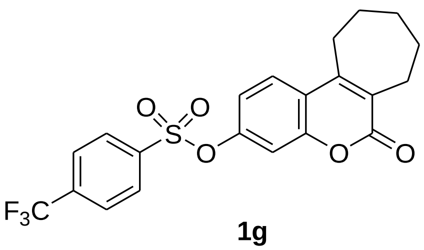

O
S
O
O
O
O
F_3C

1g

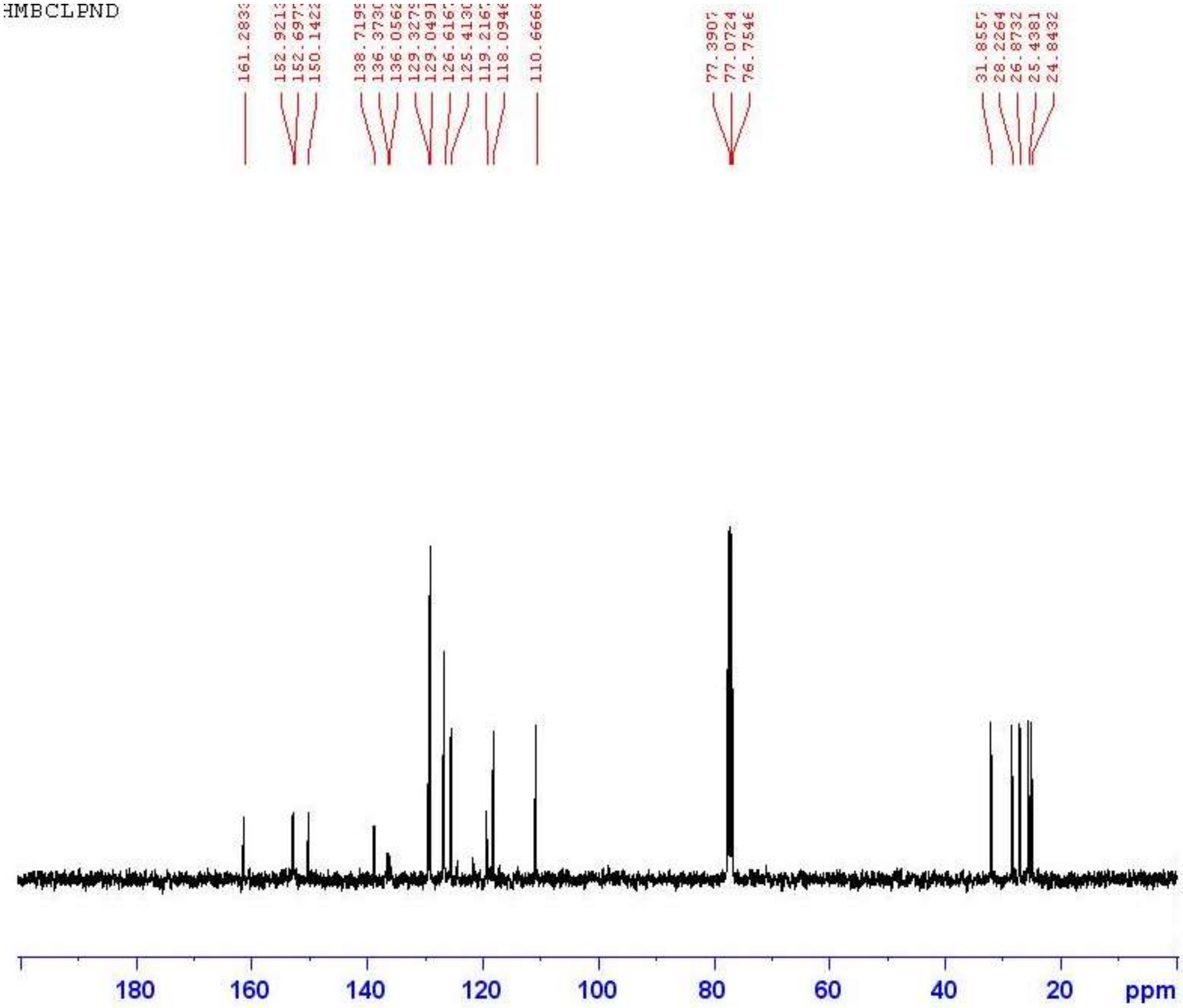

HMBCLPND
161.2833
152.9213
152.6977
150.1422
138.7199
136.3730
136.0562
129.3279
129.0491
126.6167
125.4130
119.2167
118.0946
110.6666
77.3907
77.0724
76.7546
31.8557
28.2264
26.8732
25.4381
24.8432
180
160
140
120
100
80
60
40
20
ppm

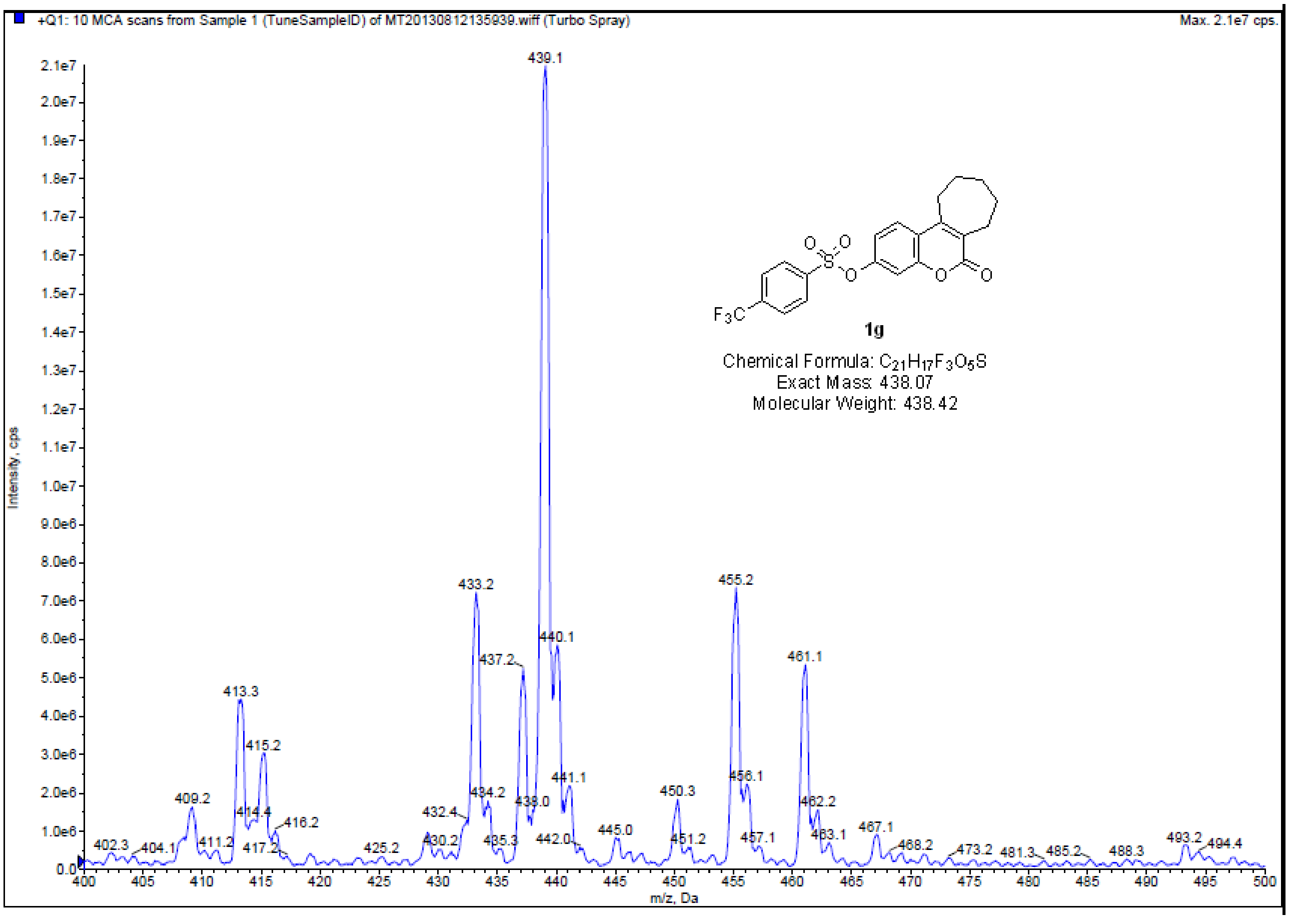

+Q1: 10 MCA scans from Sample 1 (TuneSampleID) of MT20130812135939.wiff (Turbo Spray)
Max. 2.1e7 cps.
Intensity, cps
m/z, Da
439.1
433.2
455.2
440.1
437.2
461.1
413.3
415.2
441.1
456.1
409.2
450.3
434.2
462.2
438.0
414.4
432.4
416.2
467.1
445.0
463.1
493.2
494.4
402.3
404.1
411.2
417.2
425.2
430.2
435.3
442.0
451.2
457.1
468.2
473.2
481.3
485.2
488.3
1g
Chemical Formula: $C_{21}H_{17}F_3O_5S$
Exact Mass: 438.07
Molecular Weight: 438.42

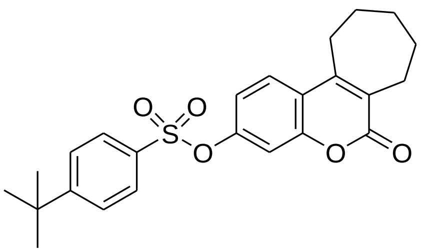

O
O
S
O
O
O

1h

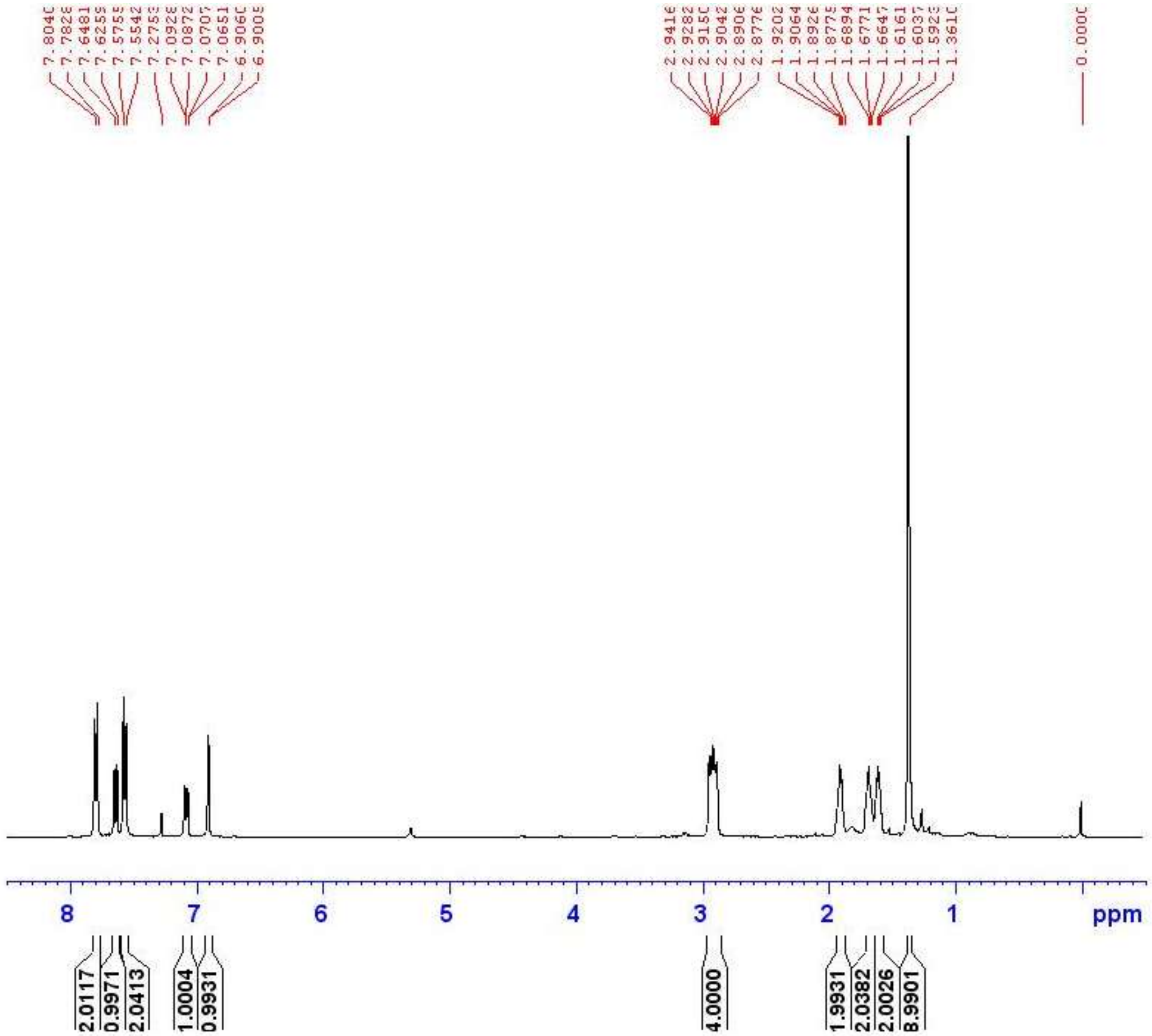

7.8040
7.7828
7.6481
7.6259
7.5755
7.5542
7.2753
7.0928
7.0872
7.0707
7.0651
6.9060
6.9005
2.9416
2.9282
2.9150
2.9042
2.8906
2.8776
1.9202
1.9064
1.8926
1.8779
1.6894
1.6771
1.6647
1.6161
1.6037
1.5929
1.3610
0.0000
8
7
6
5
4
3
2
1
ppm
2.0117
0.9971
2.0413
1.0004
0.9931
4.0000
1.9931
2.0382
2.0026
8.9901

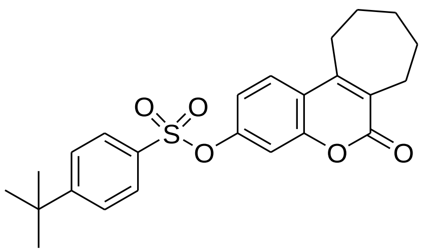
O
O
S
O
O
O

1h

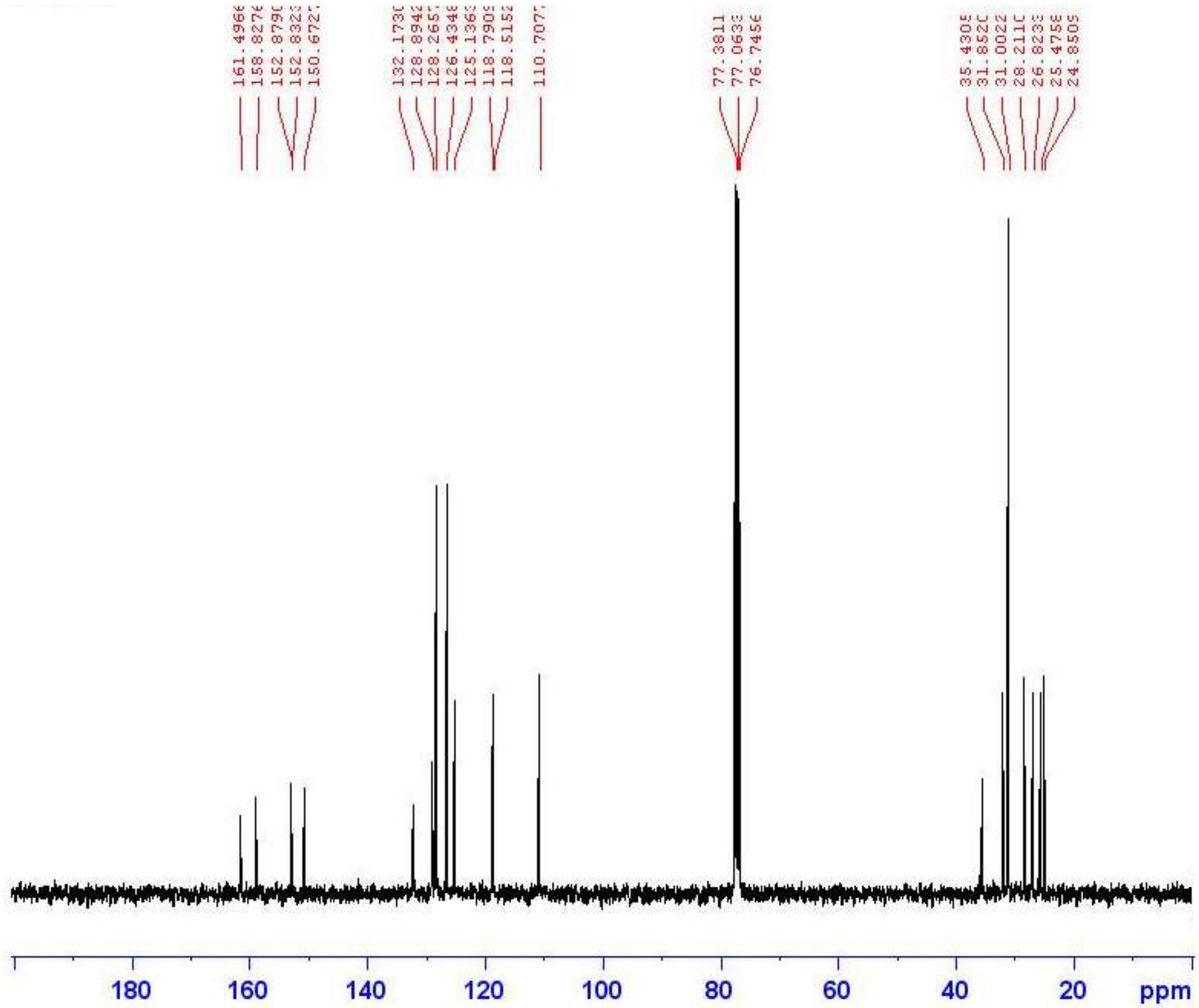
161.4966
158.8276
152.8790
152.8323
150.6727
132.1730
128.8942
128.2657
126.4348
125.1369
118.7909
118.5152
110.7077
77.3811
77.0633
76.7456
35.4305
31.8520
31.0022
28.2110
26.8233
25.4758
24.8505
180
160
140
120
100
80
60
40
20
ppm

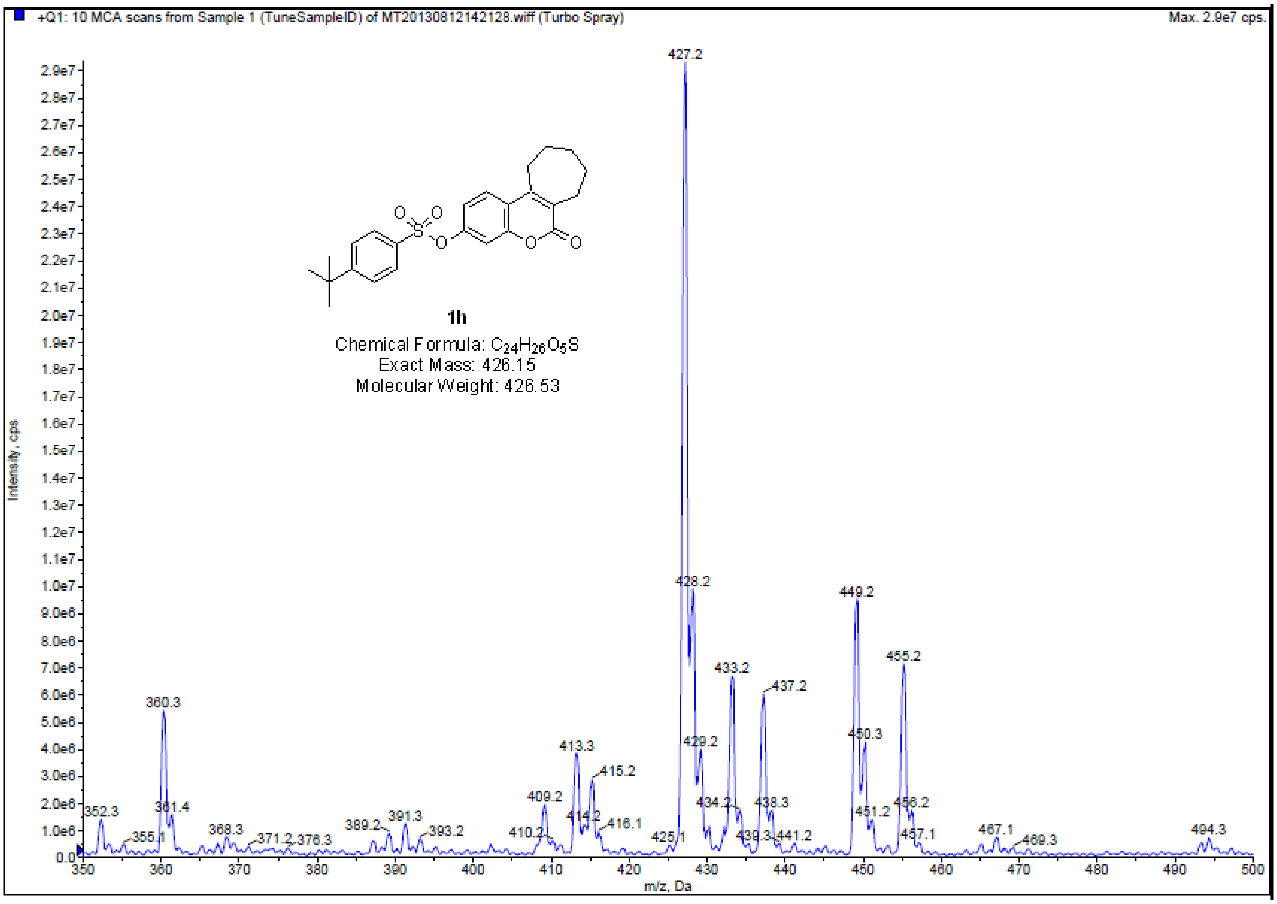

+Q1: 10 MCA scans from Sample 1 (TuneSampleID) of MT20130812142128.wiff (Turbo Spray)
Max. 2.9e7 cps.
1h
Chemical Formula: $C_{24}H_{26}O_5S$
Exact Mass: 426.15
Molecular Weight: 426.53
Intensity, cps
m/z, Da
427.2
428.2
449.2
455.2
433.2
437.2
360.3
413.3
415.2
429.2
450.3
409.2

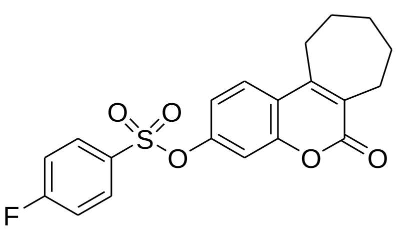
O
O
S
O
O
O
O
F

1i

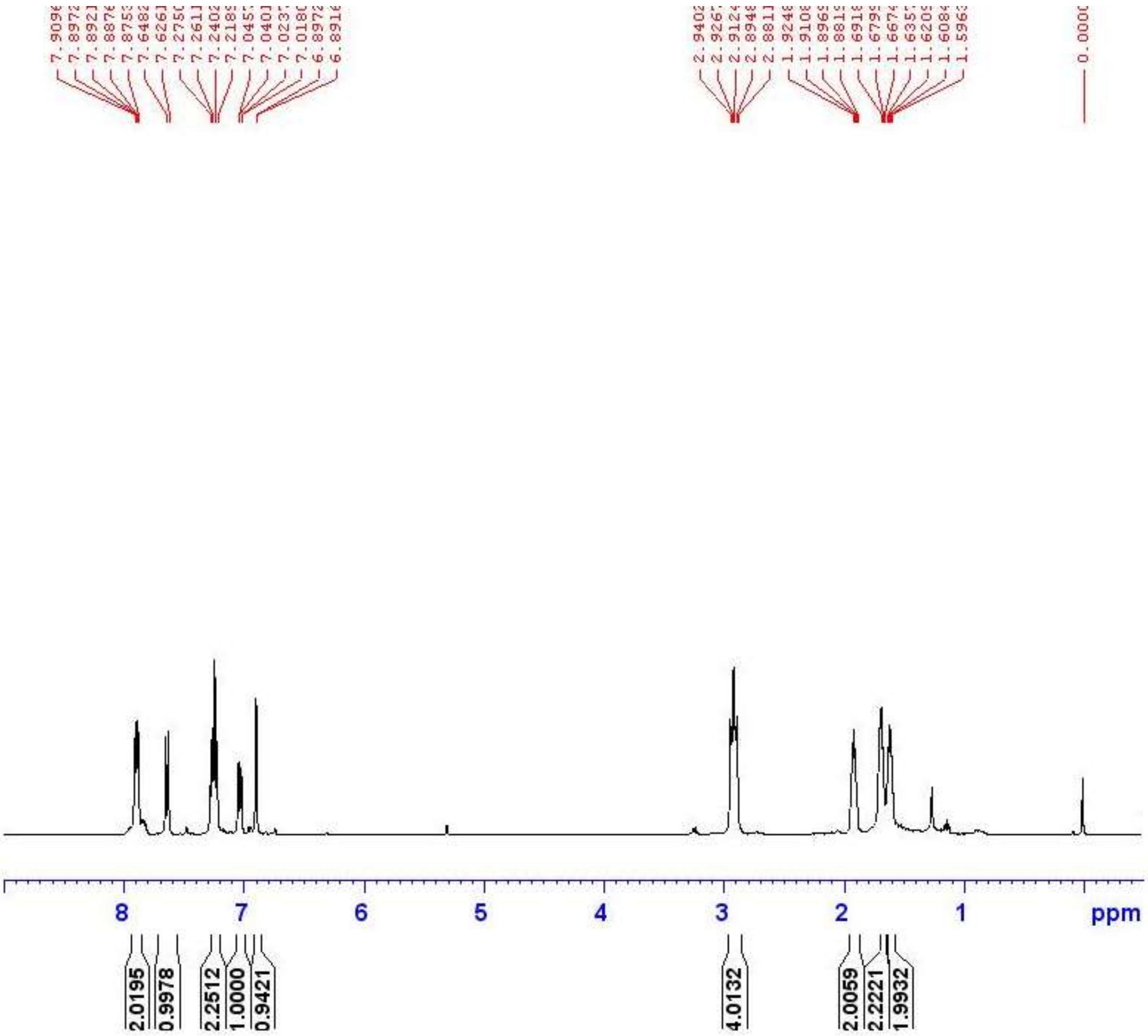
8
7
6
5
4
3
2
1
ppm
2.0195
0.9978
2.2512
1.0000
0.9421
4.0132
2.0059
2.2221
1.9932

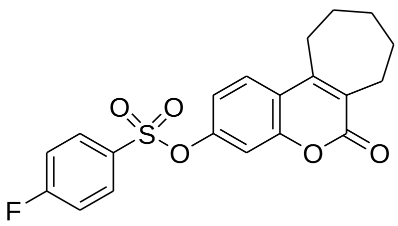

O
O
S
O
O
O
F

1i

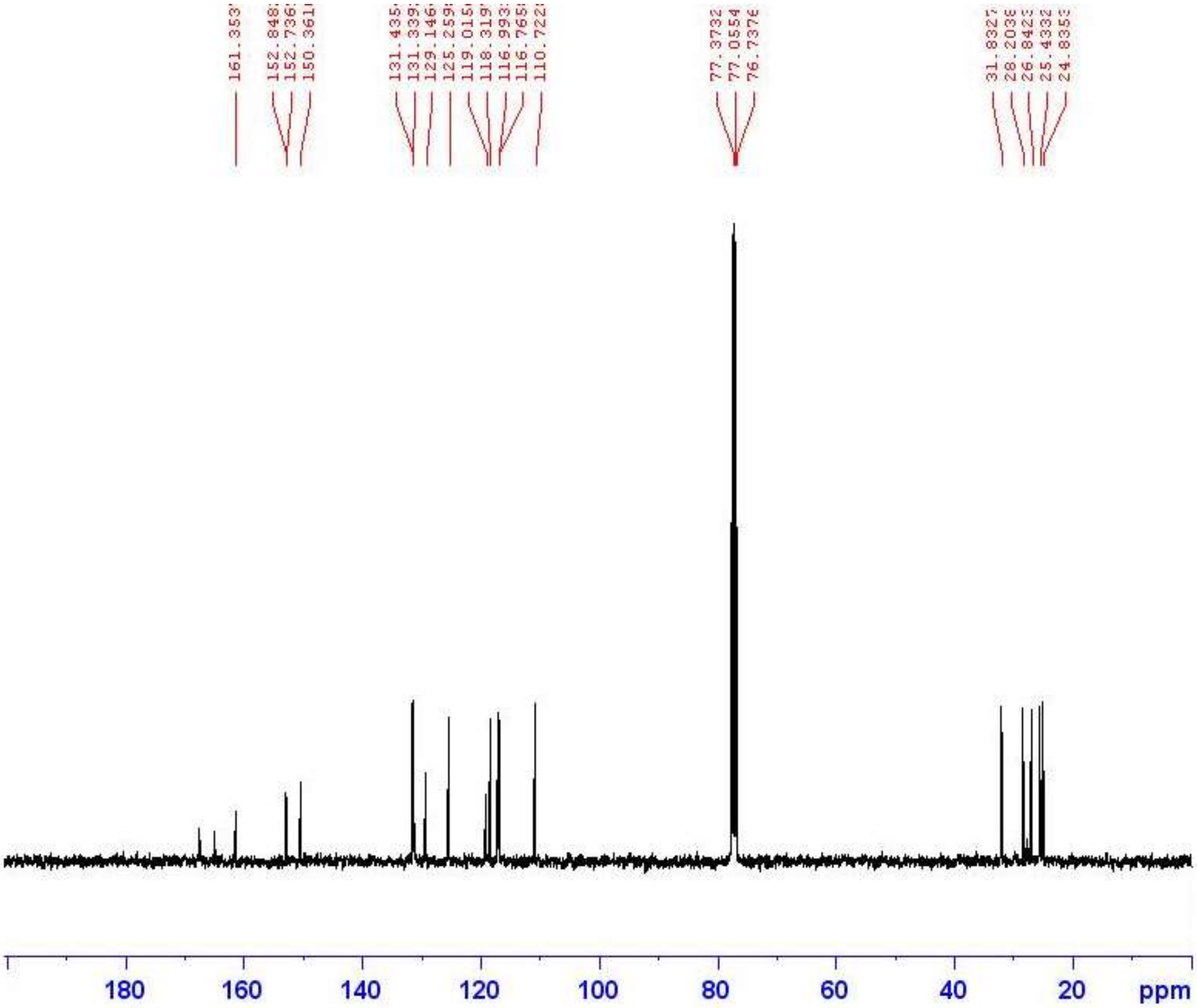

161.353
152.848
152.736
150.361
131.435
131.339
129.146
125.259
119.015
118.319
116.993
116.765
110.722
77.3732
77.0554
76.7376
31.8327
28.2038
26.8423
25.4332
24.8353
180
160
140
120
100
80
60
40
20
ppm

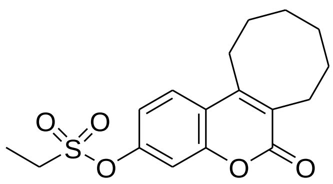

1j

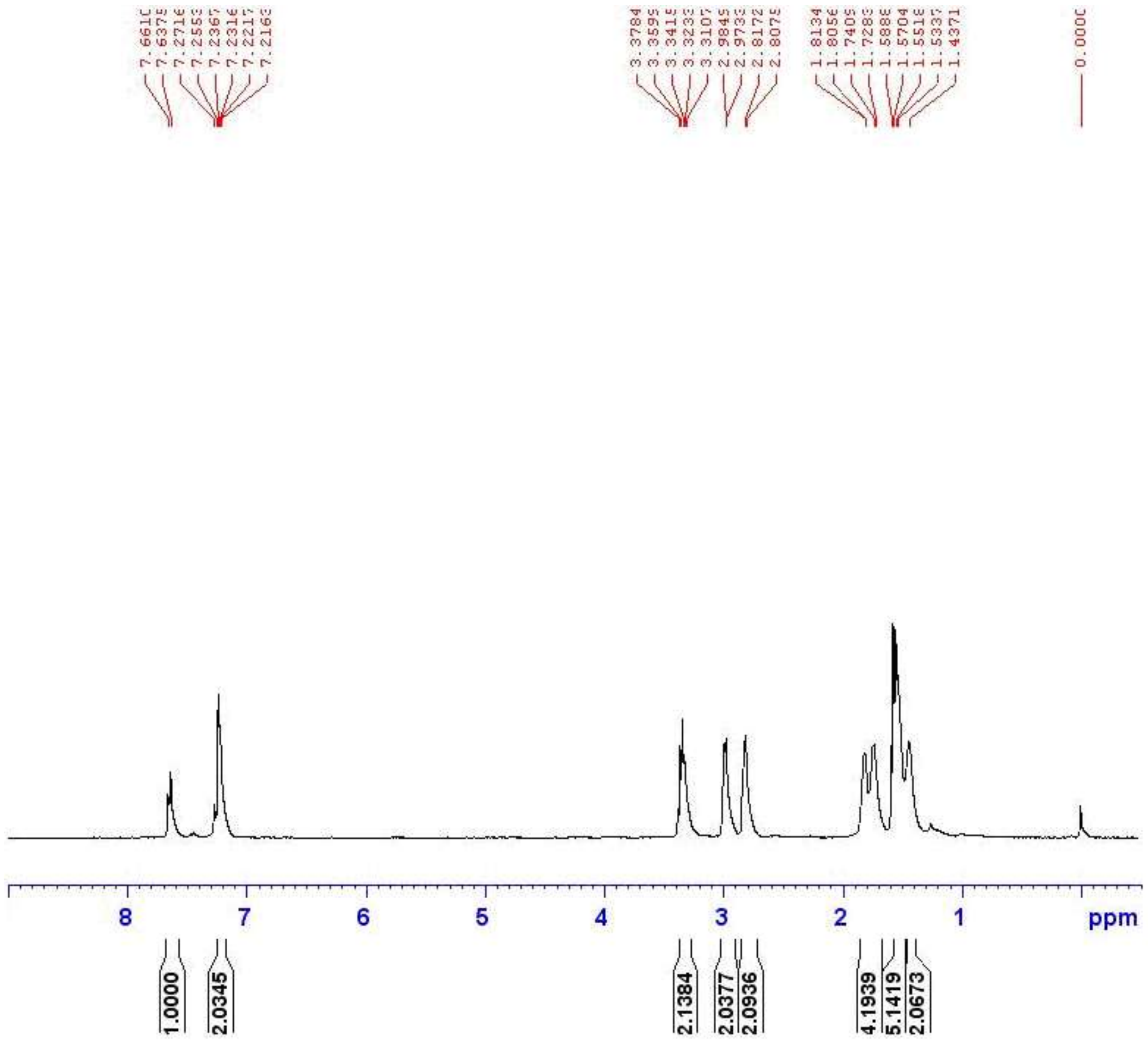

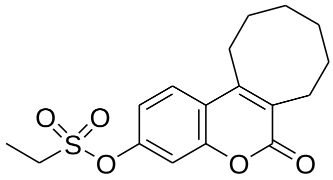
O
O
S
O
O
O

1j

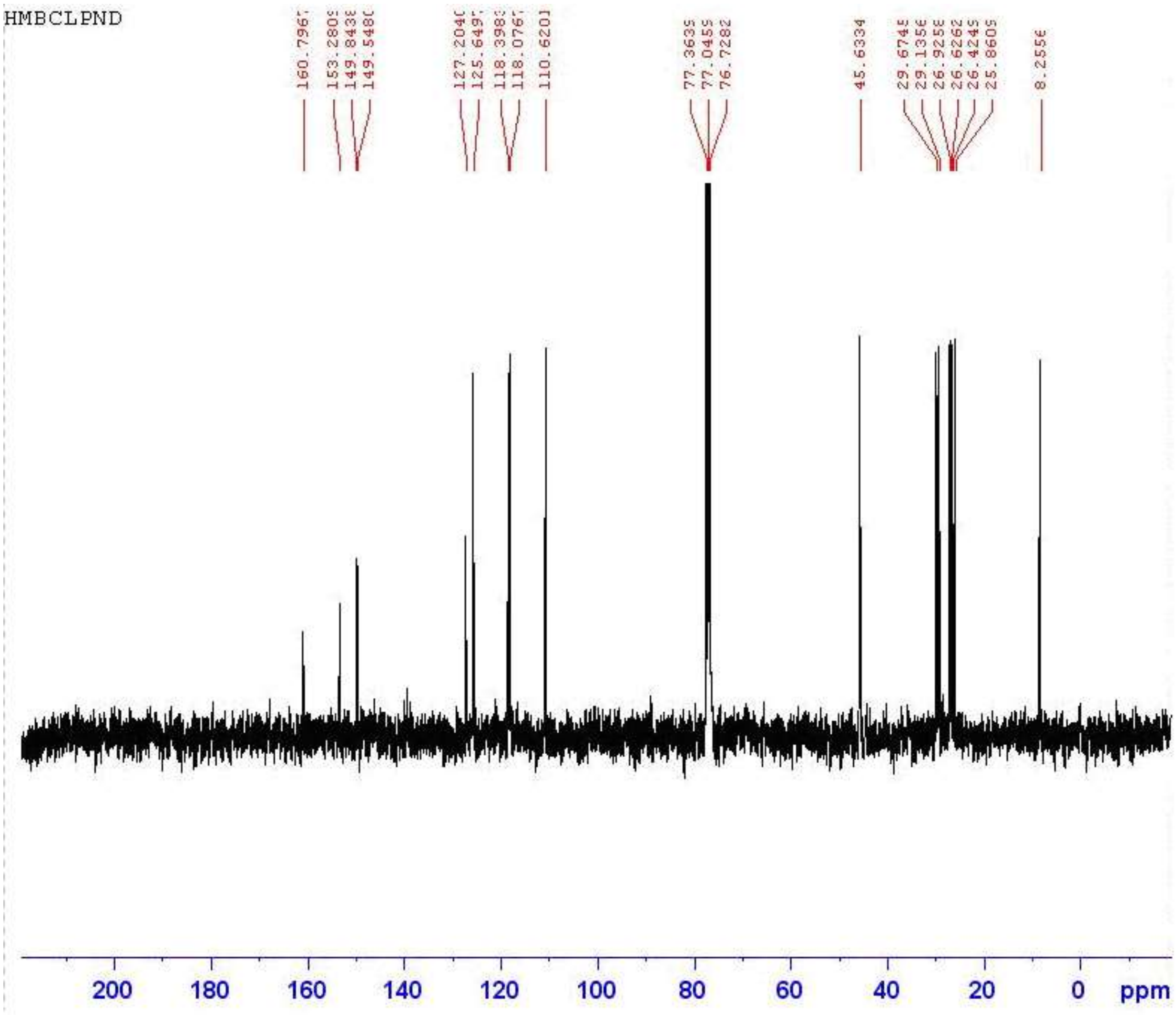
HMBCLPND
160.796
153.280
149.843
149.548
127.204
125.649
118.398
118.076
110.620
77.363
77.045
76.7282
45.6334
29.674
29.135
26.925
26.6262
26.424
25.860
8.255
200
180
160
140
120
100
80
60
40
20
0
ppm

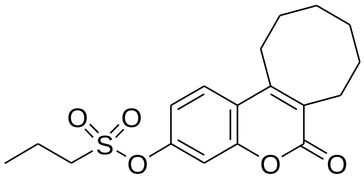

1k

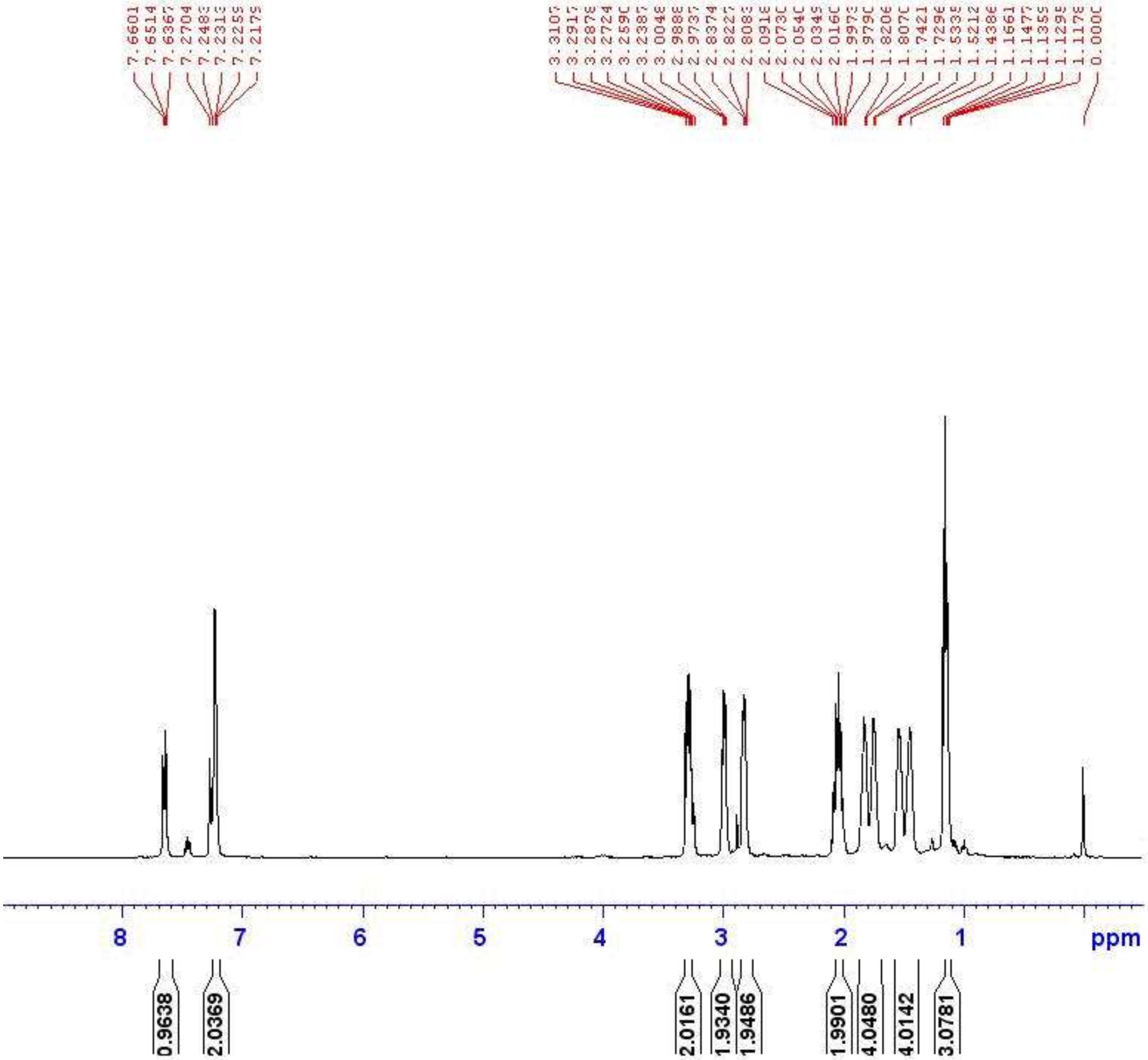
8
7
6
5
4
3
2
1
ppm
0.9638
2.0369
2.0161
1.9340
1.9486
1.9901
4.0480
4.0142
3.0781

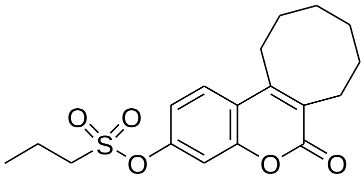

1k

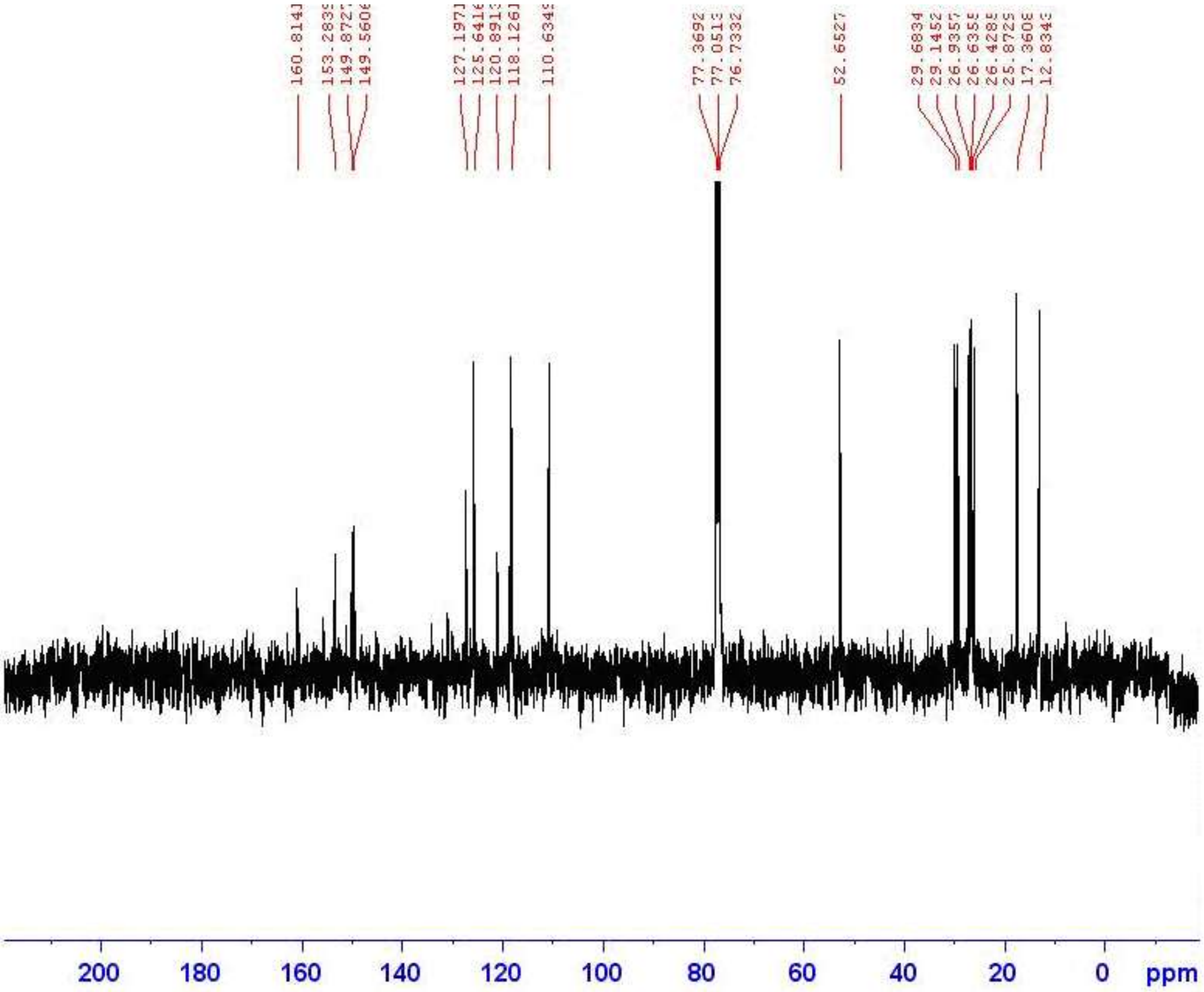

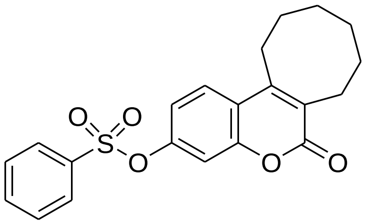

1l

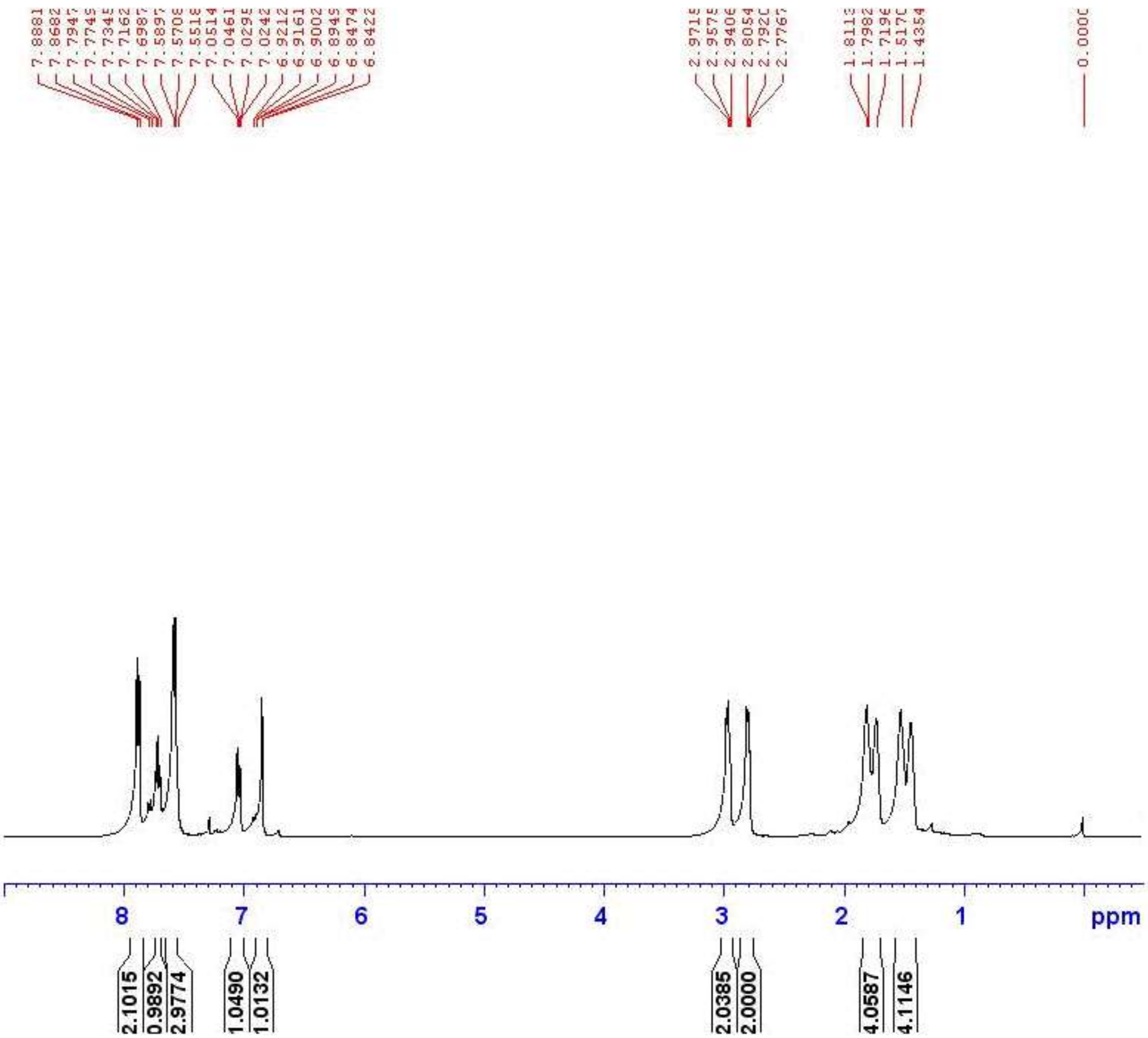
7.8881
7.8682
7.7947
7.7745
7.7345
7.7162
7.6987
7.5897
7.5708
7.5518
7.0514
7.0461
7.0295
7.0242
6.9212
6.9161
6.9002
6.8949
6.8474
6.8422
2.9715
2.9575
2.9406
2.8054
2.7920
2.7767
1.8113
1.7982
1.7196
1.5170
1.4354
0.0000
8
7
6
5
4
3
2
1
ppm
2.1015
0.9892
2.9774
1.0490
1.0132
2.0385
2.0000
4.0587
4.1146

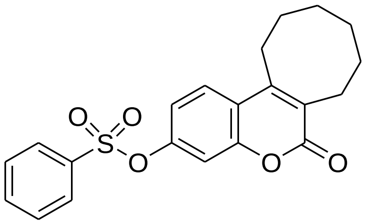

O
S
O
O
O
O

1l

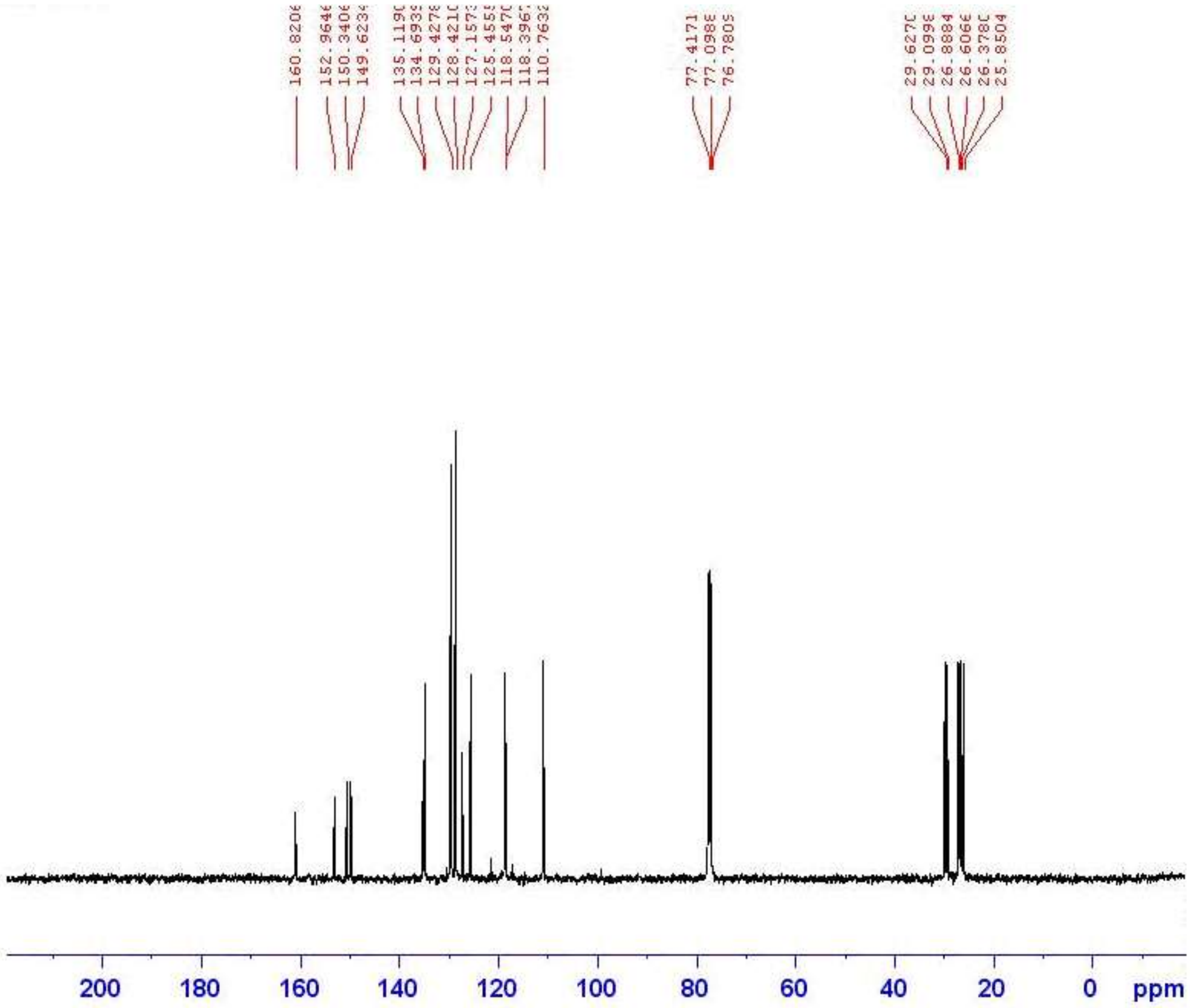

160.8206
152.9646
150.3406
149.6234
135.1190
134.6939
129.4278
128.4210
127.1573
125.4555
118.5470
118.3967
110.7632
77.4171
77.0988
76.7809
29.6270
29.0998
26.8884
26.6066
26.3780
25.8504
200
180
160
140
120
100
80
60
40
20
0
ppm

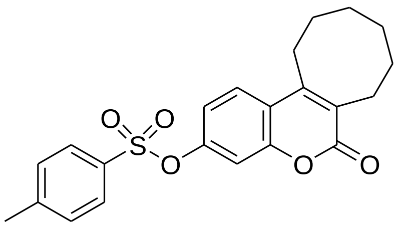
O
O
S
O
O
O

1m

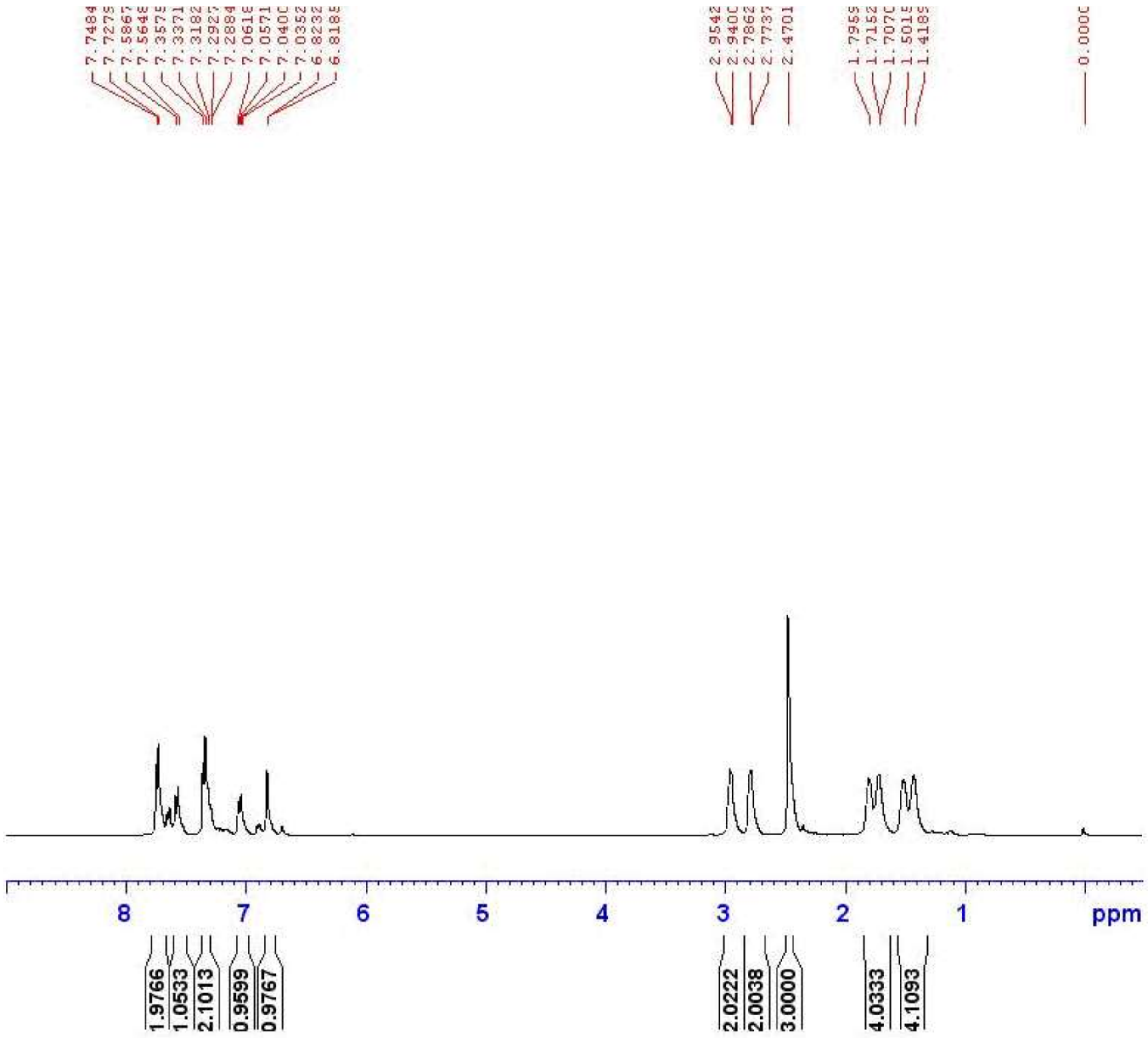
7.7484
7.7275
7.5867
7.5648
7.3575
7.3371
7.3182
7.2927
7.2884
7.0616
7.0571
7.0400
7.0352
6.8232
6.8185
2.9542
2.9400
2.7862
2.7737
2.4701
1.7959
1.7152
1.7070
1.5015
1.4189
0.0000
8
7
6
5
4
3
2
1
ppm
1.9766
1.0533
2.1013
0.9599
0.9767
2.0222
2.0038
3.0000
4.0333
4.1093

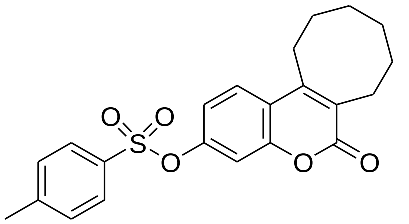
O
S
O
O
O
O

1m

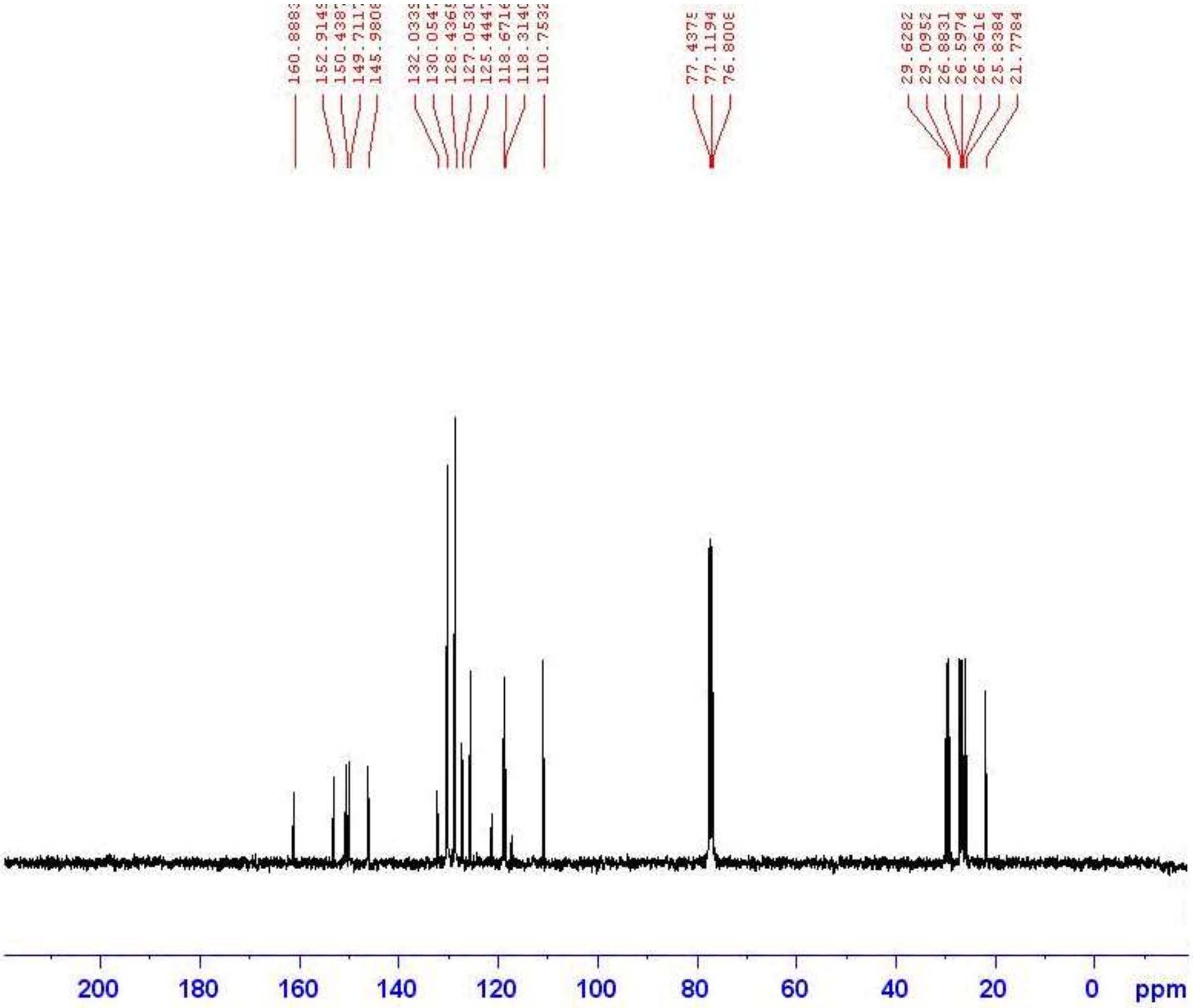
160.8883
152.9149
150.4387
149.7117
145.9808
132.0339
130.0547
128.4365
127.0530
125.4447
118.6716
118.3140
110.7532
77.4375
77.1194
76.8008
29.6282
29.0952
26.8831
26.5974
26.3616
25.8384
21.7784
200
180
160
140
120
100
80
60
40
20
0
ppm

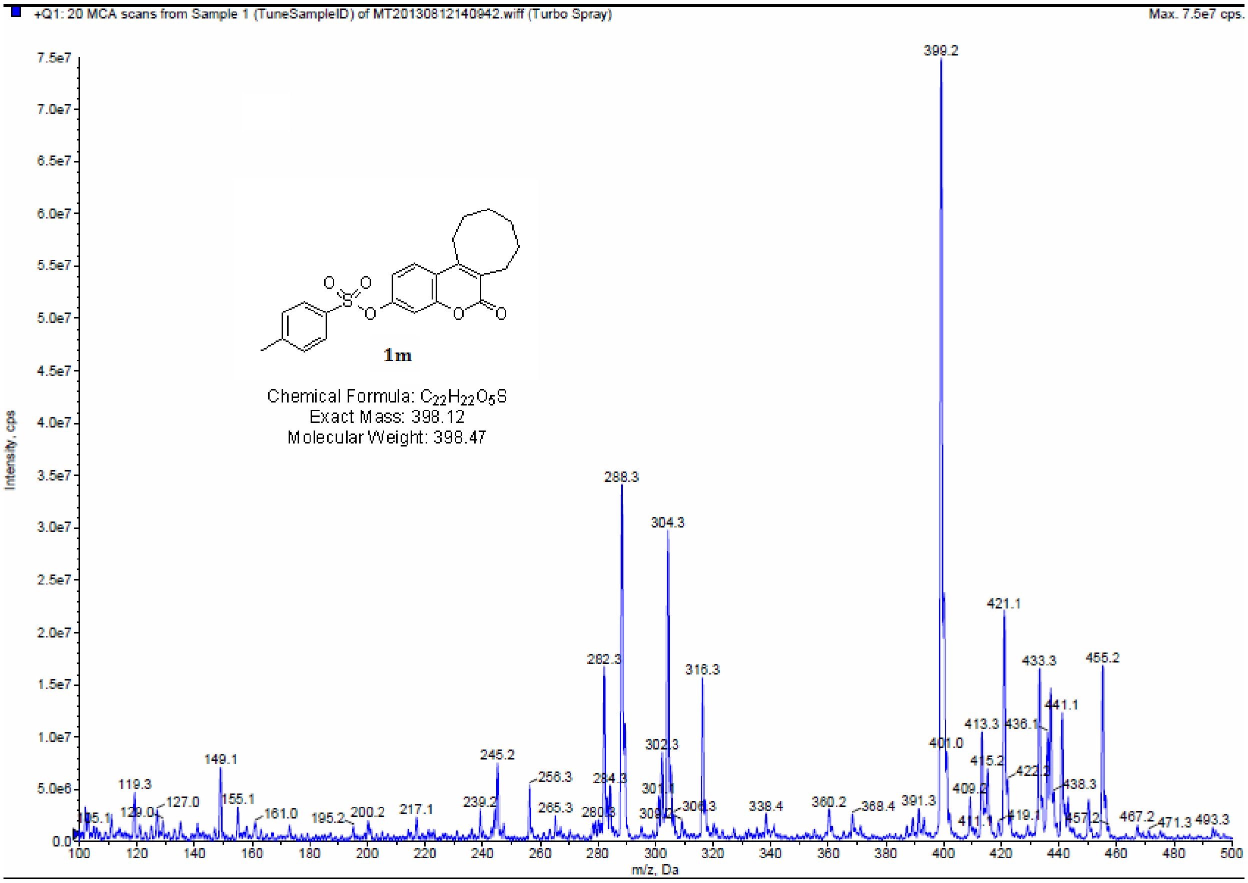
+Q1: 20 MCA scans from Sample 1 (TuneSampleID) of MT20130812140942.wiff (Turbo Spray)
Max. 7.5e7 cps.
1m
Chemical Formula: C22H22O5S
Exact Mass: 398.12
Molecular Weight: 398.47
Intensity, cps
m/z, Da
399.2
288.3
304.3
421.1
282.3
316.3
433.3
455.2
441.1
245.2
149.1
119.3

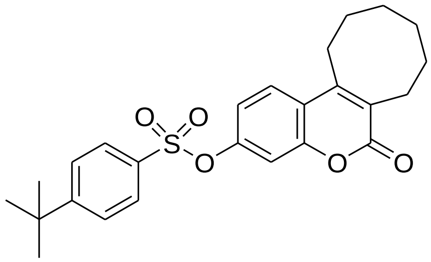

1n

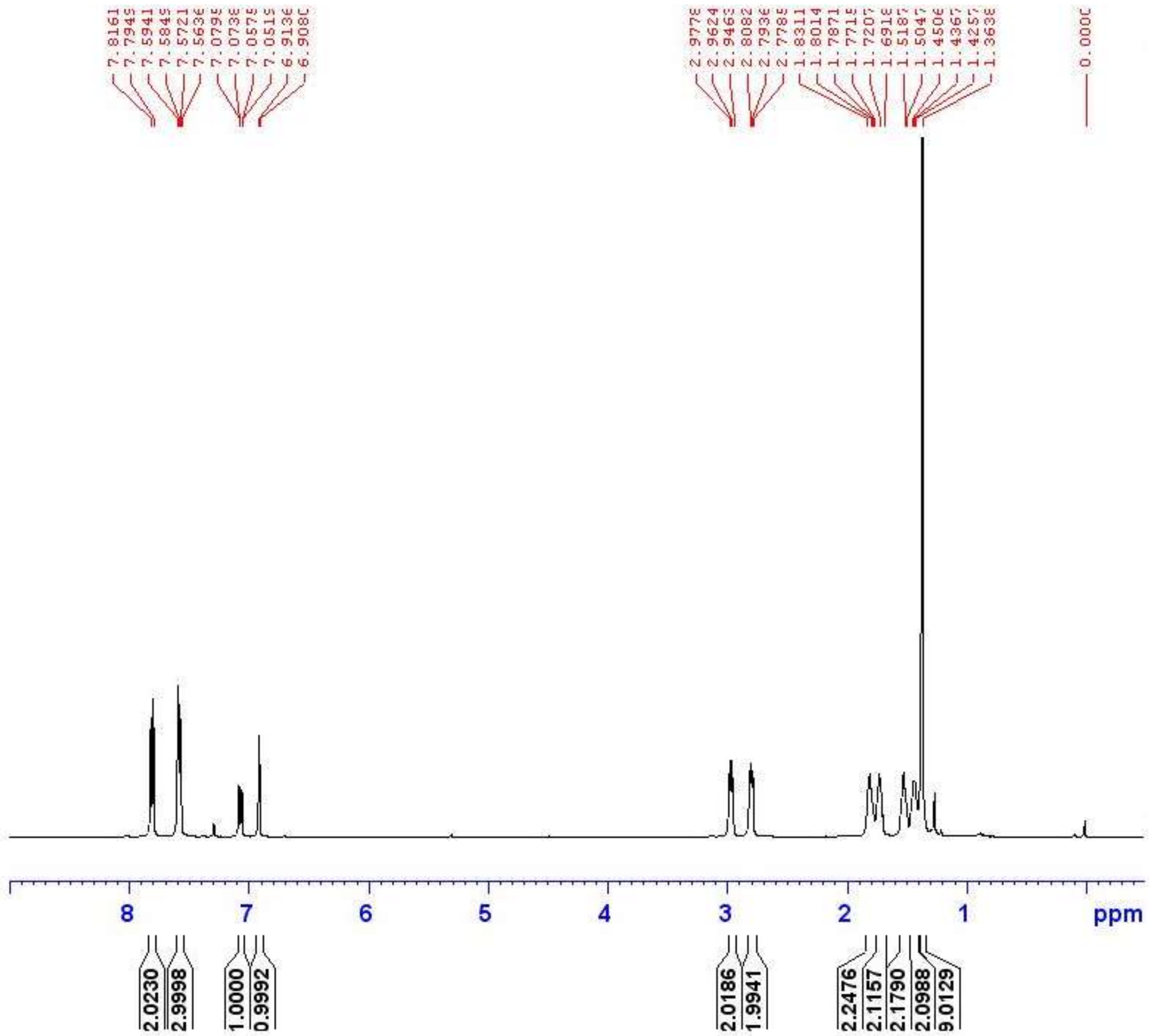

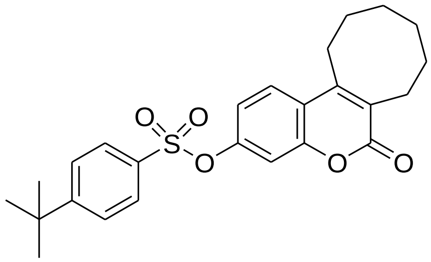

1n

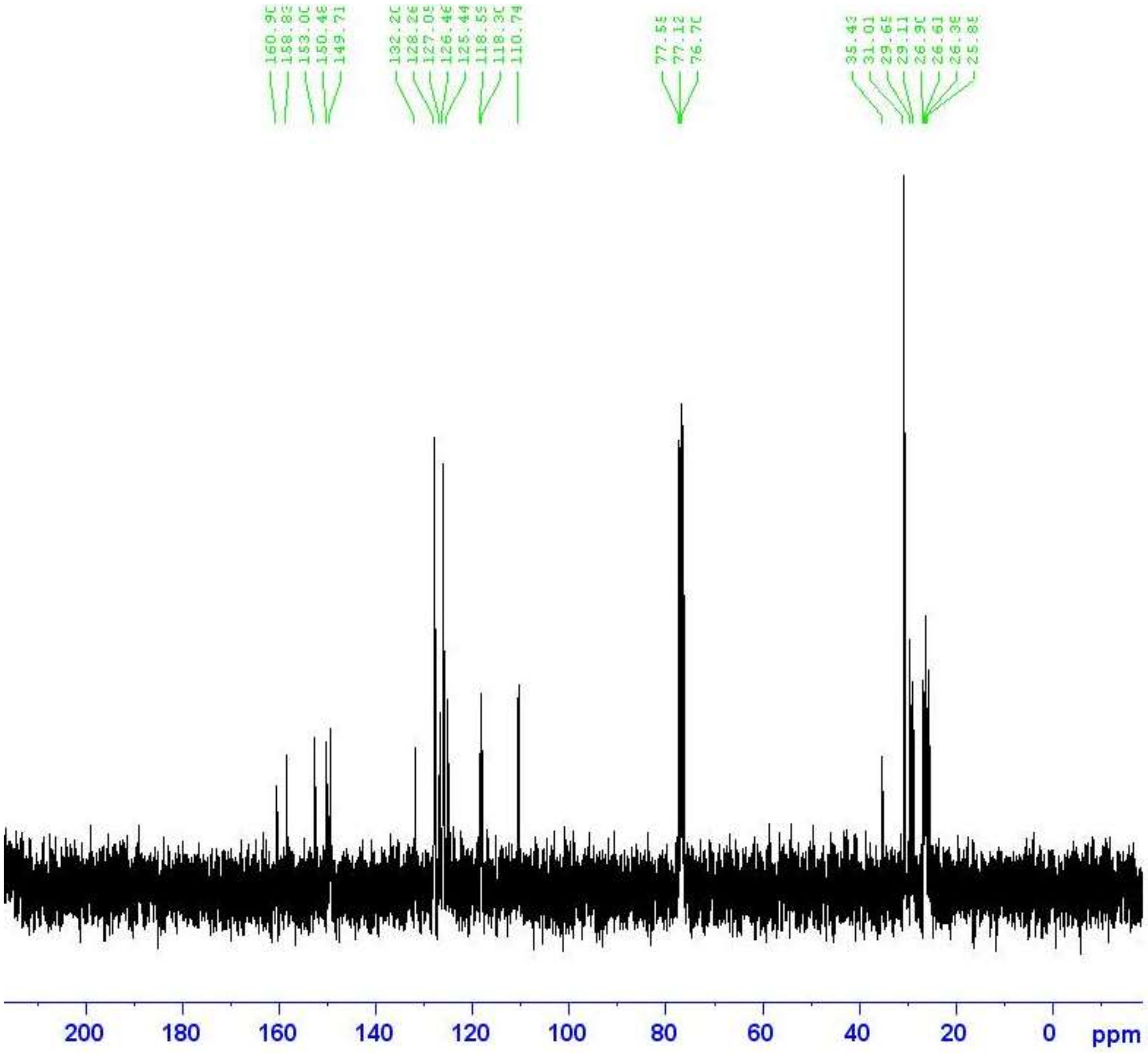

160.9C
158.83
153.0C
150.48
149.71
132.2C
128.26
127.09
126.46
125.44
118.59
118.30
110.74
77.58
77.12
76.7C
35.43
31.01
29.65
29.11
26.9C
26.61
26.38
25.88
200
180
160
140
120
100
80
60
40
20
0
ppm

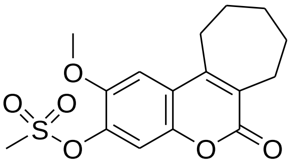

1o

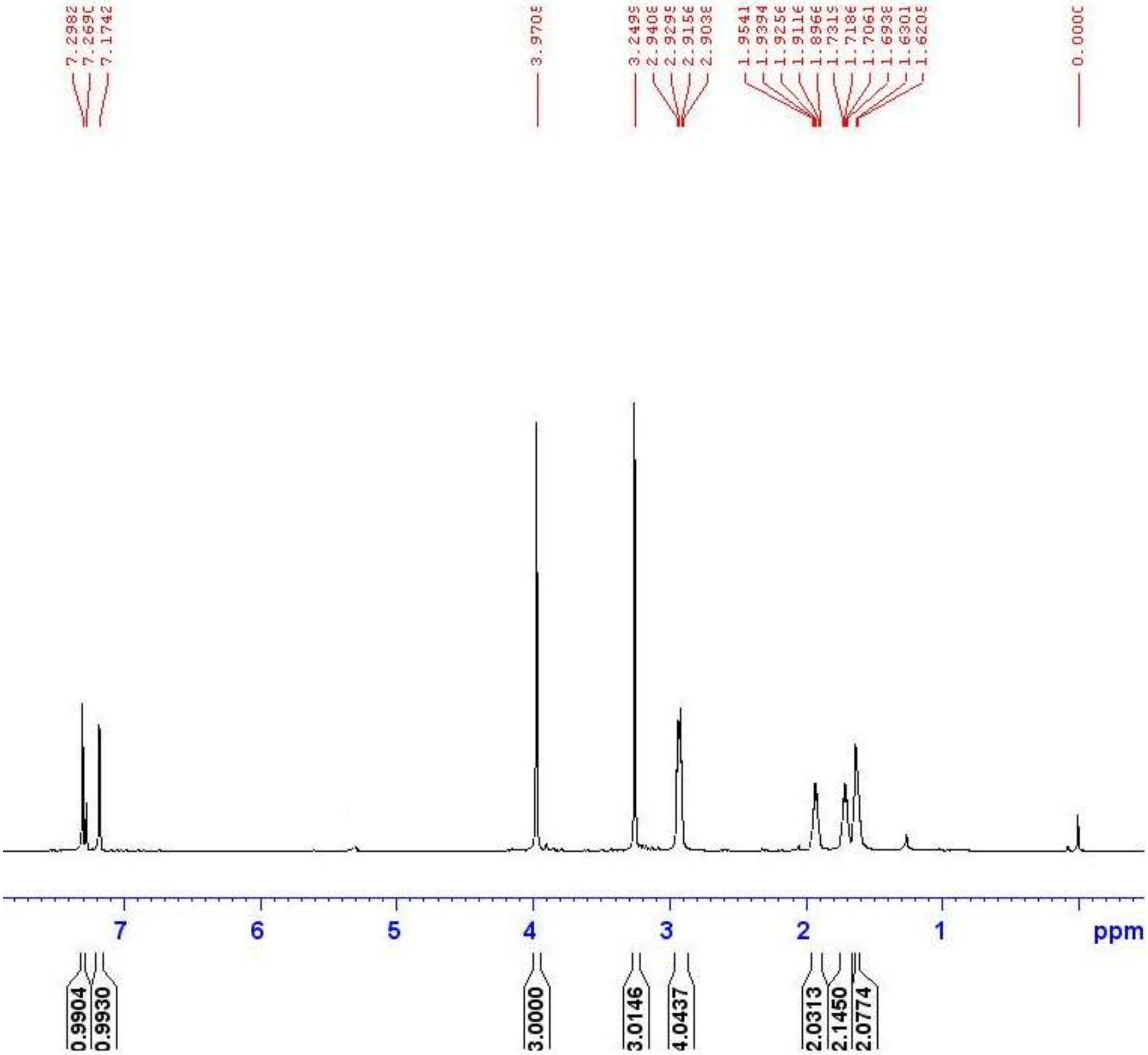

7
6
5
4
3
2
1
ppm
0.9904
0.9930
3.0000
3.0146
4.0437
2.0313
2.1450
2.0774

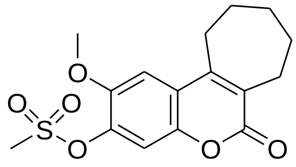
O
O
O
S
O
O
O
O

1o

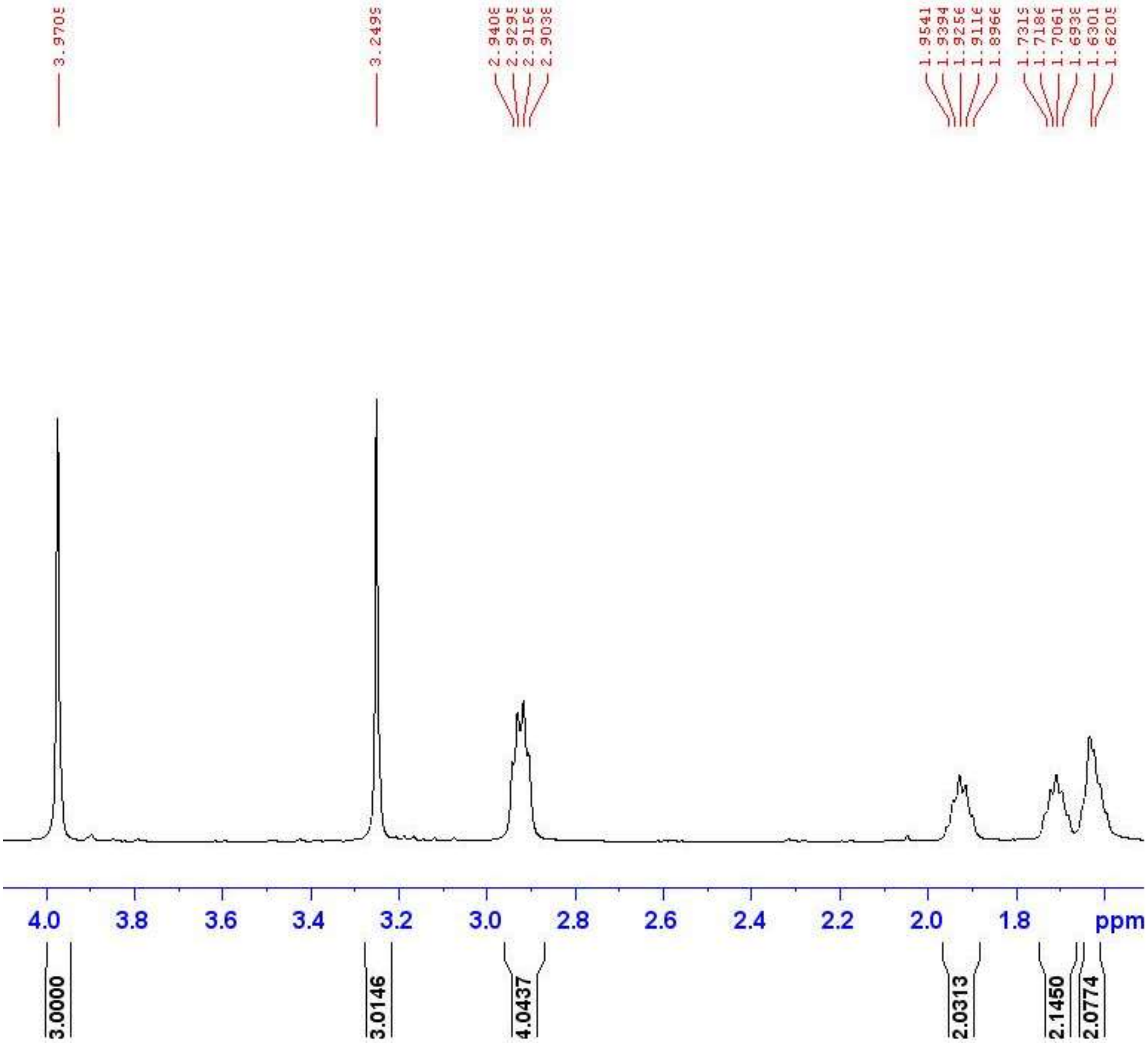
3.9705
3.2499
2.9408
2.9295
2.9156
2.9038
1.9541
1.9394
1.9256
1.9116
1.8966
1.7319
1.7186
1.7061
1.6938
1.6301
1.6205
4.0
3.8
3.6
3.4
3.2
3.0
2.8
2.6
2.4
2.2
2.0
1.8
ppm
3.0000
3.0146
4.0437
2.0313
2.1450
2.0774

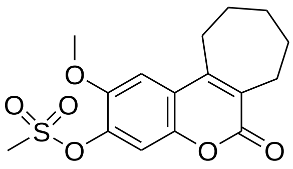

1o

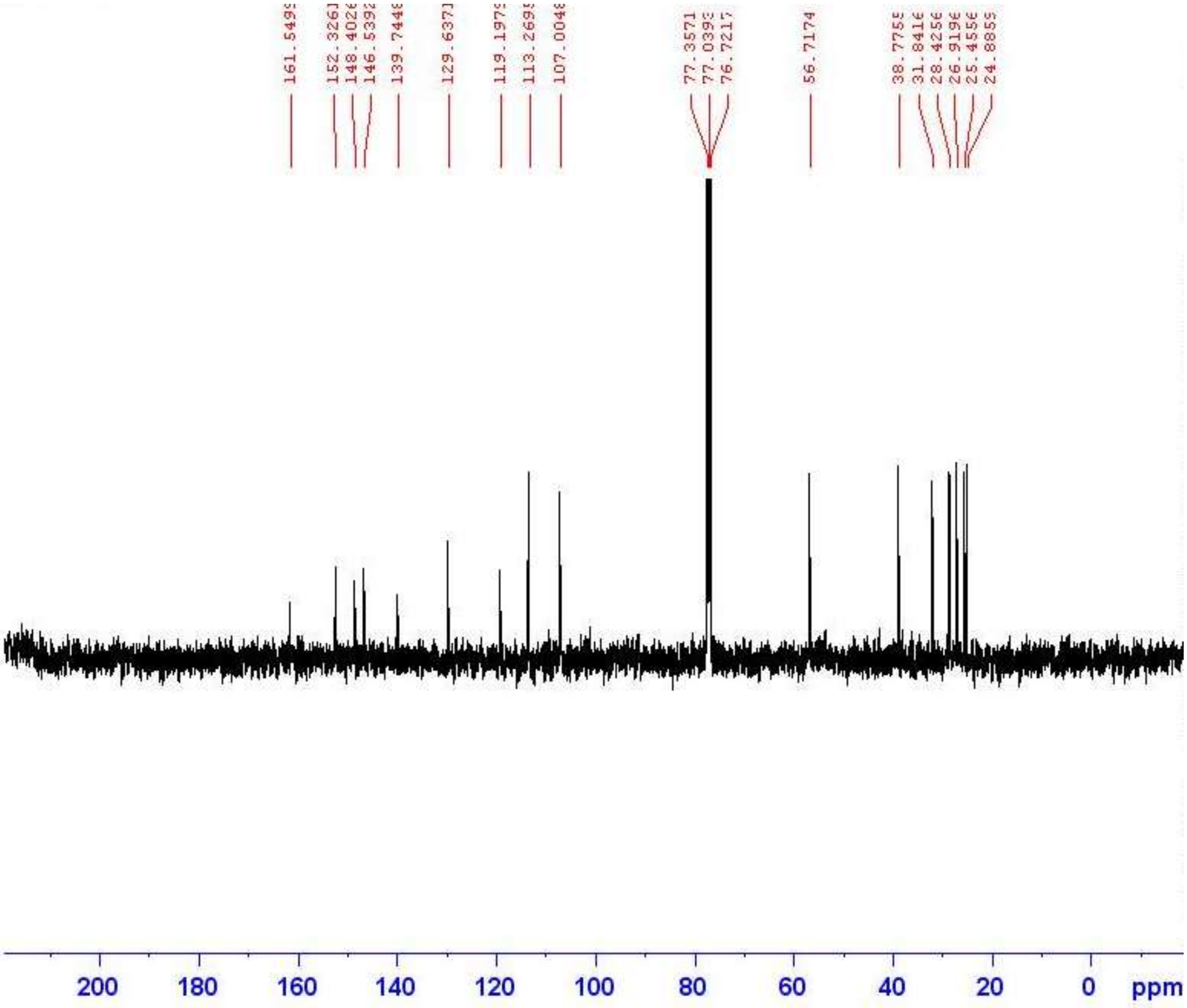
161.549
152.326
148.402
146.539
139.744
129.637
119.197
113.269
107.004
77.3571
77.039
76.721
56.7174
38.775
31.841
28.425
26.919
25.455
24.885
200
180
160
140
120
100
80
60
40
20
0
ppm

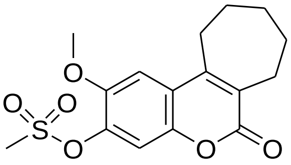

1o

Chemical Formula: $C_{16}H_{18}O_6S$
Exact Mass: 338.08
Molecular Weight: 338.38

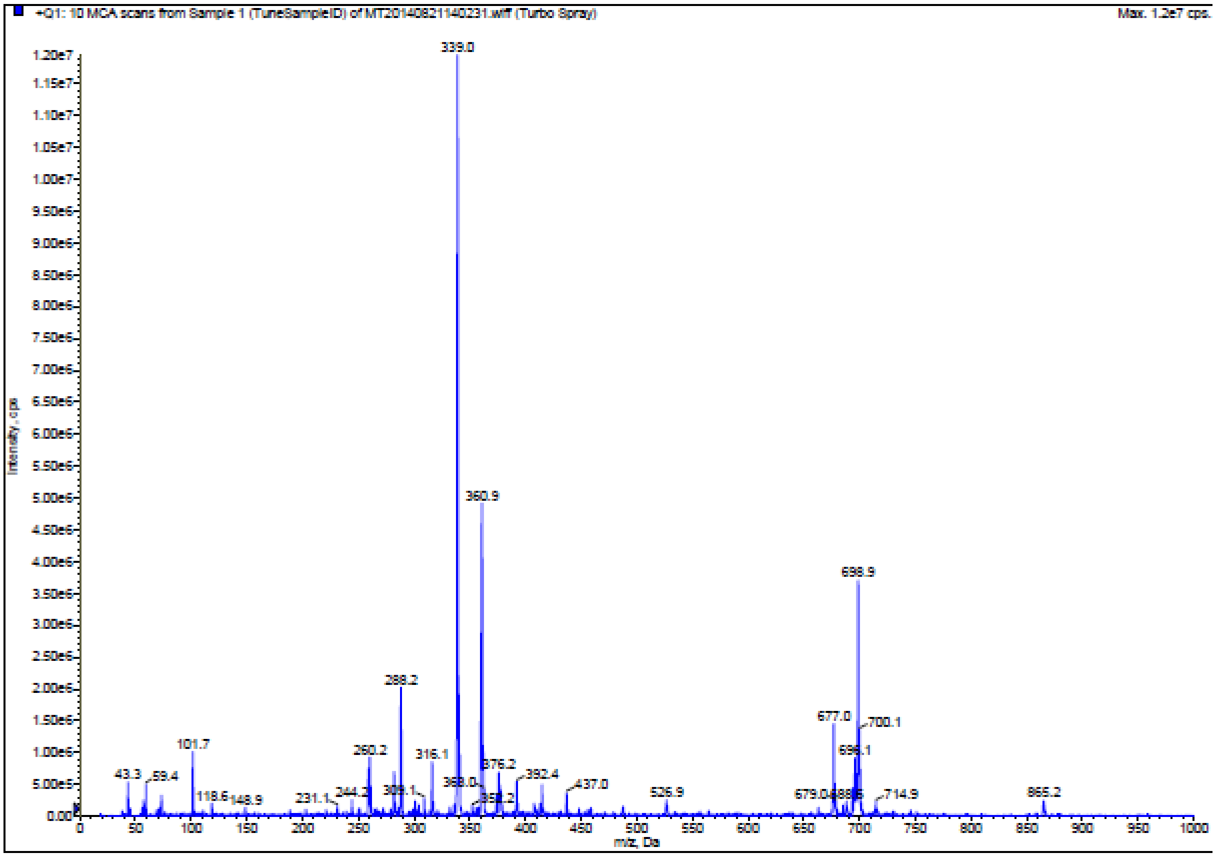

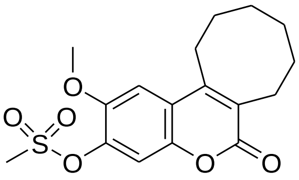

1p

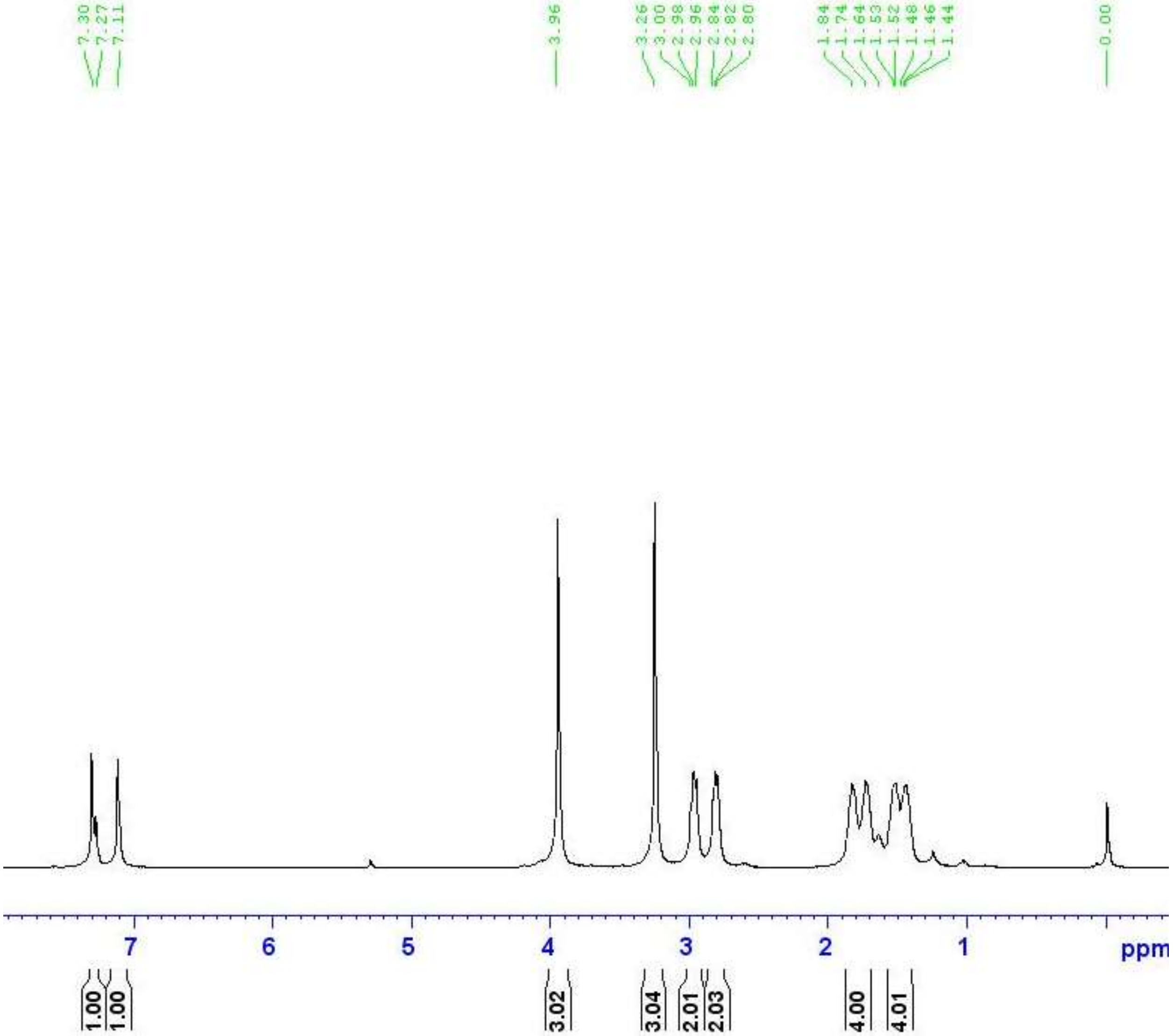
7.30
7.27
7.11
3.96
3.26
3.00
2.98
2.96
2.84
2.82
2.80
1.84
1.74
1.64
1.53
1.52
1.48
1.46
1.44
0.00
7
6
5
4
3
2
1
ppm
1.00
1.00
3.02
3.04
2.01
2.03
4.00
4.01

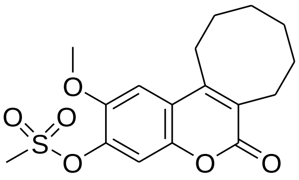

1p

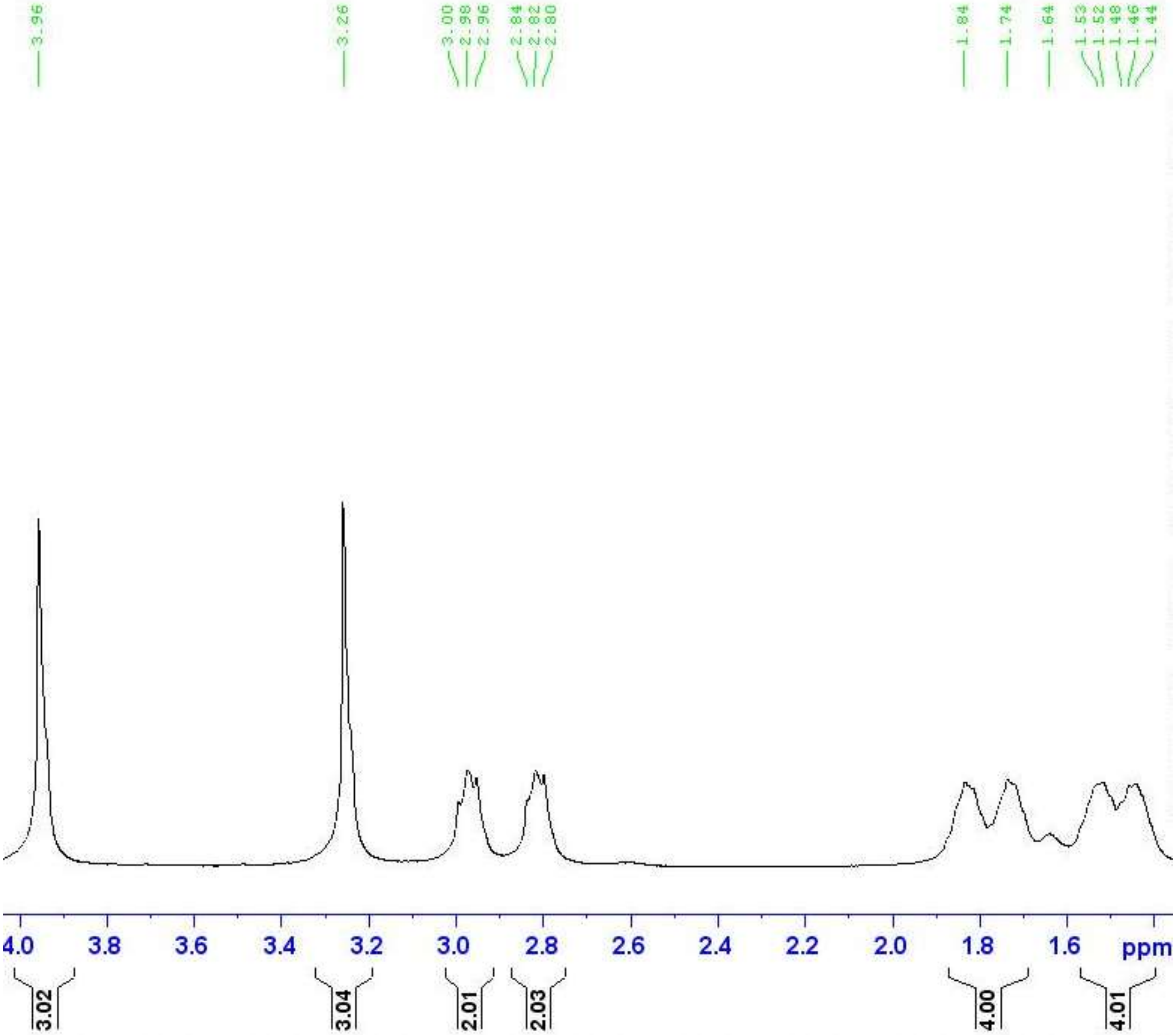

3.96
3.26
3.00
2.98
2.96
2.84
2.82
2.80
1.84
1.74
1.64
1.53
1.52
1.48
1.46
1.44
4.0
3.8
3.6
3.4
3.2
3.0
2.8
2.6
2.4
2.2
2.0
1.8
1.6
ppm
3.02
3.04
2.01
2.03
4.00
4.01

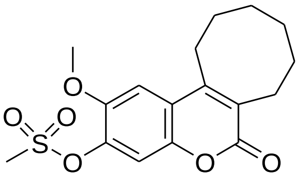

1p

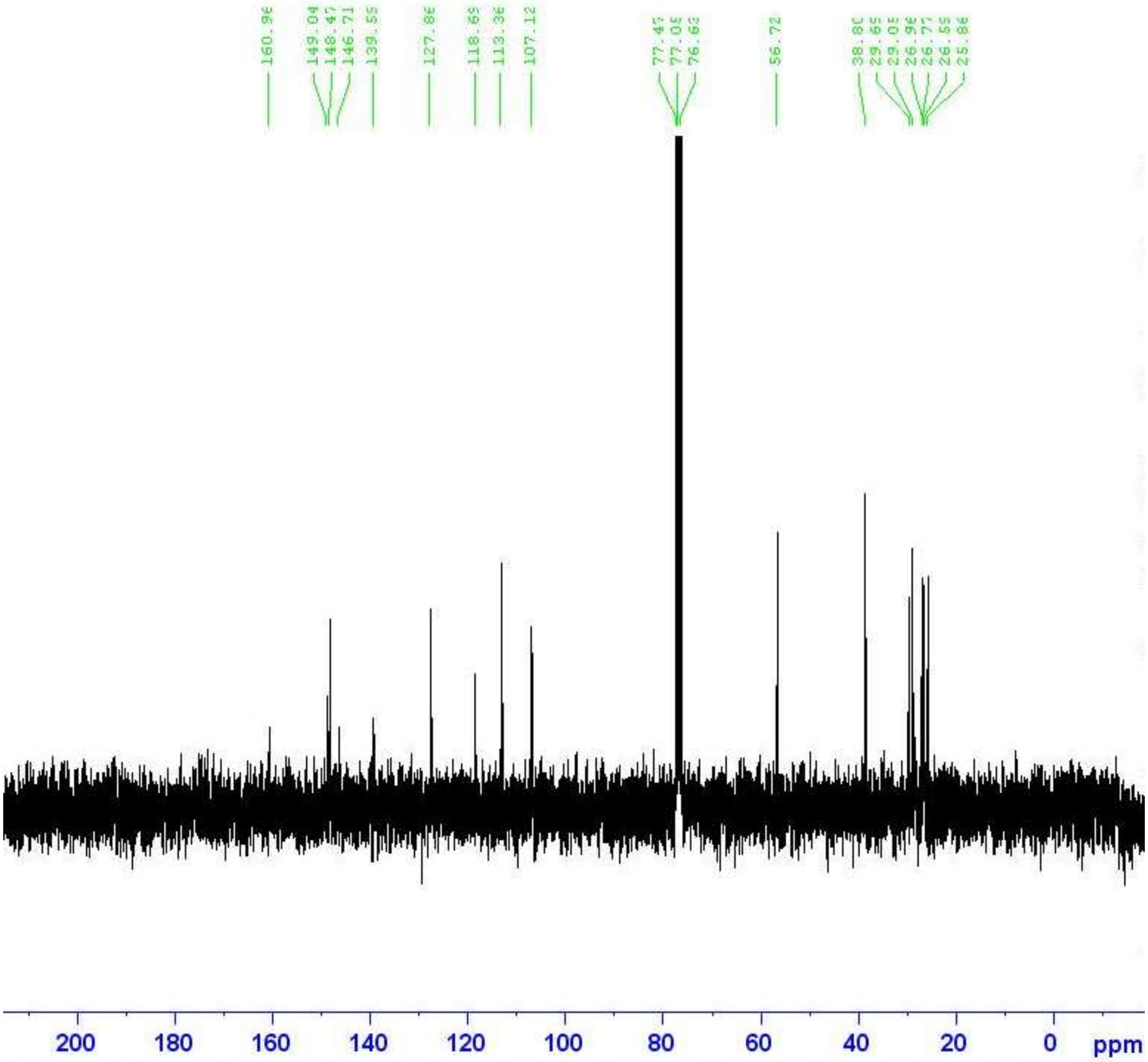

160.96
149.04
148.47
146.71
139.55
127.86
118.69
113.36
107.12
77.47
77.05
76.63
56.72
38.80
29.65
29.05
26.96
26.77
26.55
25.86
200
180
160
140
120
100
80
60
40
20
0
ppm

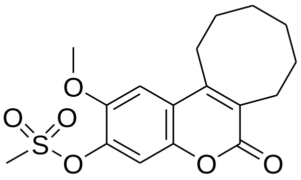

1p

Chemical Formula: $C_{17}H_{20}O_6S$
Exact Mass: 352.10
Molecular Weight: 352.40

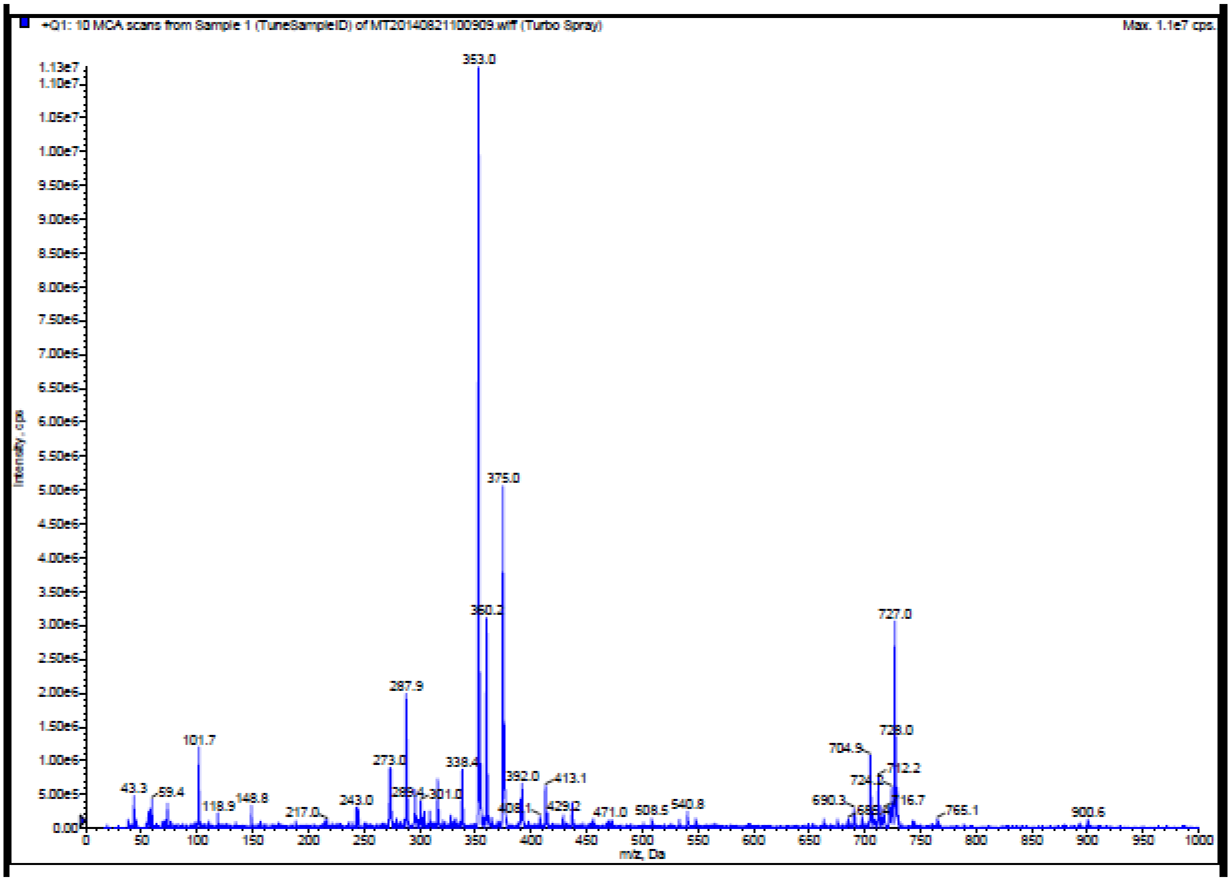

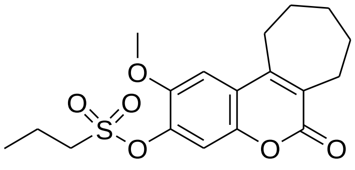

1q

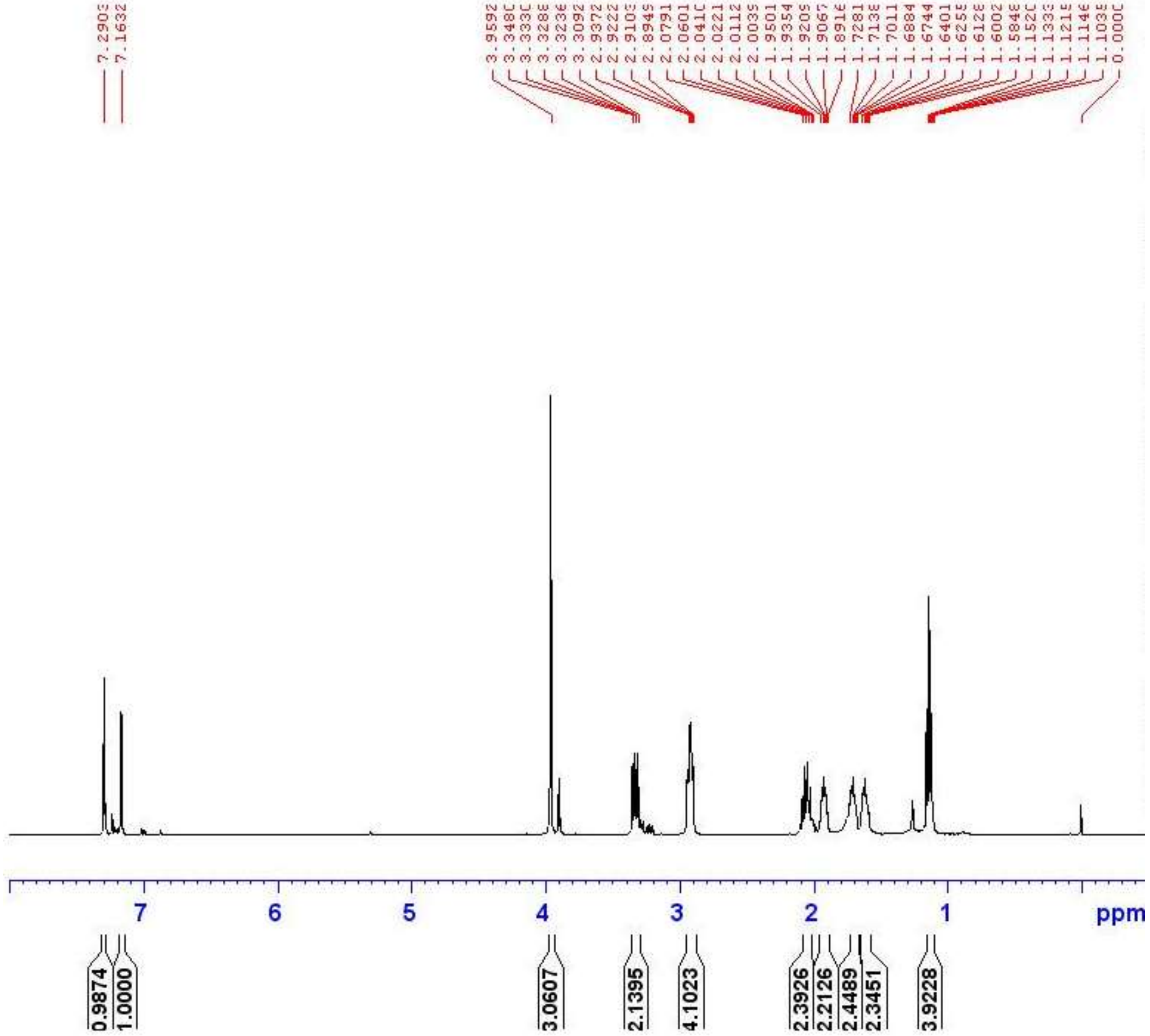
7.2903
7.1632
3.9592
3.3480
3.3330
3.3288
3.3236
3.3092
2.9372
2.9222
2.9103
2.8949
2.0791
2.0601
2.0410
2.0221
2.0112
2.0039
1.9501
1.9354
1.9209
1.9067
1.8916
1.7281
1.7138
1.7011
1.6884
1.6744
1.6401
1.6258
1.6128
1.6002
1.5848
1.1520
1.1333
1.1219
1.1146
1.1035
0.0000
7
6
5
4
3
2
1
ppm
0.9874
1.0000
3.0607
2.1395
4.1023
2.3926
2.2126
2.4489
2.3451
3.9228

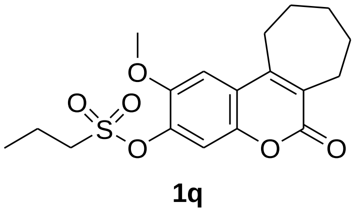
O
O
O
S
O
O
O

1q

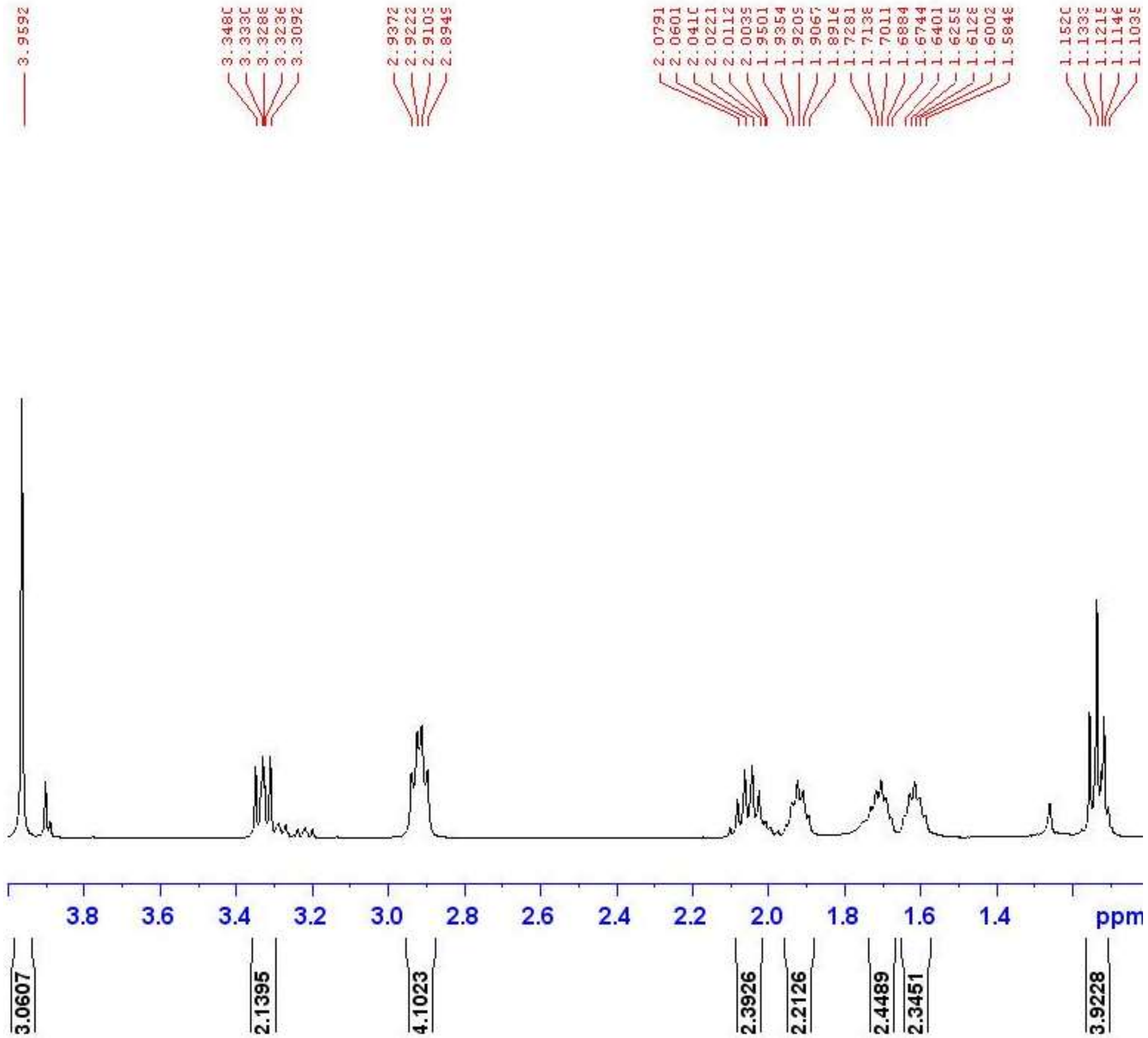
3.9592
3.3480
3.3330
3.3288
3.3236
3.3092
2.9372
2.9222
2.9103
2.8949
2.0791
2.0601
2.0410
2.0221
2.0112
2.0035
1.9501
1.9354
1.9205
1.9067
1.8916
1.7281
1.7138
1.7011
1.6884
1.6744
1.6401
1.6255
1.6128
1.6002
1.5848
1.1520
1.1333
1.1215
1.1146
1.1035
3.8
3.6
3.4
3.2
3.0
2.8
2.6
2.4
2.2
2.0
1.8
1.6
1.4
ppm
3.0607
2.1395
4.1023
2.3926
2.2126
2.4489
2.3451
3.9228

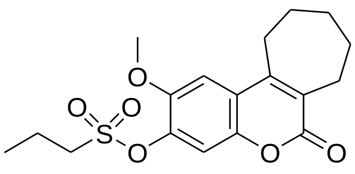

O
O
O
S
O
O
O

1q

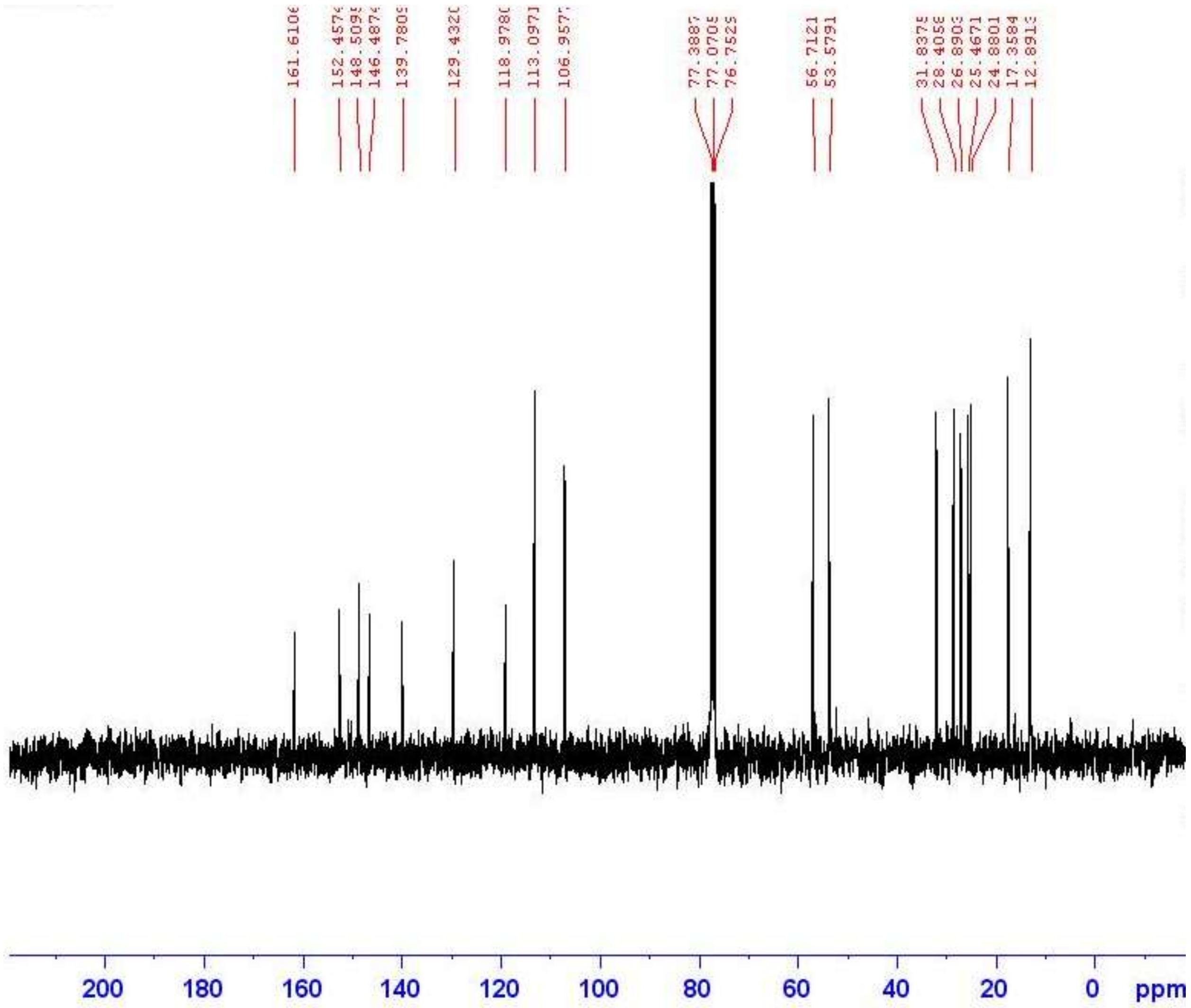

161.610
152.457
148.509
146.487
139.780
129.432
118.978
113.097
106.957
77.388
77.070
76.7525
56.7121
53.5791
31.837
28.405
26.890
25.4671
24.8801
17.3584
12.891
200
180
160
140
120
100
80
60
40
20
0
ppm

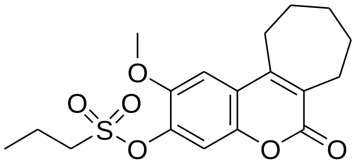

1q

Chemical Formula: $C_{18}H_{22}O_6S$
Exact Mass: 366.11
Molecular Weight: 366.43

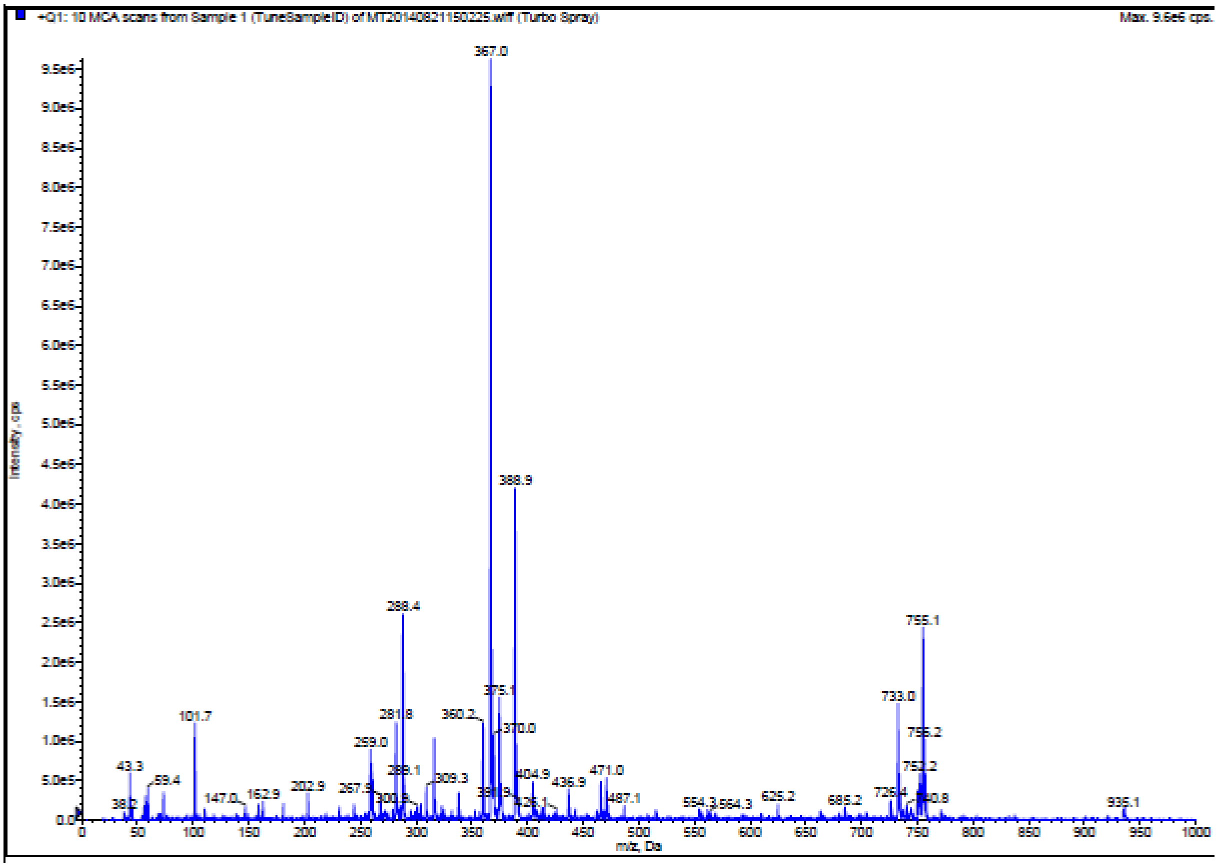

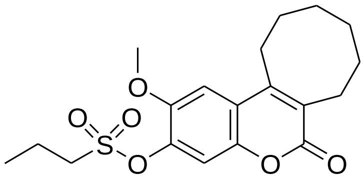

1r

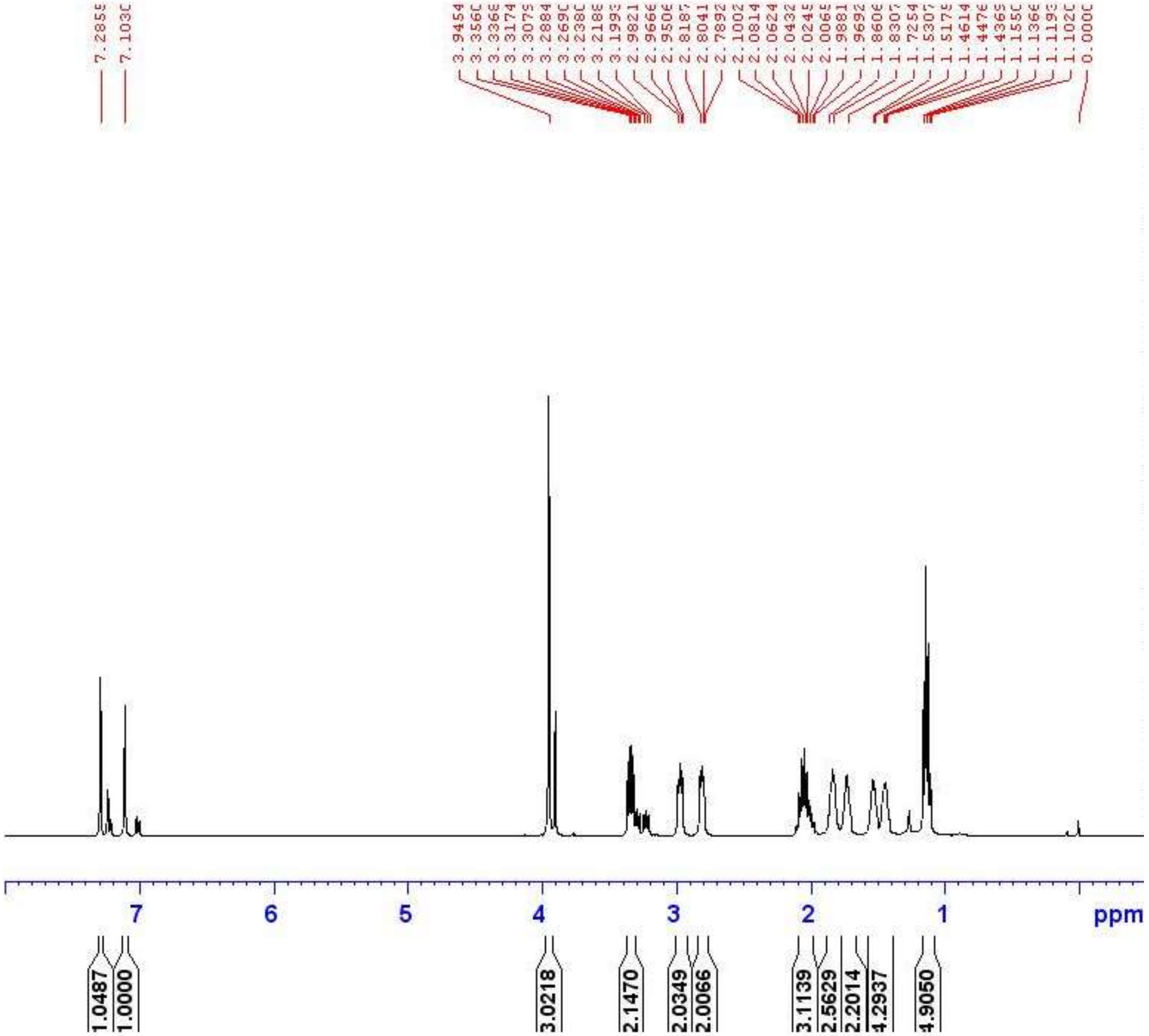

7.2858
7.1030
3.9454
3.3560
3.3368
3.3174
3.3079
3.2884
3.2690
3.2380
3.2188
3.1993
2.9821
2.9666
2.9506
2.8187
2.8041
2.7892
2.1002
2.0814
2.0624
2.0432
2.0249
2.0065
1.9881
1.9692
1.8606
1.8307
1.7254
1.5307
1.5179
1.4614
1.4476
1.4369
1.1550
1.1366
1.1193
1.1020
0.0000
7
6
5
4
3
2
1
ppm
1.0487
1.0000
3.0218
2.1470
2.0349
2.0066
3.1139
2.5629
2.2014
4.2937
4.9050

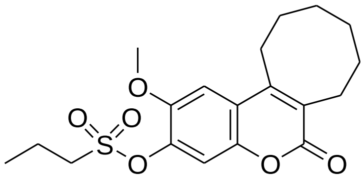

1r

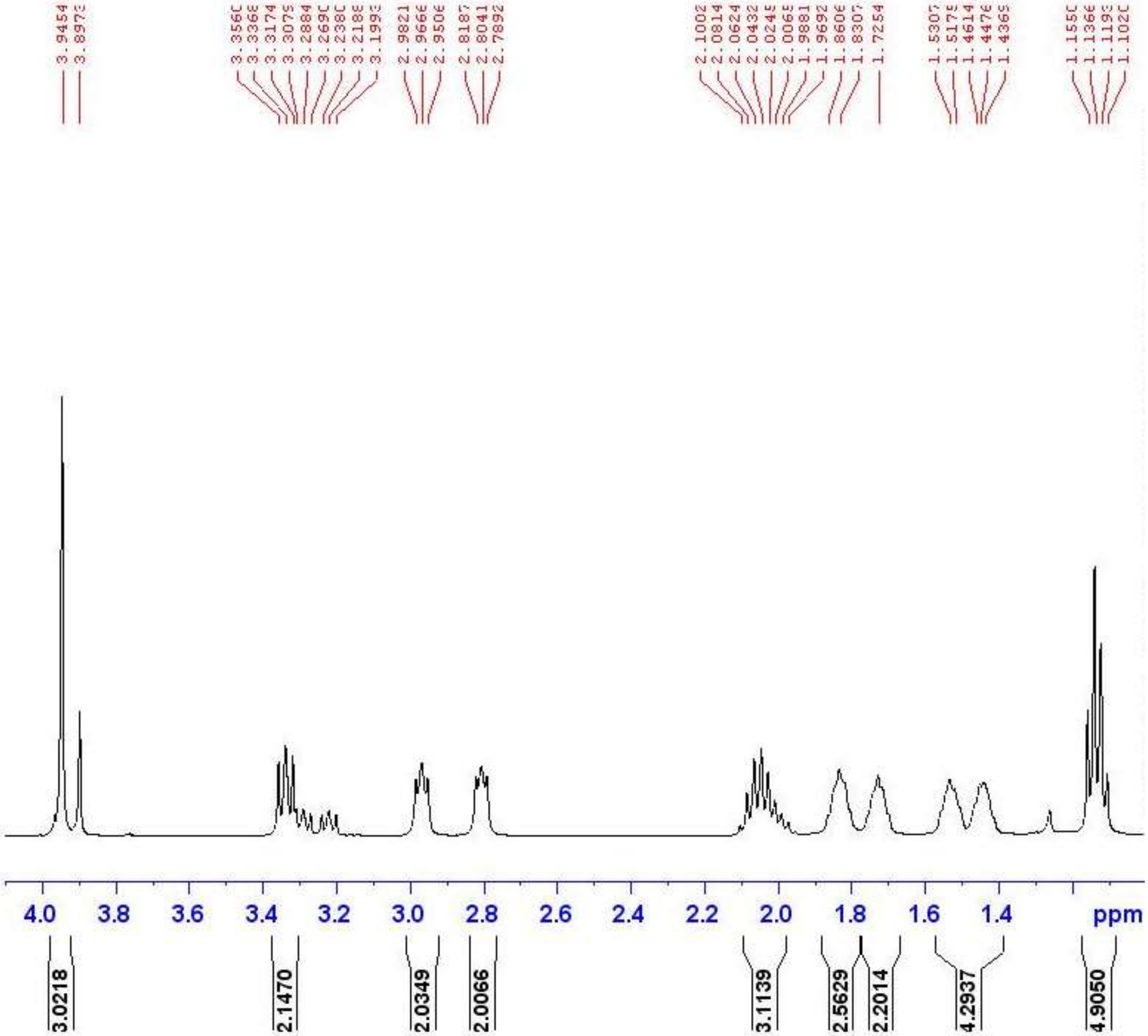

3.9454
3.8973
3.3560
3.3368
3.3174
3.3079
3.2884
3.2690
3.2380
3.2188
3.1993
2.9821
2.9666
2.9506
2.8187
2.8041
2.7892
2.1002
2.0814
2.0624
2.0432
2.0249
2.0065
1.9881
1.9692
1.8606
1.8307
1.7254
1.5307
1.5175
1.4614
1.4476
1.4369
1.1550
1.1366
1.1193
1.1020
4.0
3.8
3.6
3.4
3.2
3.0
2.8
2.6
2.4
2.2
2.0
1.8
1.6
1.4
ppm
3.0218
2.1470
2.0349
2.0066
3.1139
2.5629
2.2014
4.2937
4.9050

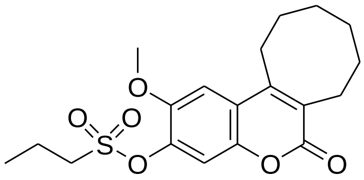

O
O
O
S
O
O
O

1r

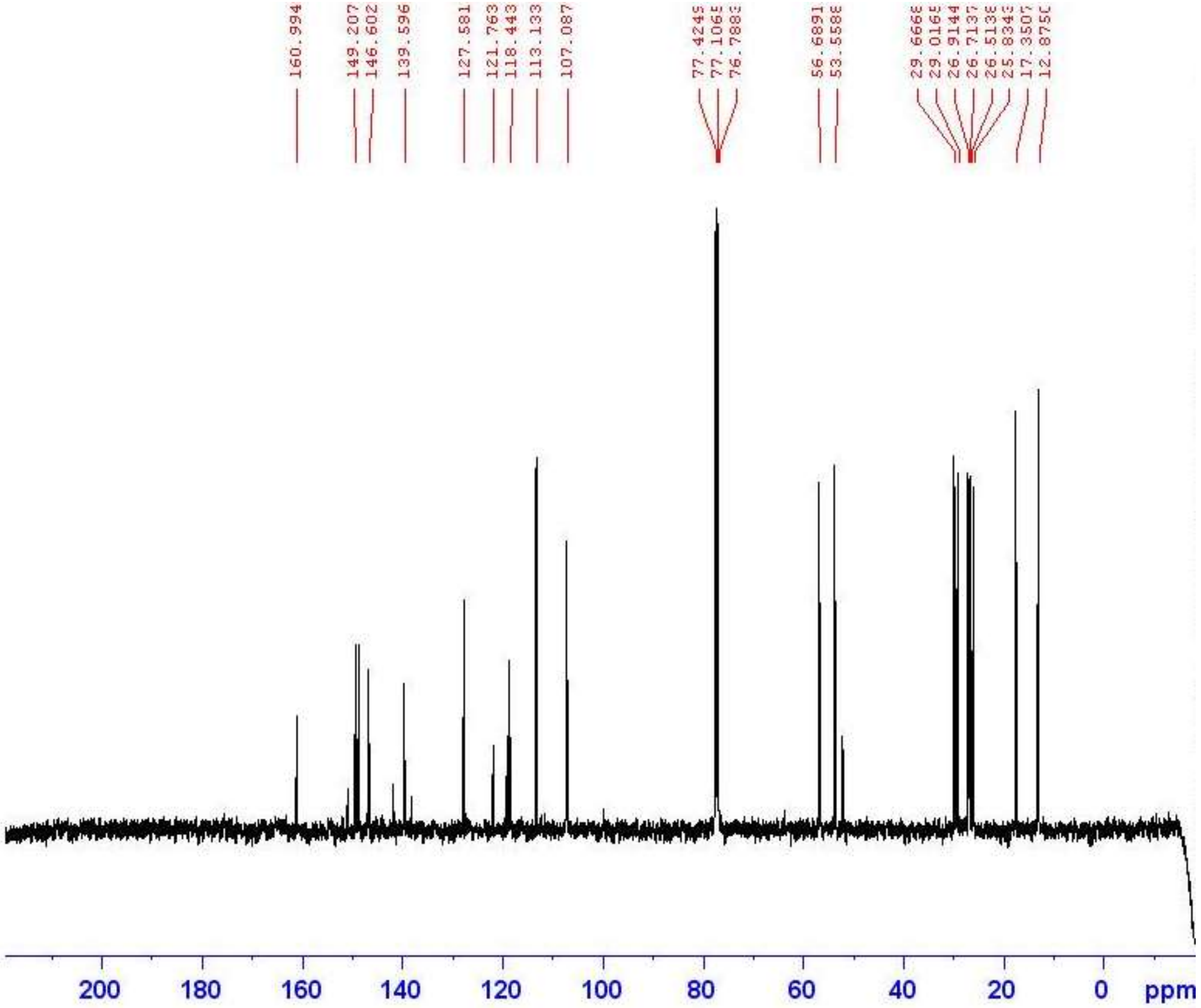

160.994
149.207
146.602
139.596
127.581
121.763
118.443
113.133
107.087
77.4245
77.1065
76.7883
56.6891
53.5588
29.6668
29.0169
26.9144
26.7137
26.5138
25.8343
17.3507
12.8750
200
180
160
140
120
100
80
60
40
20
0
ppm

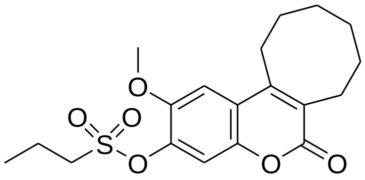

1r

Chemical Formula: $C_{19}H_{24}O_6S$
Exact Mass: 380.13
Molecular Weight: 380.46

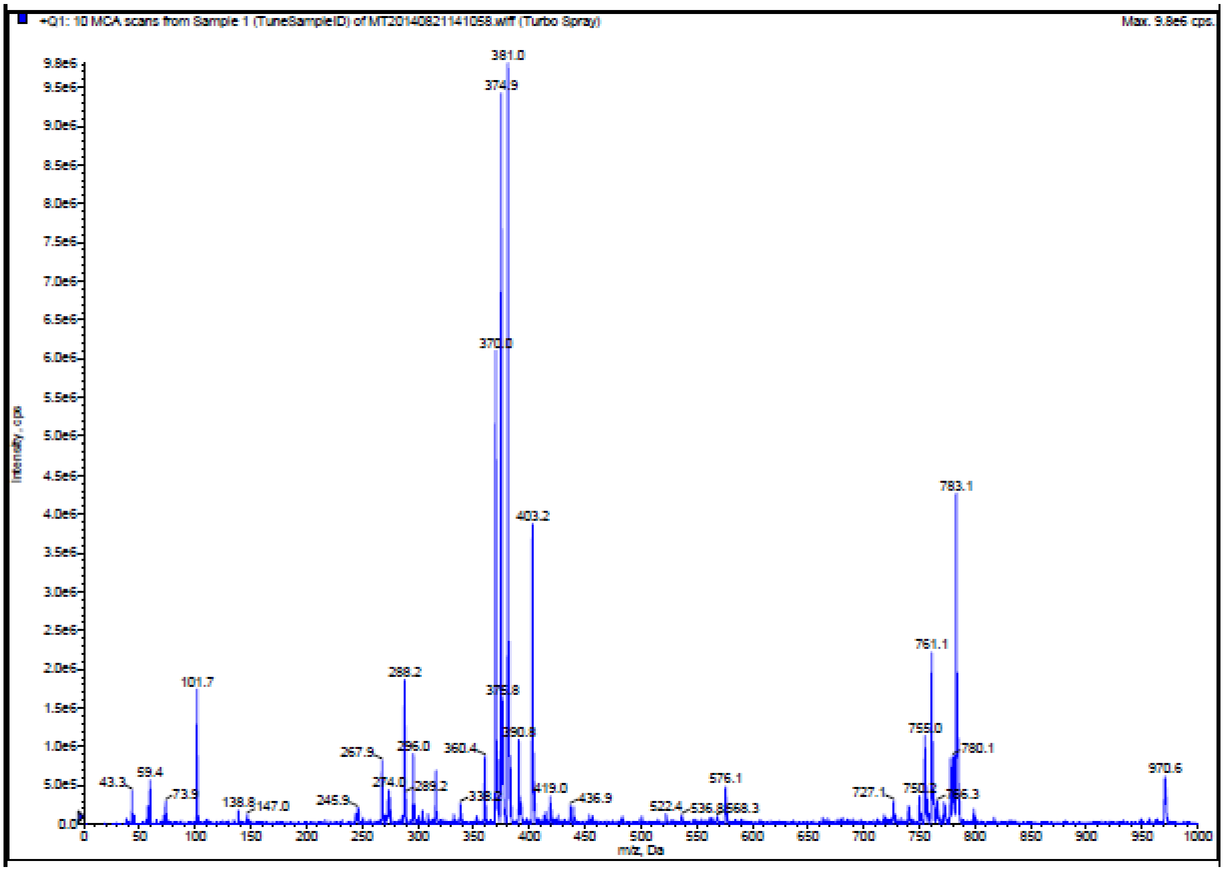

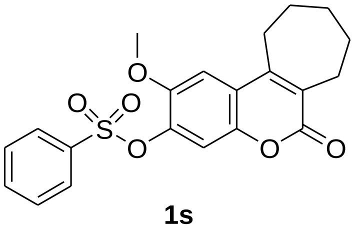
O
O
S
O
O
O
O
O

1s

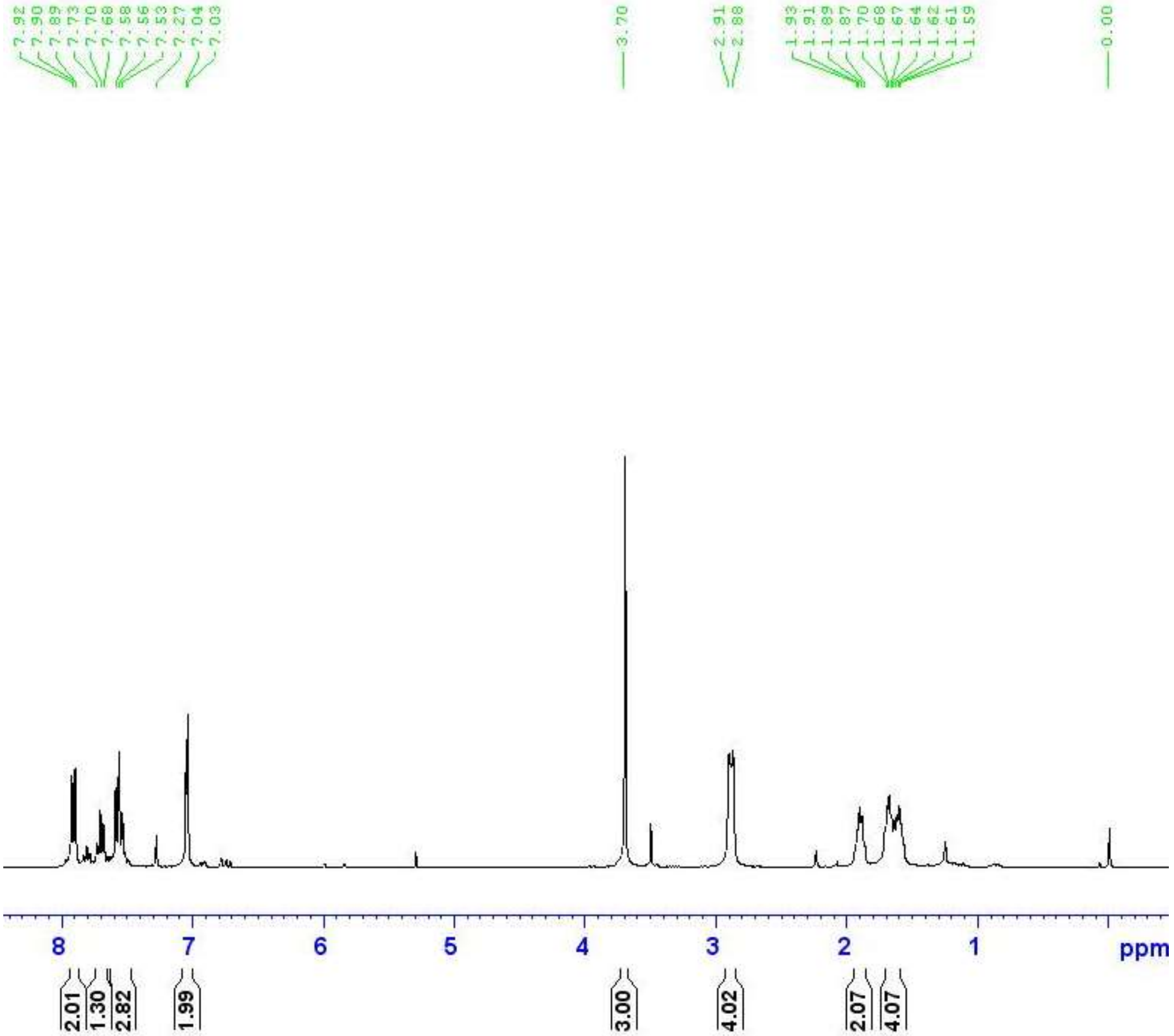
7.92
7.90
7.89
7.73
7.70
7.68
7.58
7.56
7.53
7.27
7.04
7.03
3.70
2.91
2.88
1.93
1.91
1.89
1.87
1.70
1.68
1.67
1.64
1.62
1.61
1.59
0.00
8
7
6
5
4
3
2
1
ppm
2.01
1.30
2.82
1.99
3.00
4.02
2.07
4.07

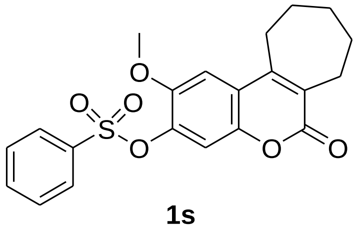

1s

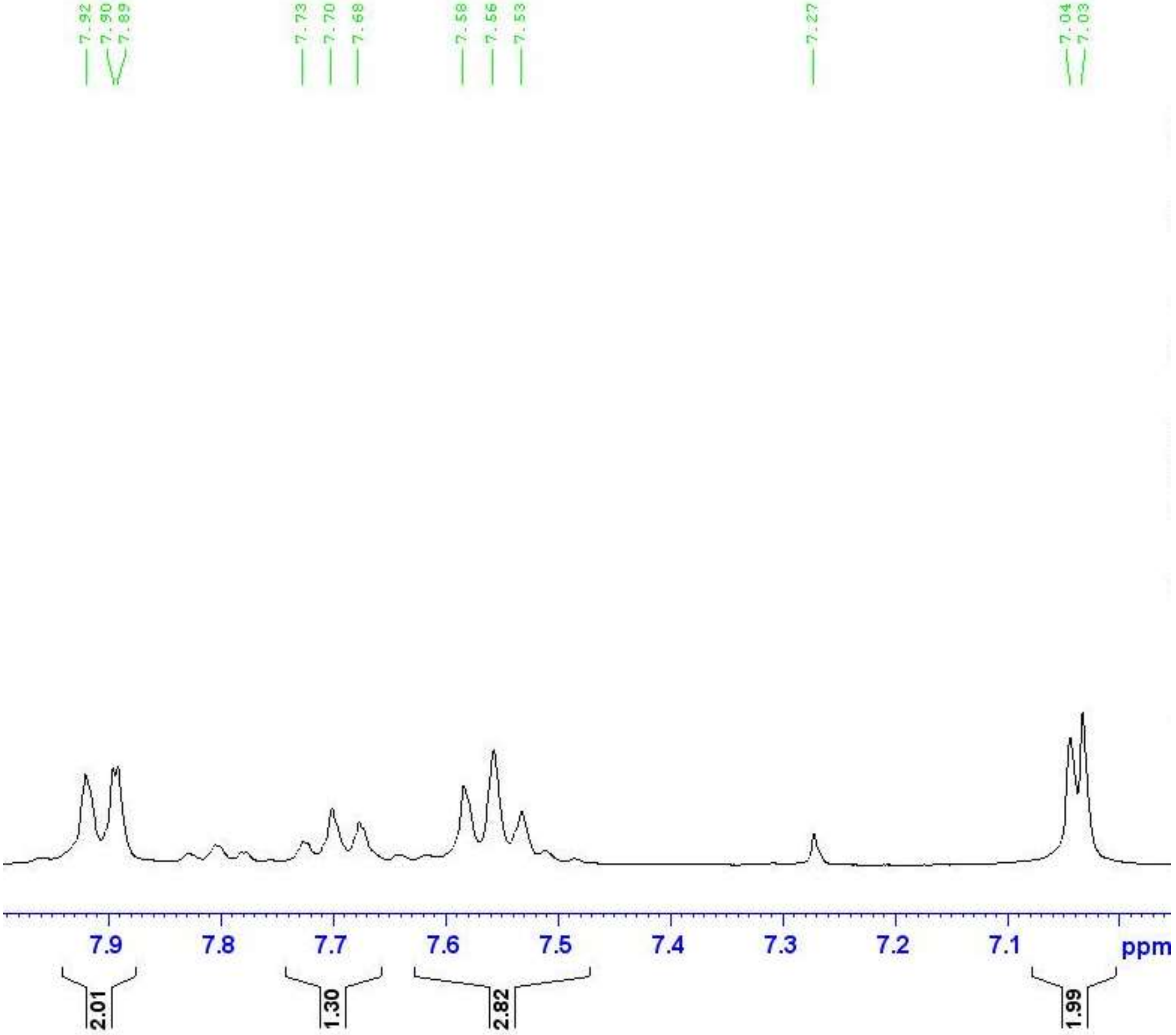
7.92
7.90
7.89
7.73
7.70
7.68
7.58
7.56
7.53
7.27
7.04
7.03
7.9
7.8
7.7
7.6
7.5
7.4
7.3
7.2
7.1
ppm
2.01
1.30
2.82
1.99

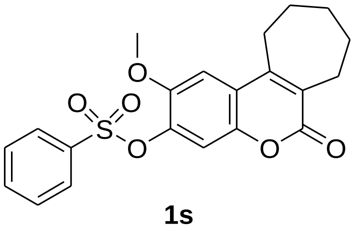

1s

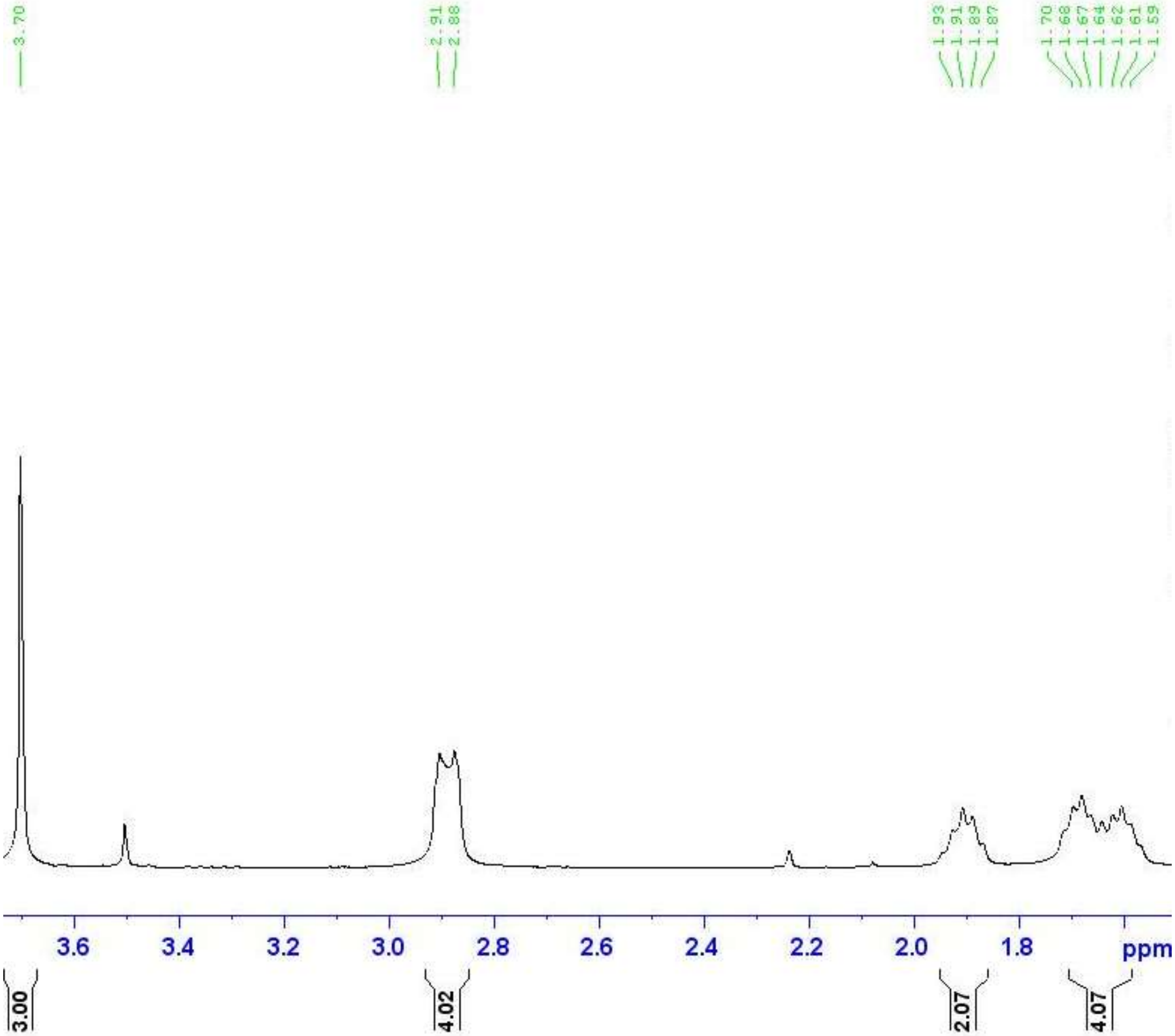

3.70
2.91
2.88
1.93
1.91
1.89
1.87
1.70
1.68
1.67
1.64
1.62
1.61
1.59
3.6
3.4
3.2
3.0
2.8
2.6
2.4
2.2
2.0
1.8
ppm
3.00
4.02
2.07
4.07

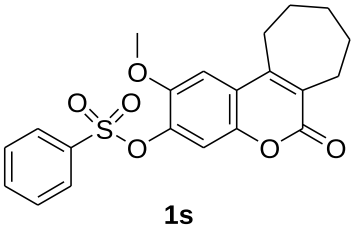

1s

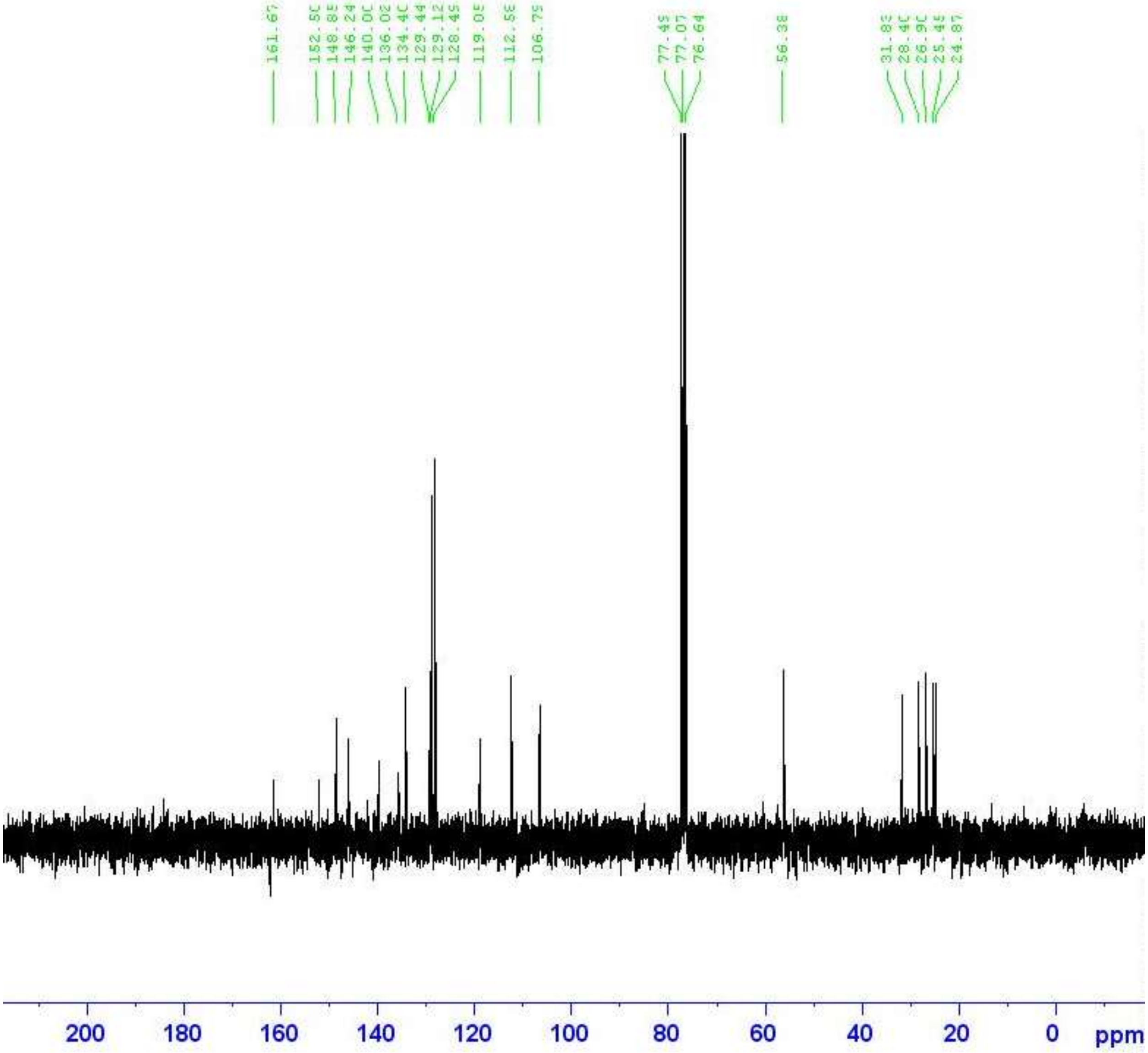

161.67
152.50
148.85
146.24
140.00
136.08
134.40
129.44
129.12
128.45
119.05
112.58
106.79
77.49
77.07
76.64
56.38
31.89
28.40
26.90
25.49
24.87
200
180
160
140
120
100
80
60
40
20
0
ppm

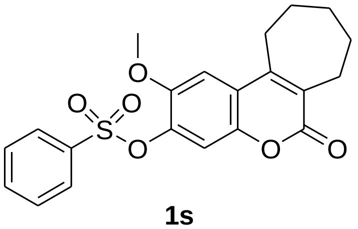

1s

Chemical Formula: $C_{21}H_{20}O_6S$
Exact Mass: 400.10
Molecular Weight: 400.44

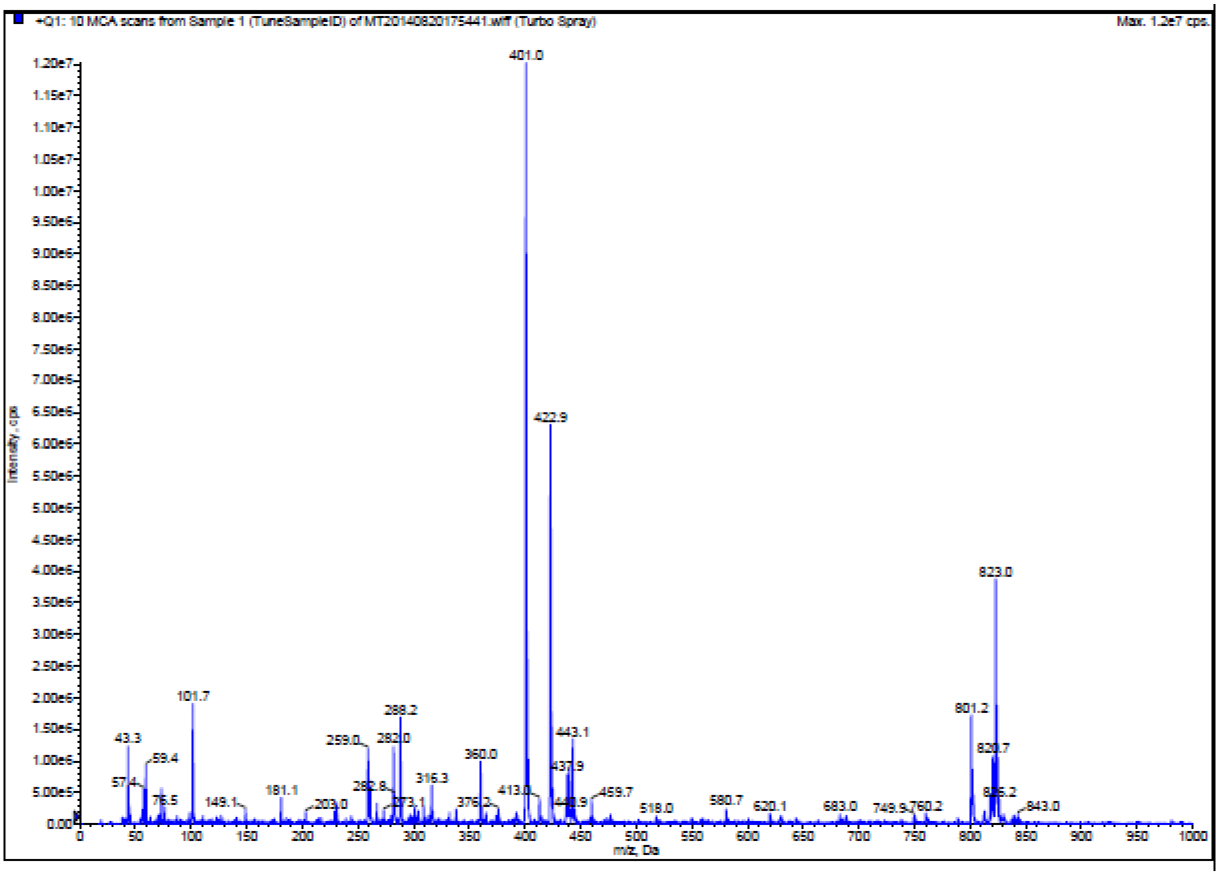

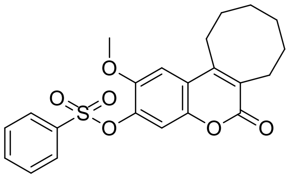
O
O
S
O
O
O
O

1t

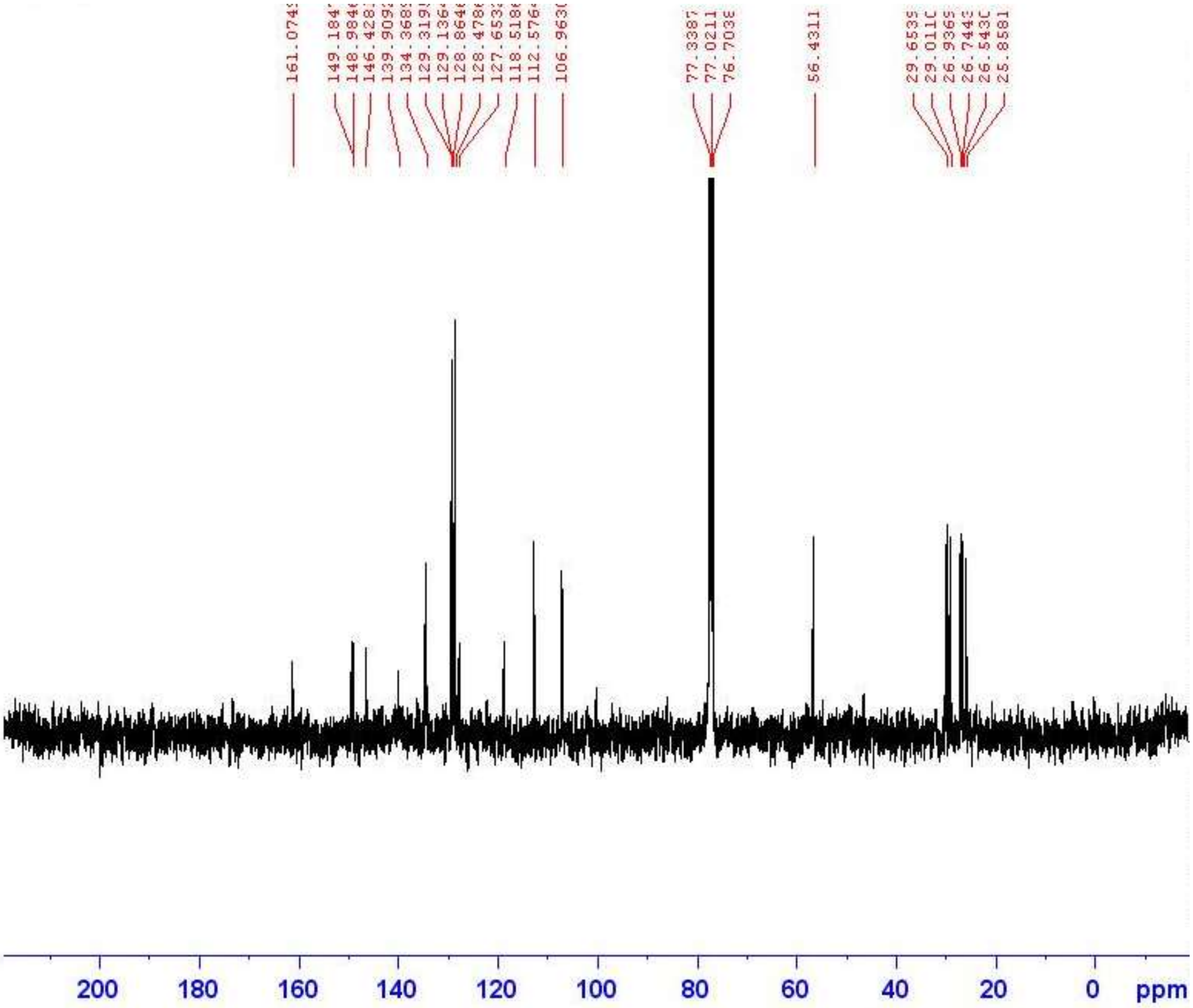
77.3387
77.0211
76.7038
56.4311
25.8581
200
180
160
140
120
100
80
60
40
20
0
ppm

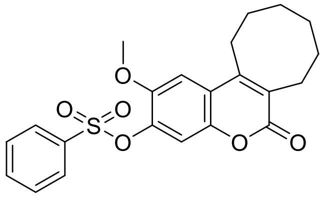

Chemical Formula: $C_{22}H_{22}O_6S$
Exact Mass: 414.11
Molecular Weight: 414.47

1t

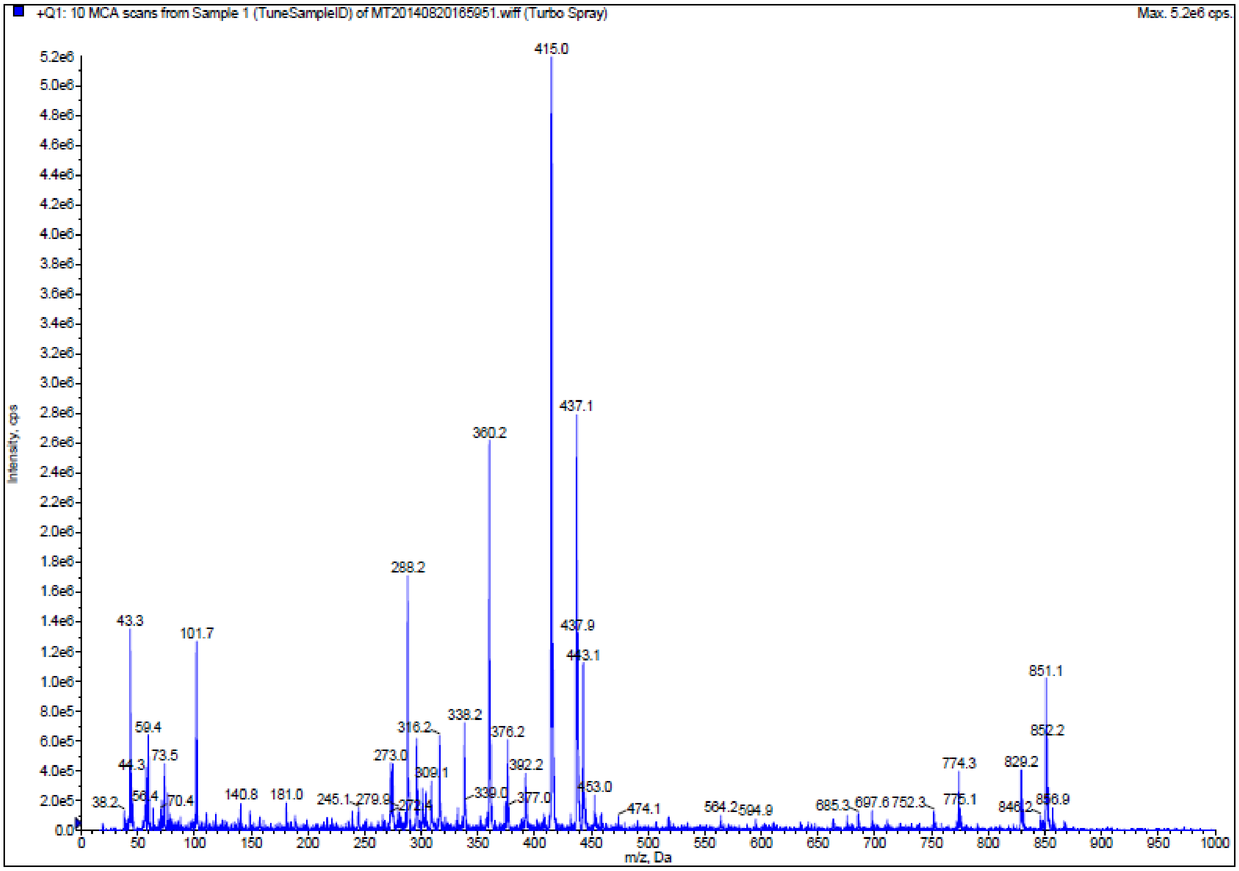

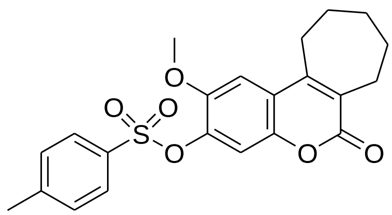

1u

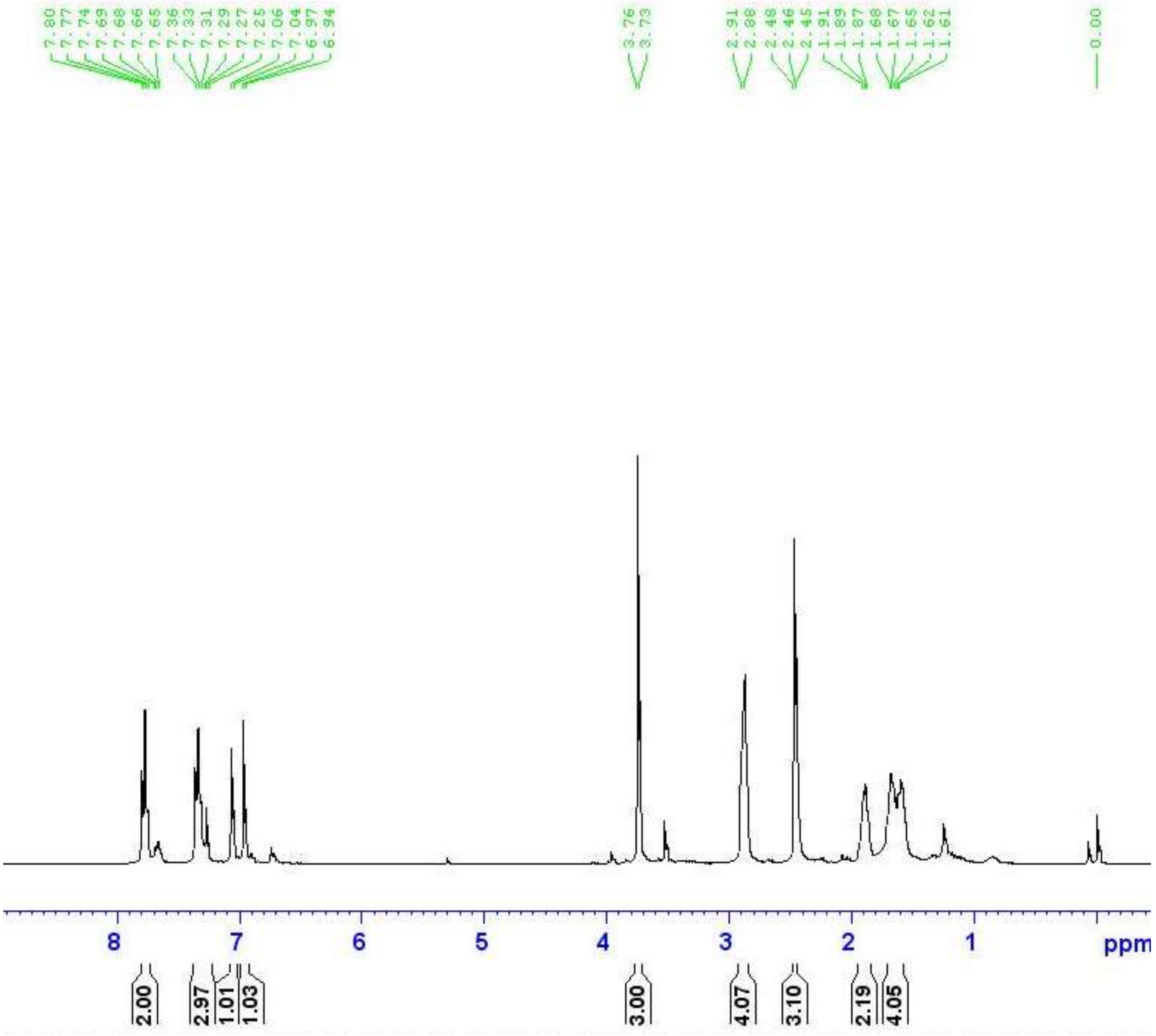

7.80 7.77 7.74 7.69 7.68 7.66 7.65 7.36 7.33 7.31 7.29 7.27 7.25 7.06 7.04 6.97 6.94
3.76 3.73
2.91 2.88 2.48 2.46 2.45 1.91 1.89 1.87 1.68 1.67 1.65 1.62 1.61
0.00
8 7 6 5 4 3 2 1 ppm
2.00 2.97 1.01 1.03 3.00 4.07 3.10 2.19 4.05

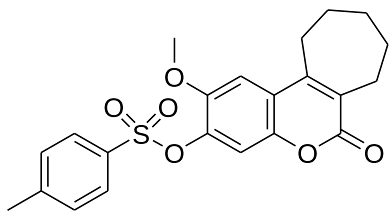

1u

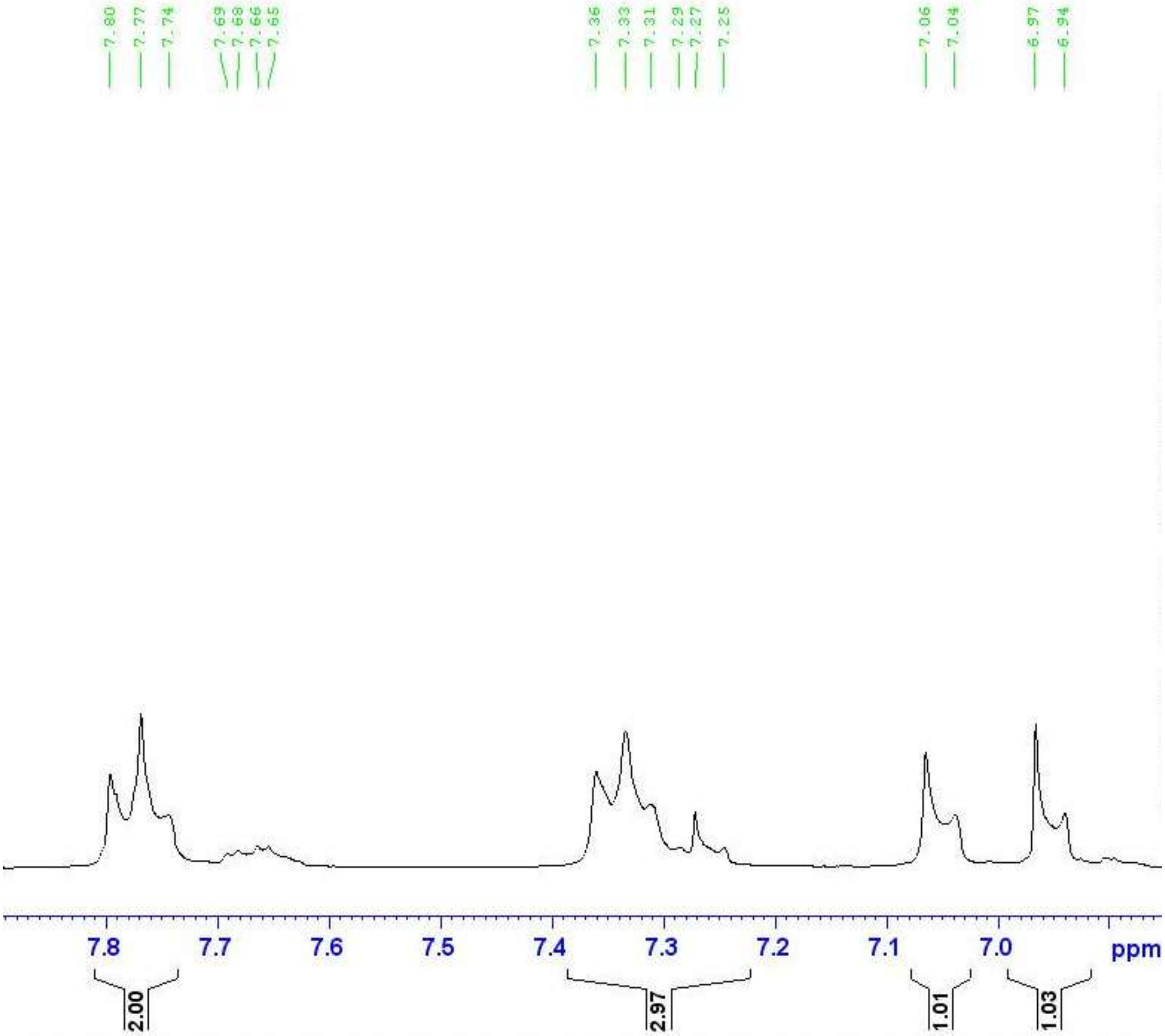
7.80
7.77
7.74
7.69
7.68
7.66
7.65
7.36
7.33
7.31
7.29
7.27
7.25
7.06
7.04
6.97
6.94
7.8
7.7
7.6
7.5
7.4
7.3
7.2
7.1
7.0
ppm
2.00
2.97
1.01
1.03

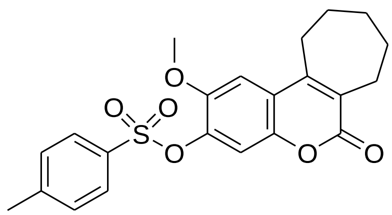
O
O
O
S
O
O
O
O

1u

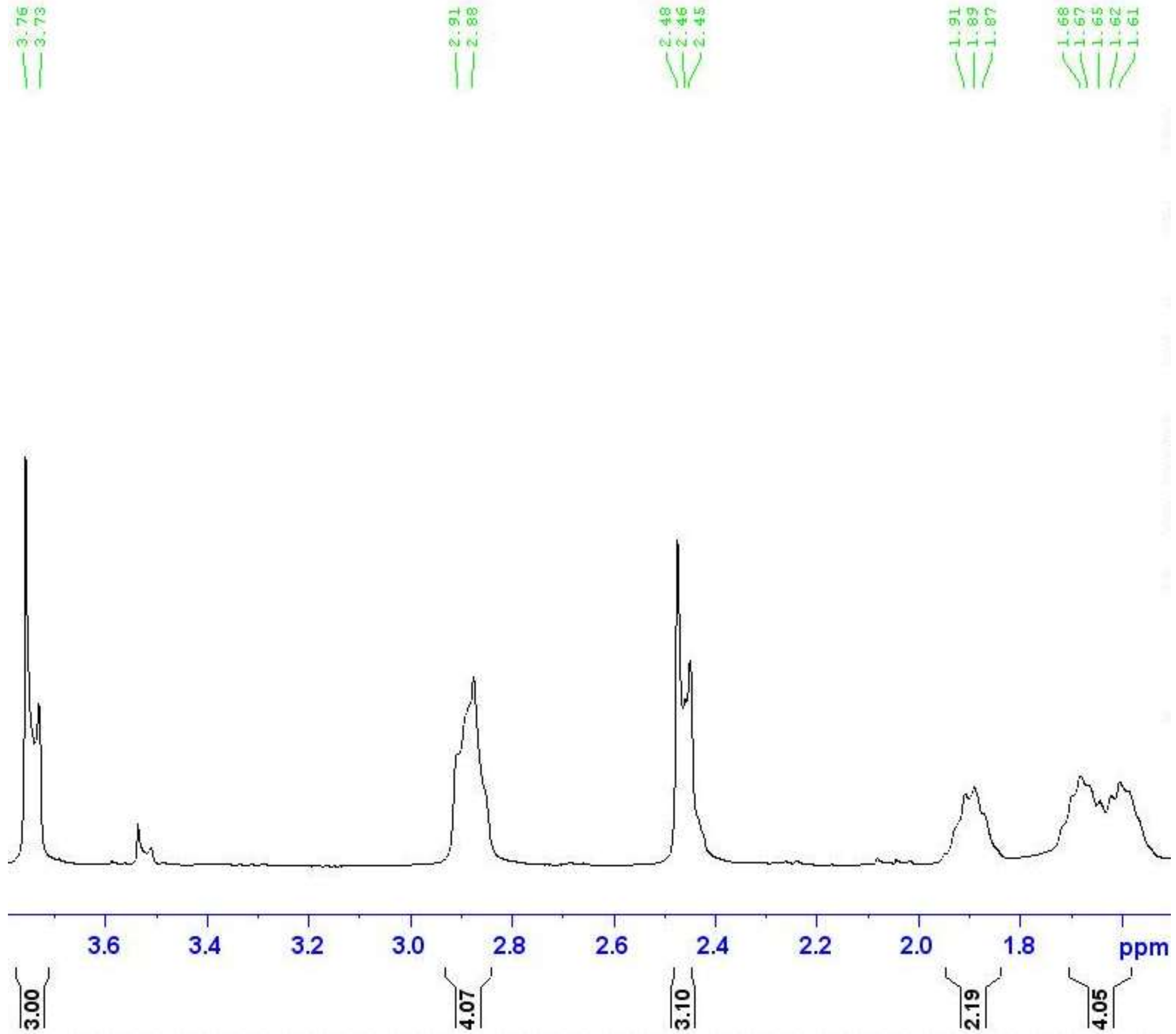
3.76
3.73
2.91
2.88
2.48
2.46
2.45
1.91
1.89
1.87
1.68
1.67
1.65
1.62
1.61
3.6
3.4
3.2
3.0
2.8
2.6
2.4
2.2
2.0
1.8
ppm
3.00
4.07
3.10
2.19
4.05

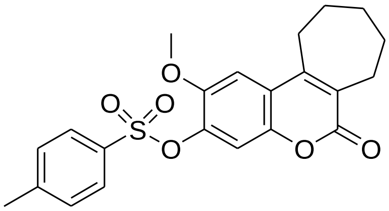

O
O
S
O
O
O
O

1u

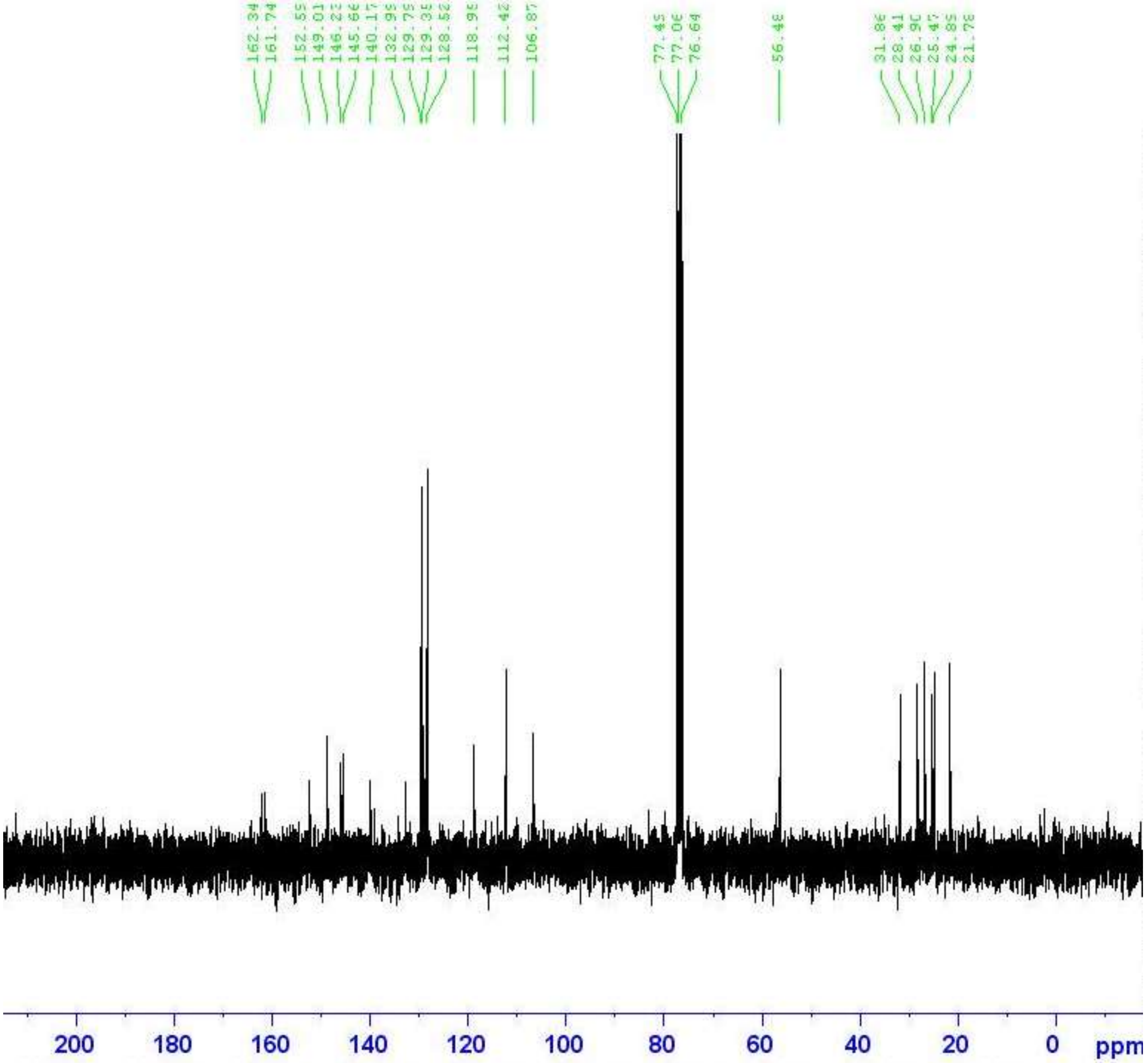

162.34
161.74
152.55
149.01
146.23
145.66
140.17
132.99
129.75
129.35
128.52
118.95
112.42
106.87
77.45
77.06
76.64
56.48
31.86
28.41
26.90
25.47
24.85
21.78
200 180 160 140 120 100 80 60 40 20 0 ppm

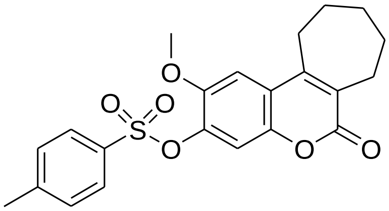

1u

Chemical Formula: $C_{22}H_{22}O_6S$
Exact Mass: 414.11
Molecular Weight: 414.47

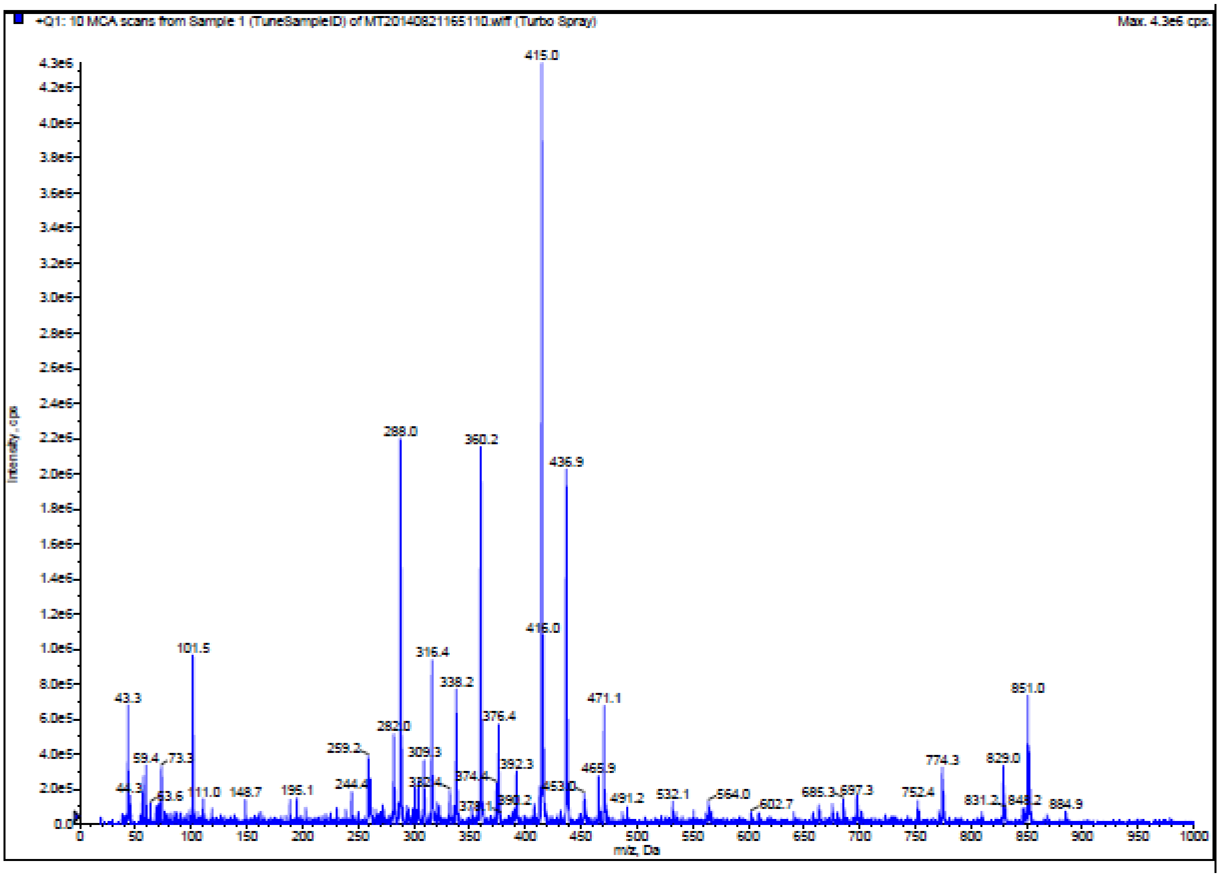

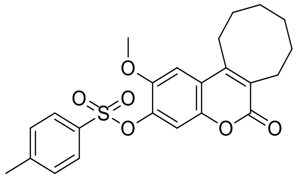

1v

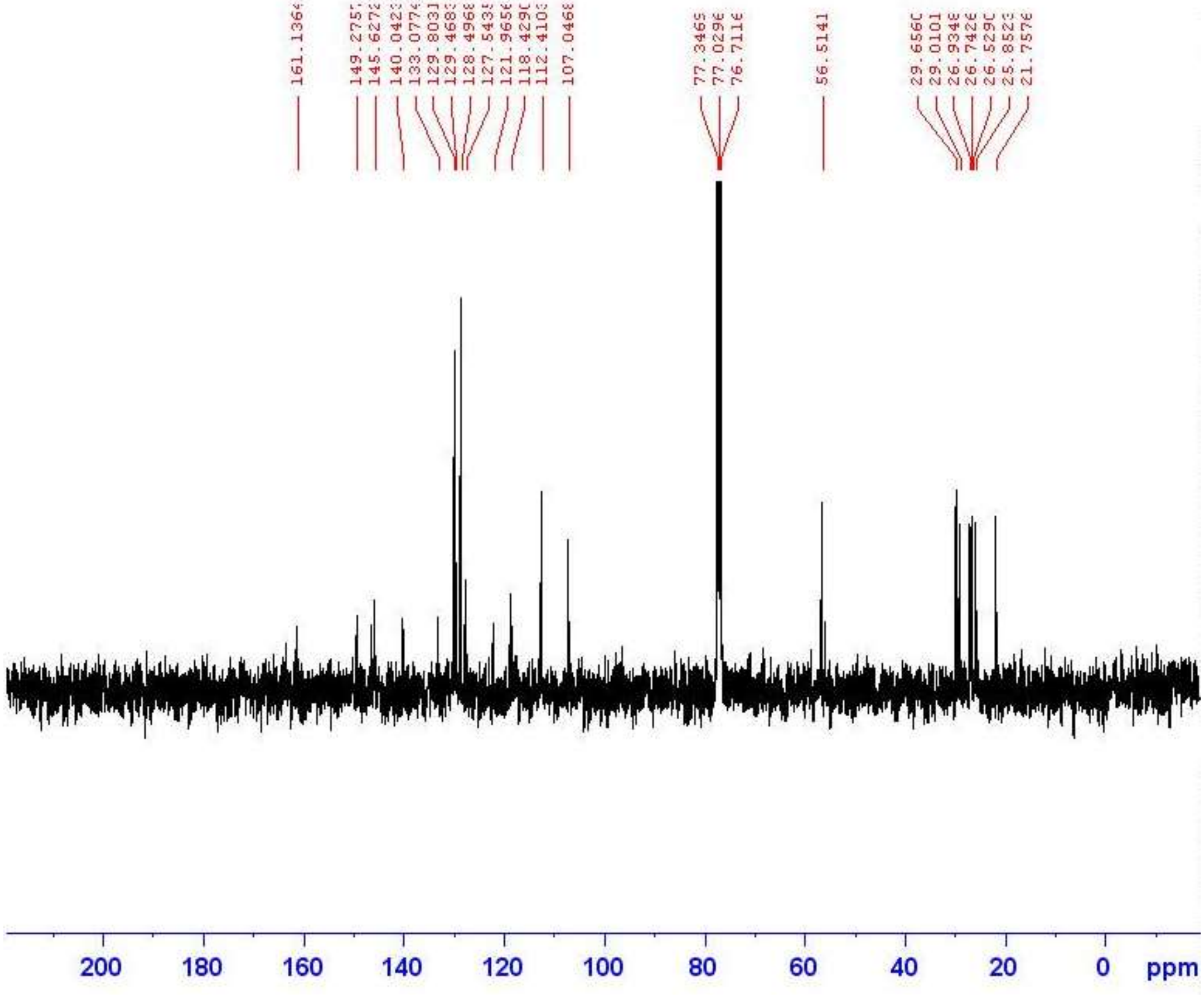
161.1364
149.2757
145.6272
140.0423
133.0774
129.8031
129.4683
128.4968
127.5435
121.9656
118.4290
112.4103
107.0468
77.3469
77.0296
76.7116
56.5141
29.6560
29.0101
26.9348
26.7426
26.5290
25.8523
21.7576
200
180
160
140
120
100
80
60
40
20
0
ppm

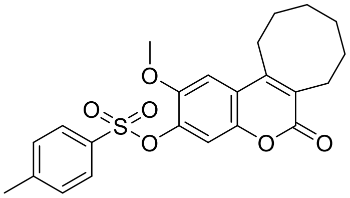

Chemical Formula: $C_{23}H_{24}O_6S$
Exact Mass: 428.13
Molecular Weight: 428.50

1v

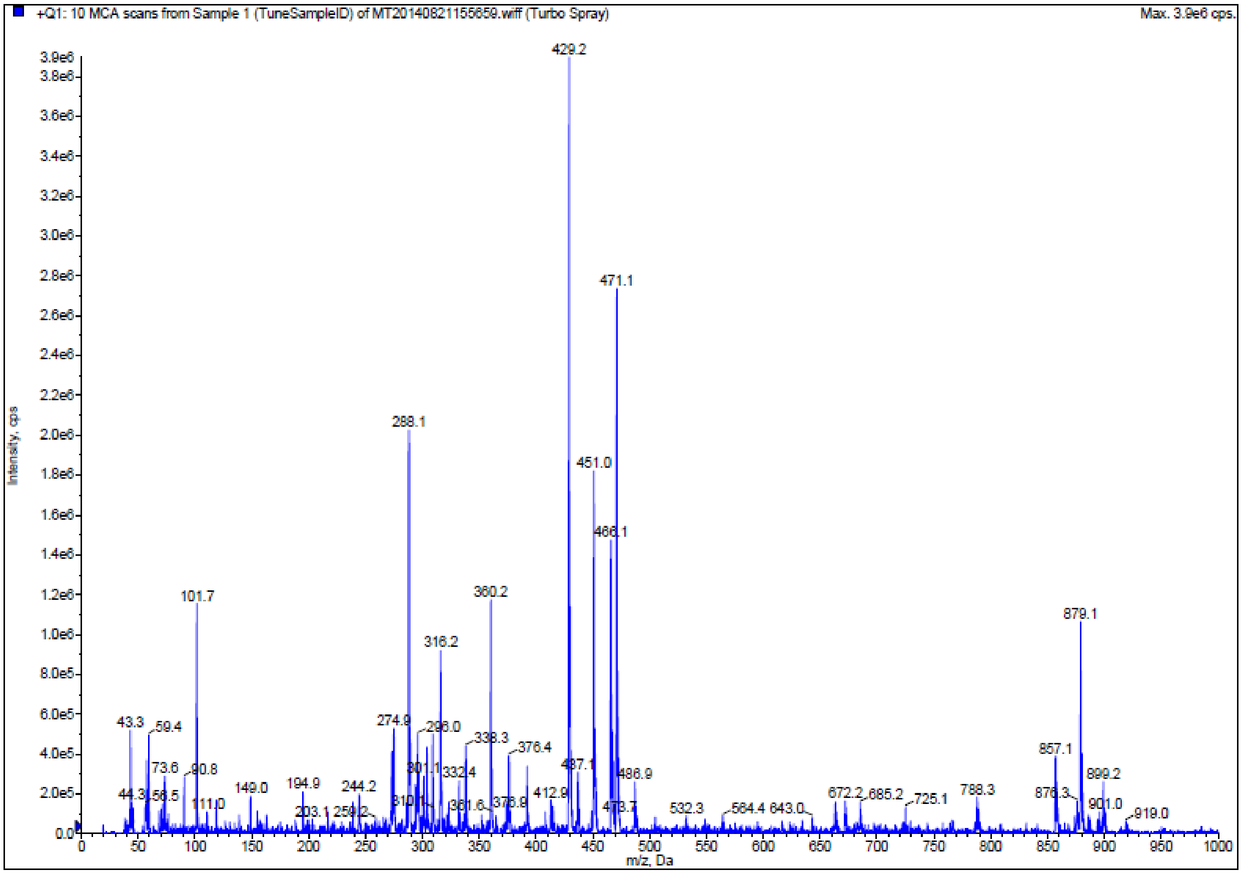

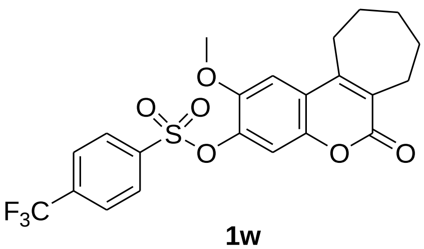
O
O
O
S
O
O
O
O
F3C

1w

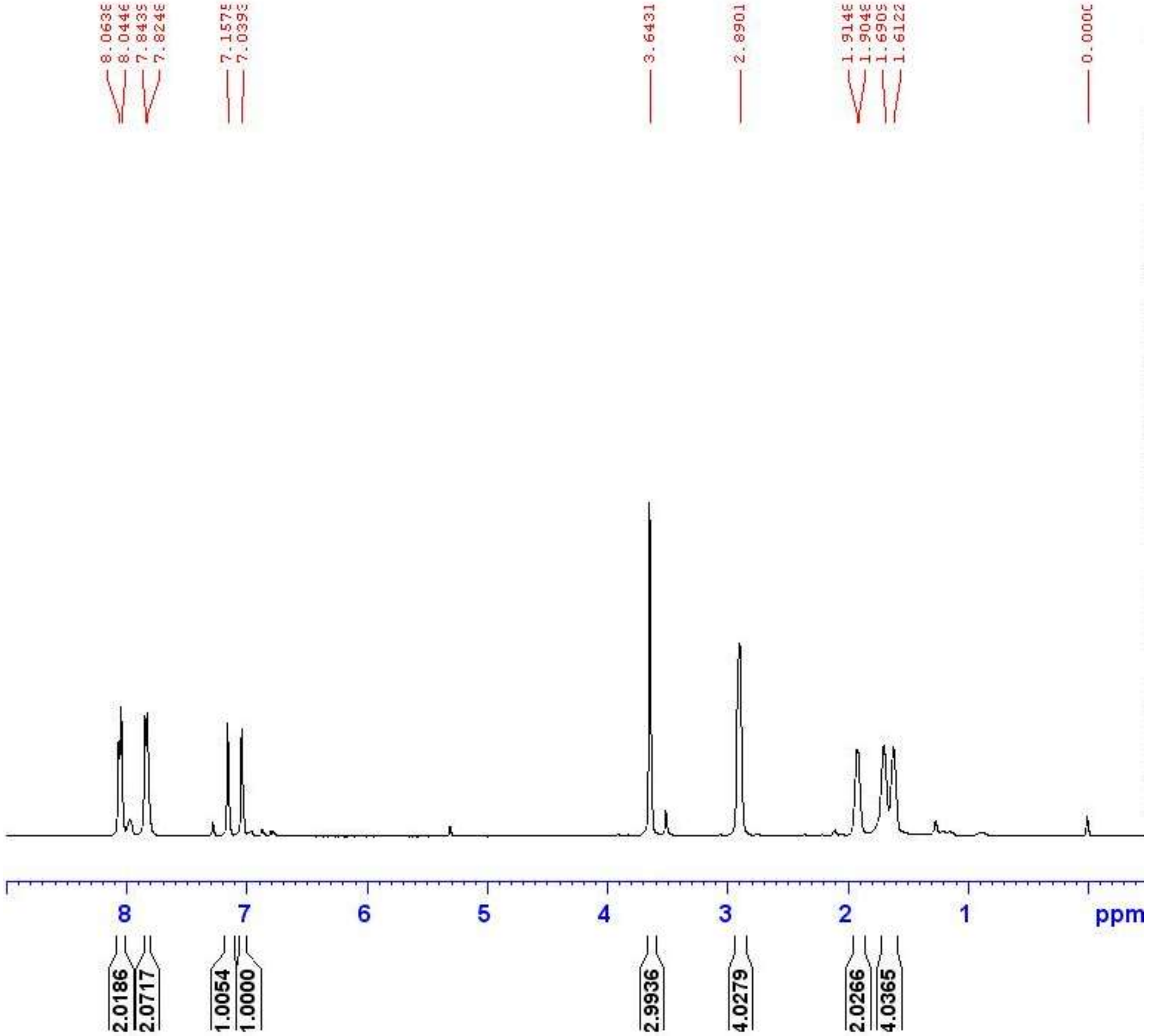
8.0638
8.0446
7.8439
7.8248
7.1579
7.0393
3.6431
2.8901
1.9148
1.9048
1.6909
1.6122
0.0000
8
7
6
5
4
3
2
1
ppm
2.0186
2.0717
1.0054
1.0000
2.9936
4.0279
2.0266
4.0365

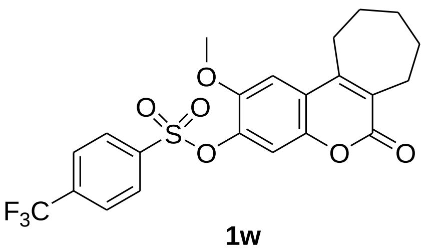
O
O
O
S
O
O
O
F3C

1w

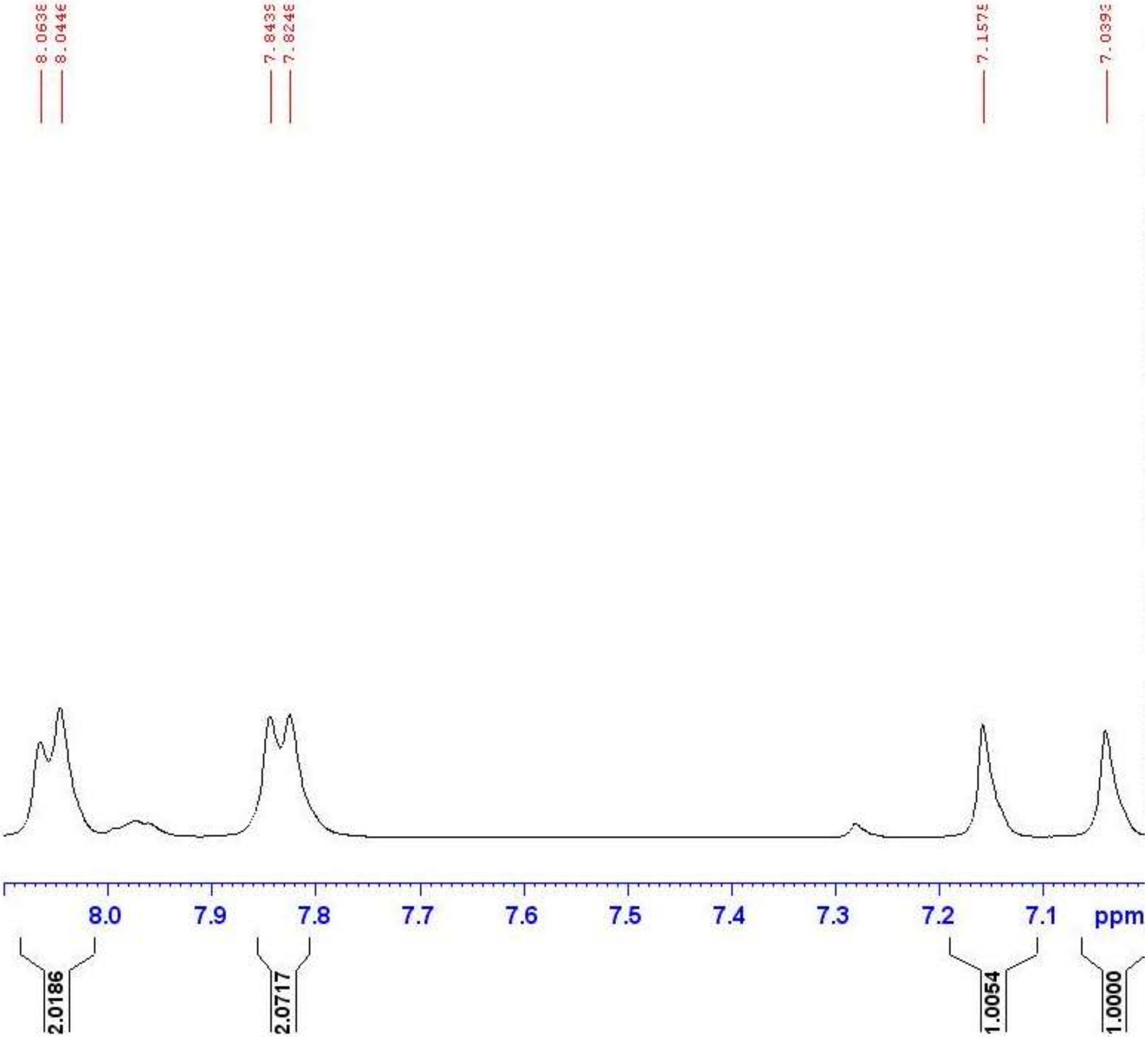
8.0638
8.0446
7.8439
7.8248
7.1579
7.0393
8.0
7.9
7.8
7.7
7.6
7.5
7.4
7.3
7.2
7.1
ppm
2.0186
2.0717
1.0054
1.0000

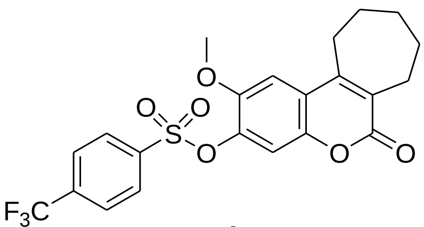
O
O
S
O
O
O
O
F_3C

1w

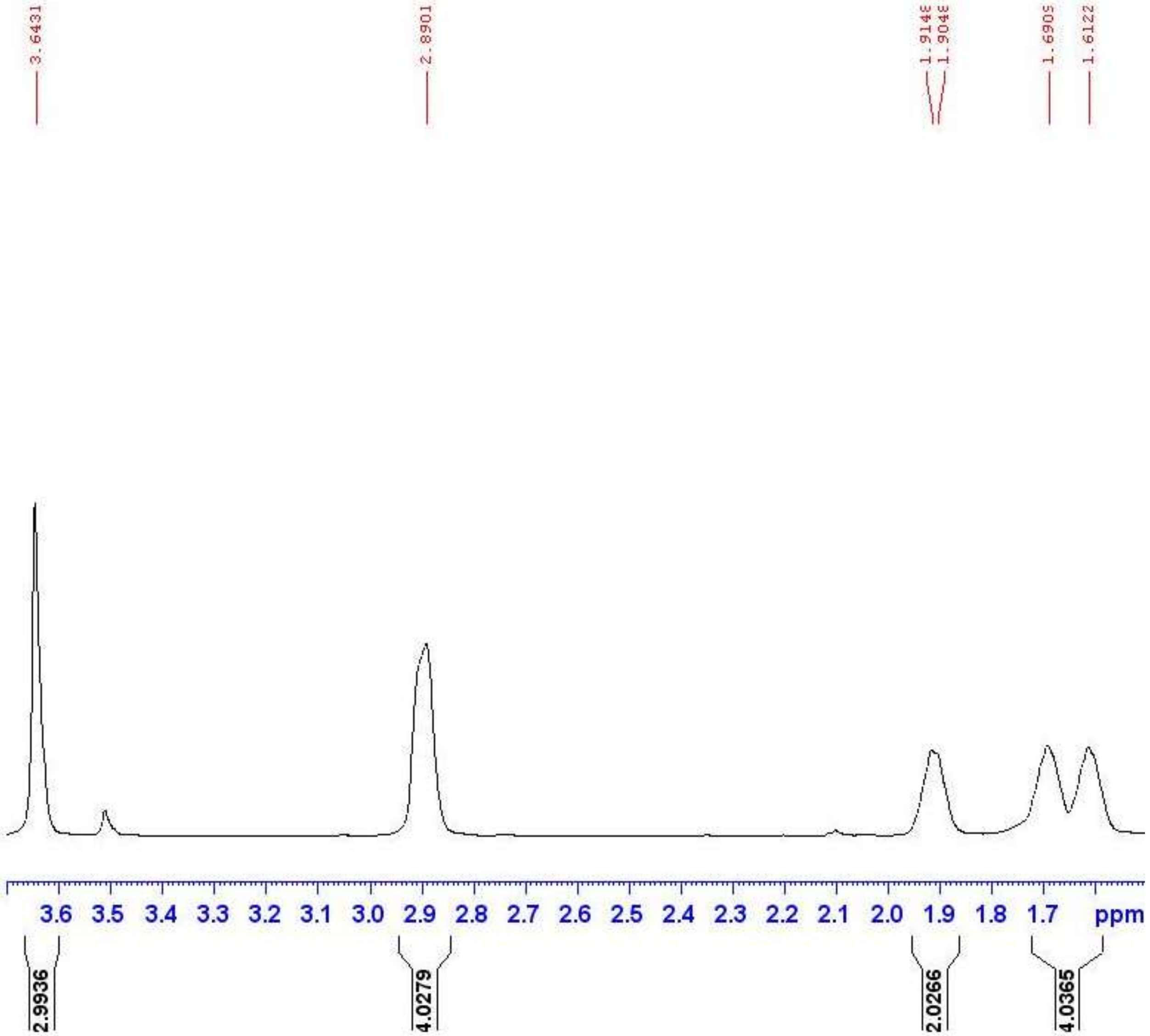
3.6431
2.8901
1.9148
1.9048
1.6905
1.6122
3.6 3.5 3.4 3.3 3.2 3.1 3.0 2.9 2.8 2.7 2.6 2.5 2.4 2.3 2.2 2.1 2.0 1.9 1.8 1.7 ppm
2.9936
4.0279
2.0266
4.0365

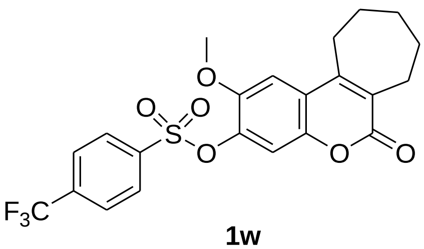

O
O
S
O
O
O
O
O
F3C

1w

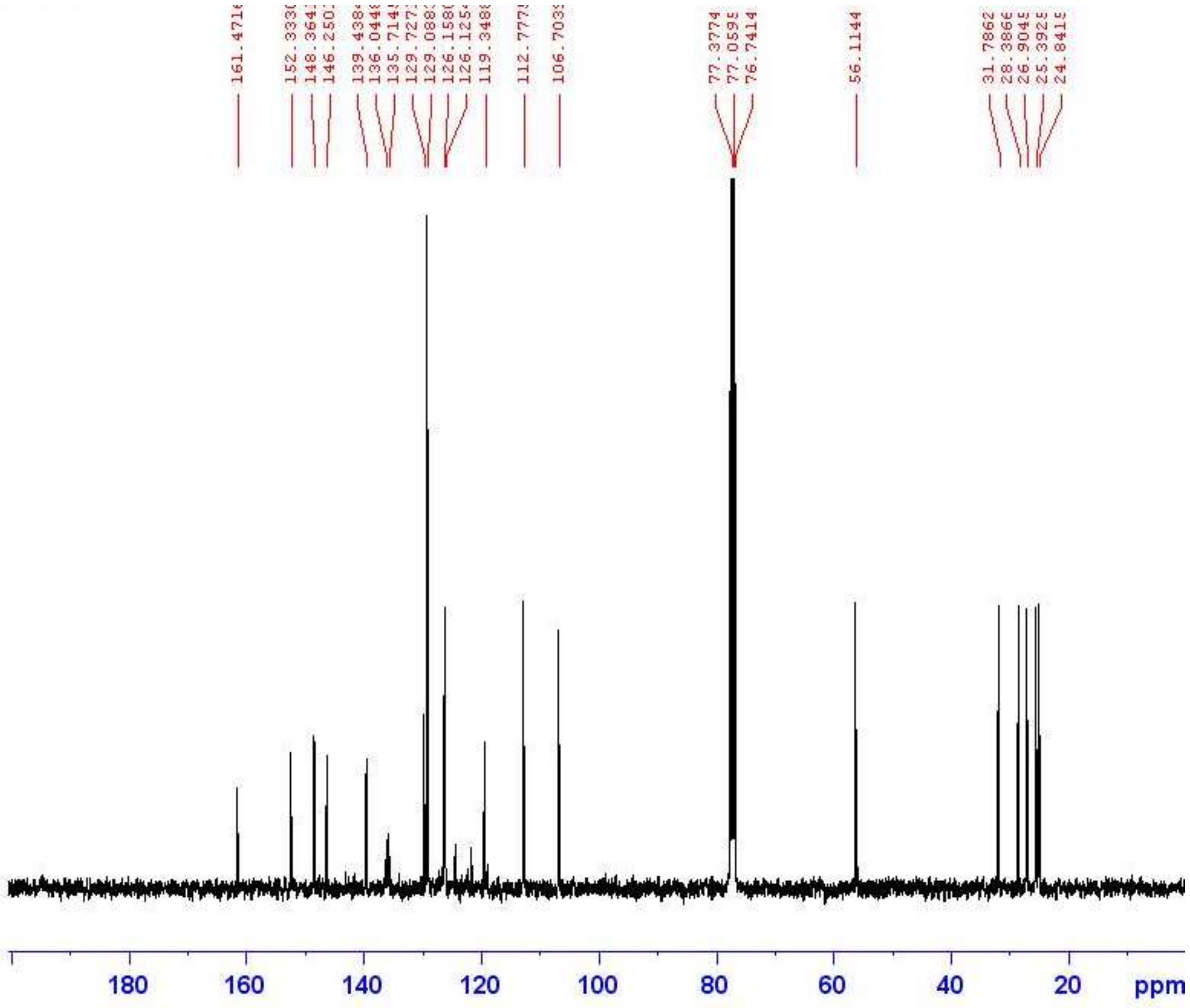

161.471
152.333
148.364
146.250
139.438
136.044
135.714
129.727
129.088
126.158
126.125
119.348
112.777
106.703
77.3774
77.0595
76.7414
56.1144
31.7862
28.3866
26.9045
25.392
24.841
180
160
140
120
100
80
60
40
20
ppm

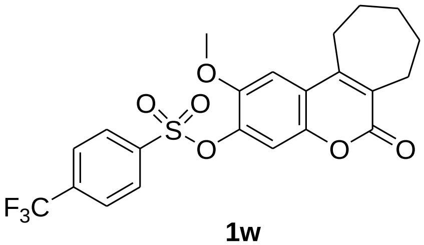

1w

Chemical Formula: $C_{22}H_{19}F_3O_6S$
Exact Mass: 468.09
Molecular Weight: 468.44

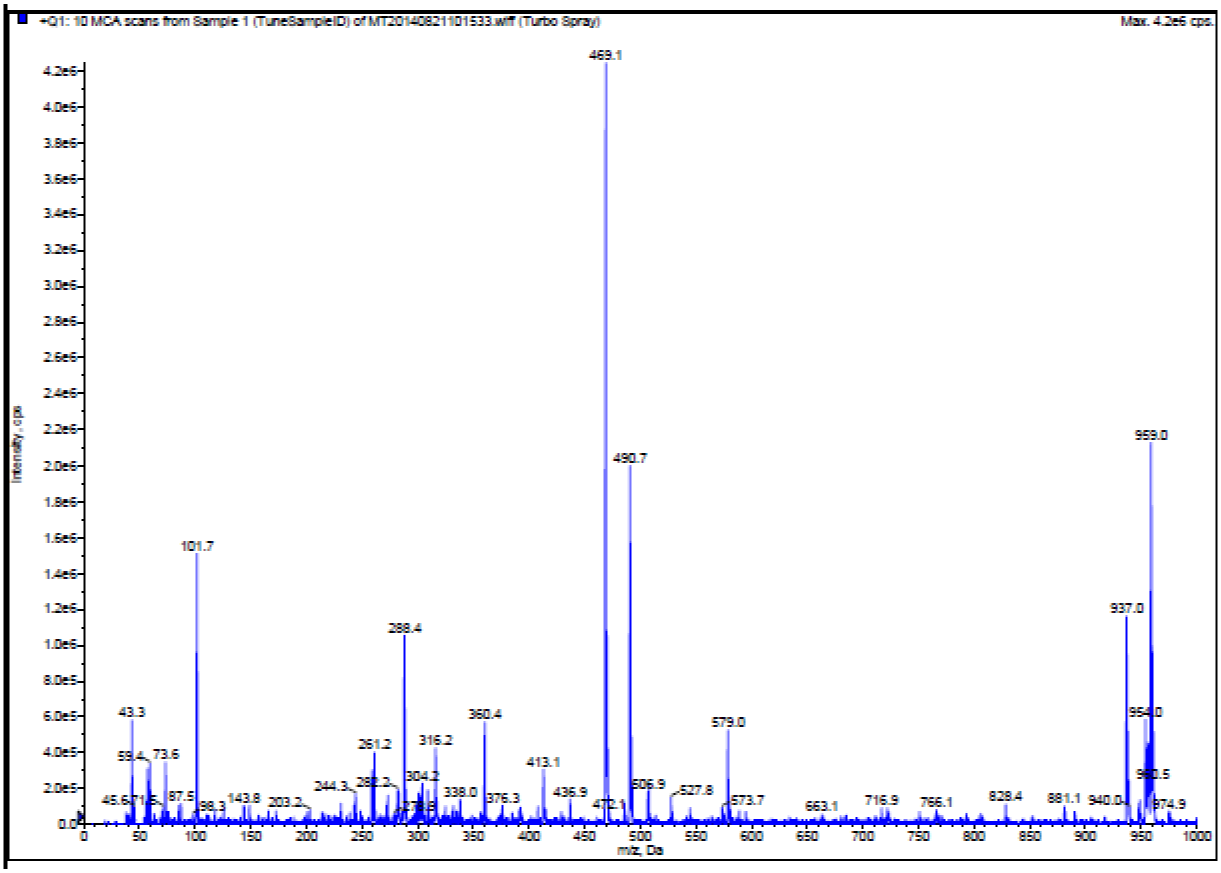

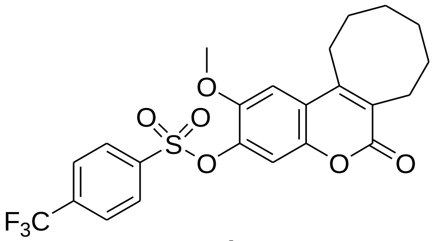

1x

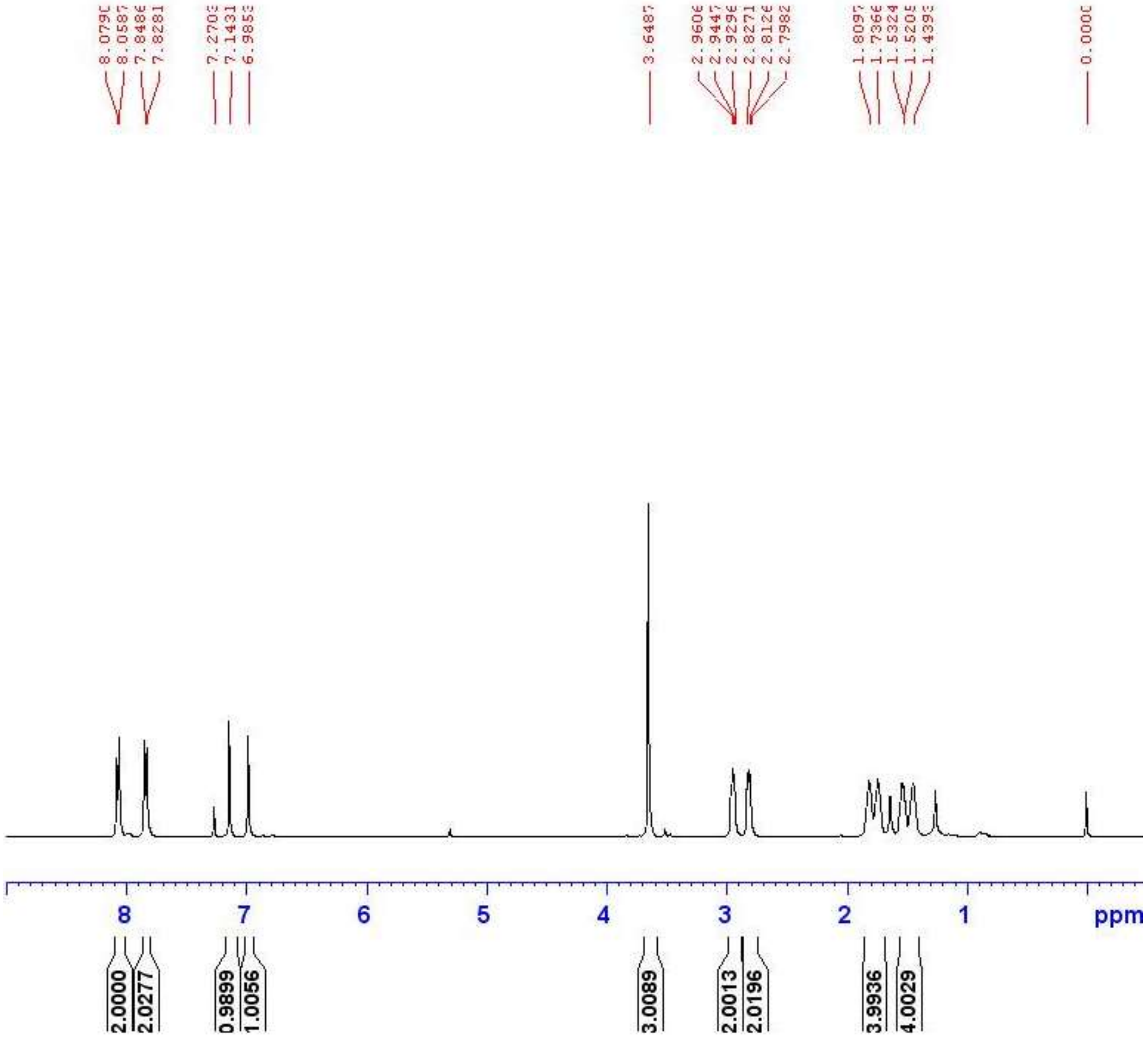
8.0790
8.0587
7.8486
7.8281
7.2703
7.1431
6.9853
3.6487
2.9606
2.9447
2.9296
2.8271
2.8126
2.7982
1.8097
1.7366
1.5324
1.5205
1.4393
0.0000
8
7
6
5
4
3
2
1
ppm
2.0000
2.0277
0.9899
1.0056
3.0089
2.0013
2.0196
3.9936
4.0029

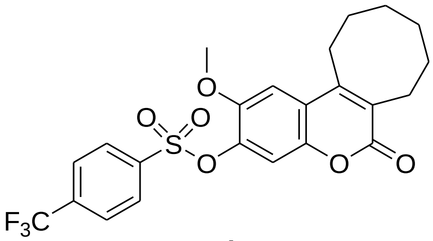
O
O
S
O
O
O
O
F3C

1x

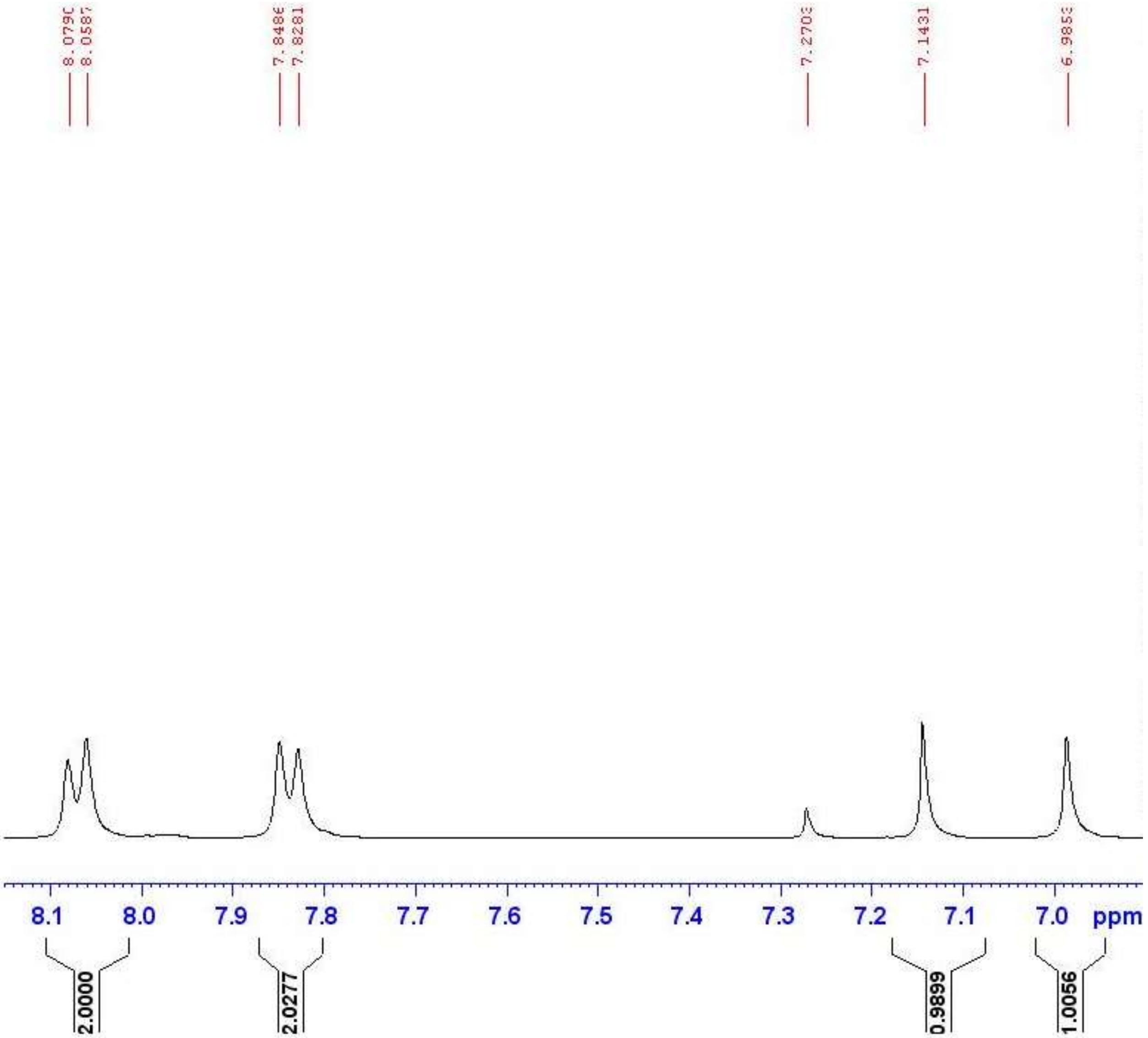
8.0790
8.0587
7.8486
7.8281
7.2703
7.1431
6.9853
8.1
8.0
7.9
7.8
7.7
7.6
7.5
7.4
7.3
7.2
7.1
7.0
ppm
2.0000
2.0277
0.9899
1.0056

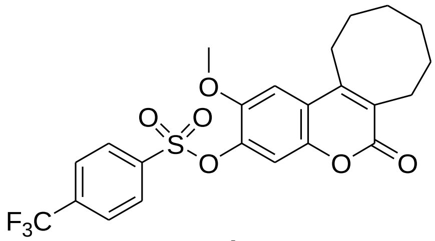
O
O
O
S
O
O
O
F3C

1x

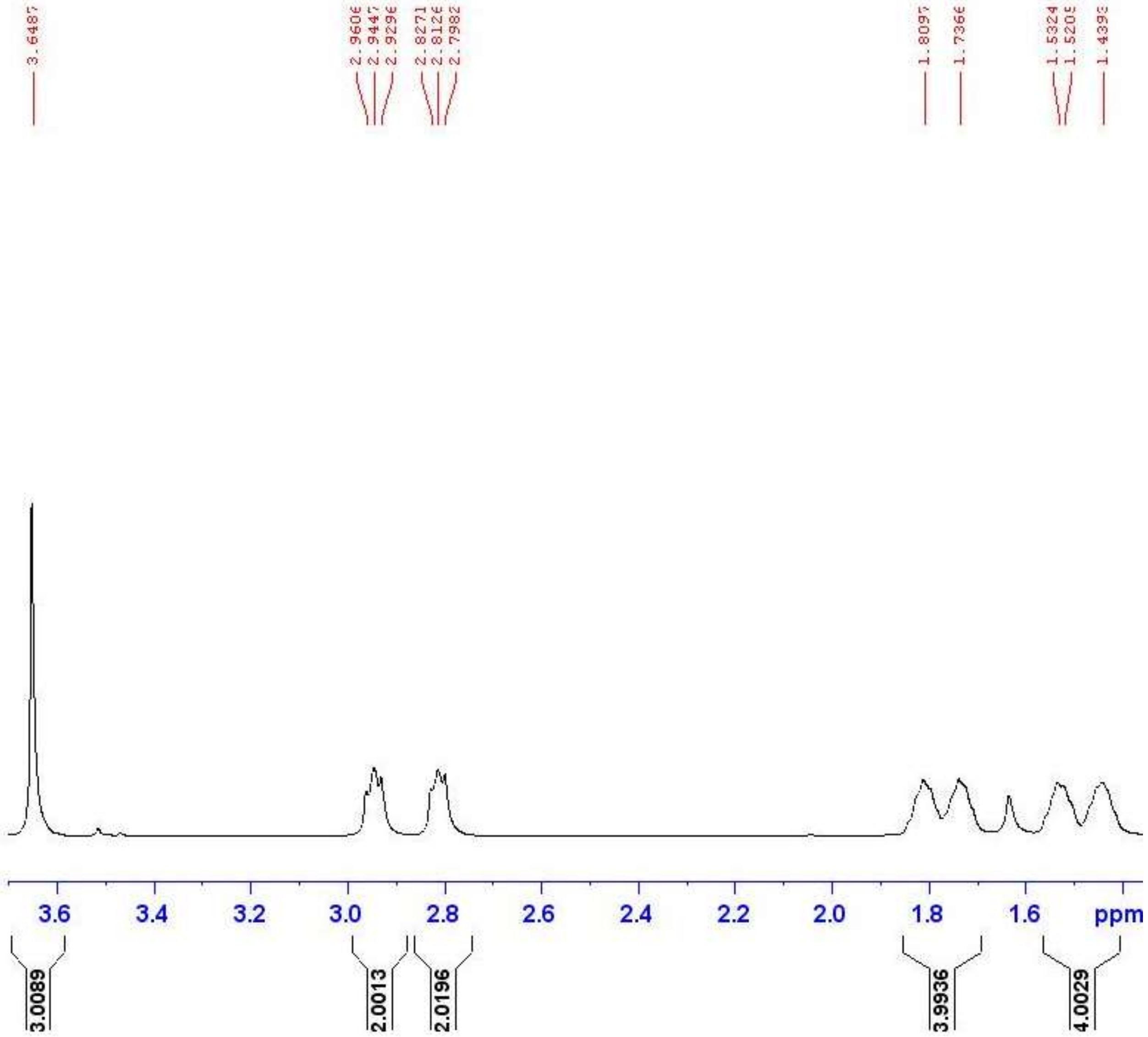
3.6487
2.9606
2.9447
2.9296
2.8271
2.8126
2.7982
1.8097
1.7366
1.5324
1.5209
1.4393
3.6
3.4
3.2
3.0
2.8
2.6
2.4
2.2
2.0
1.8
1.6
ppm
3.0089
2.0013
2.0196
3.9936
4.0029

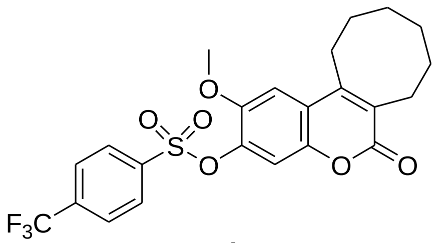

1x

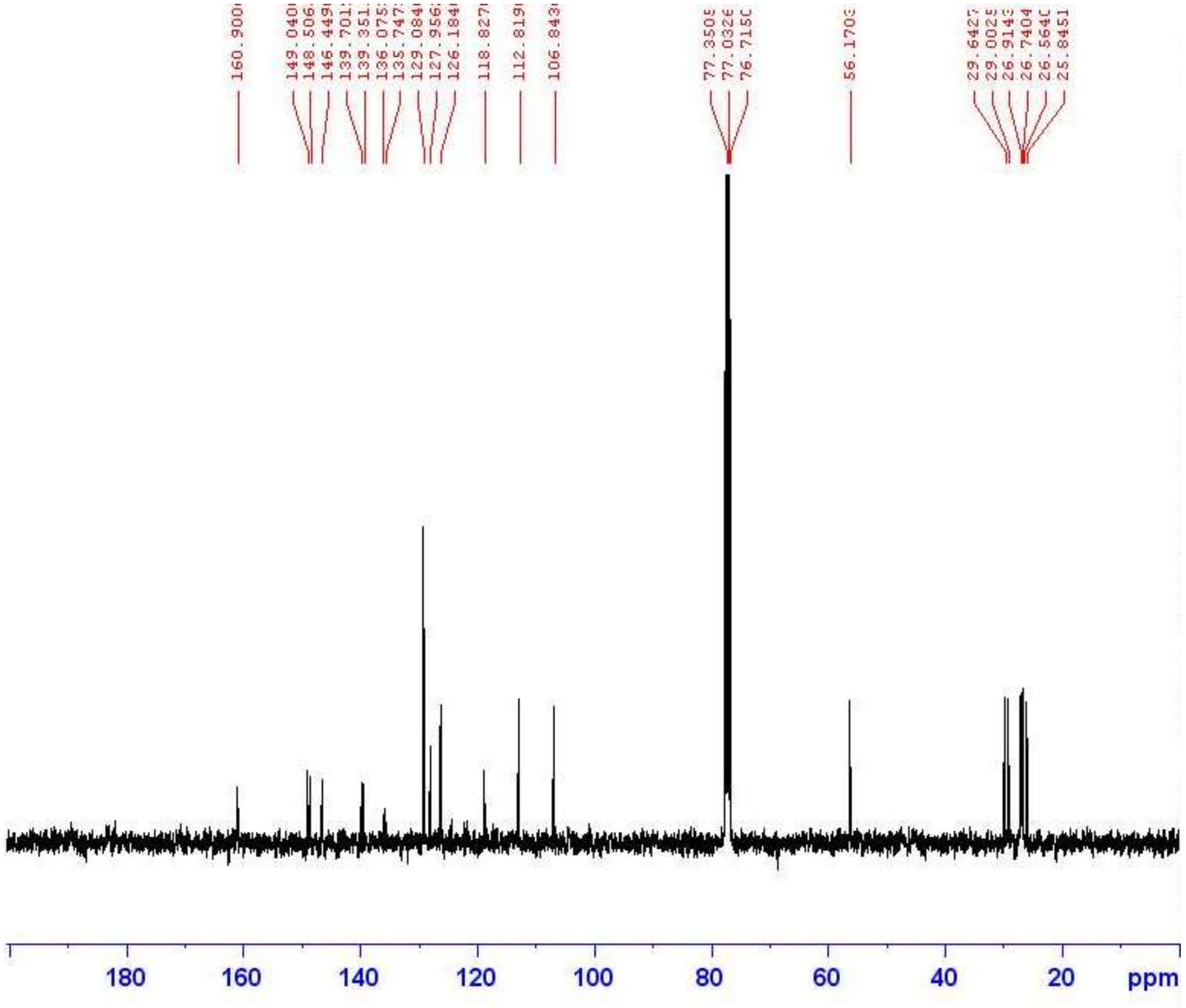

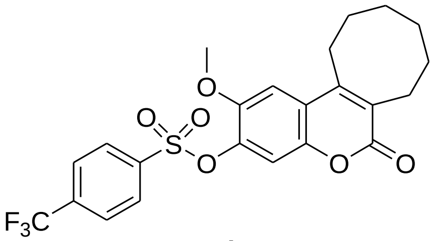

1x

Chemical Formula: $C_{23}H_{21}F_3O_6S$
Exact Mass: 482.10
Molecular Weight: 482.47

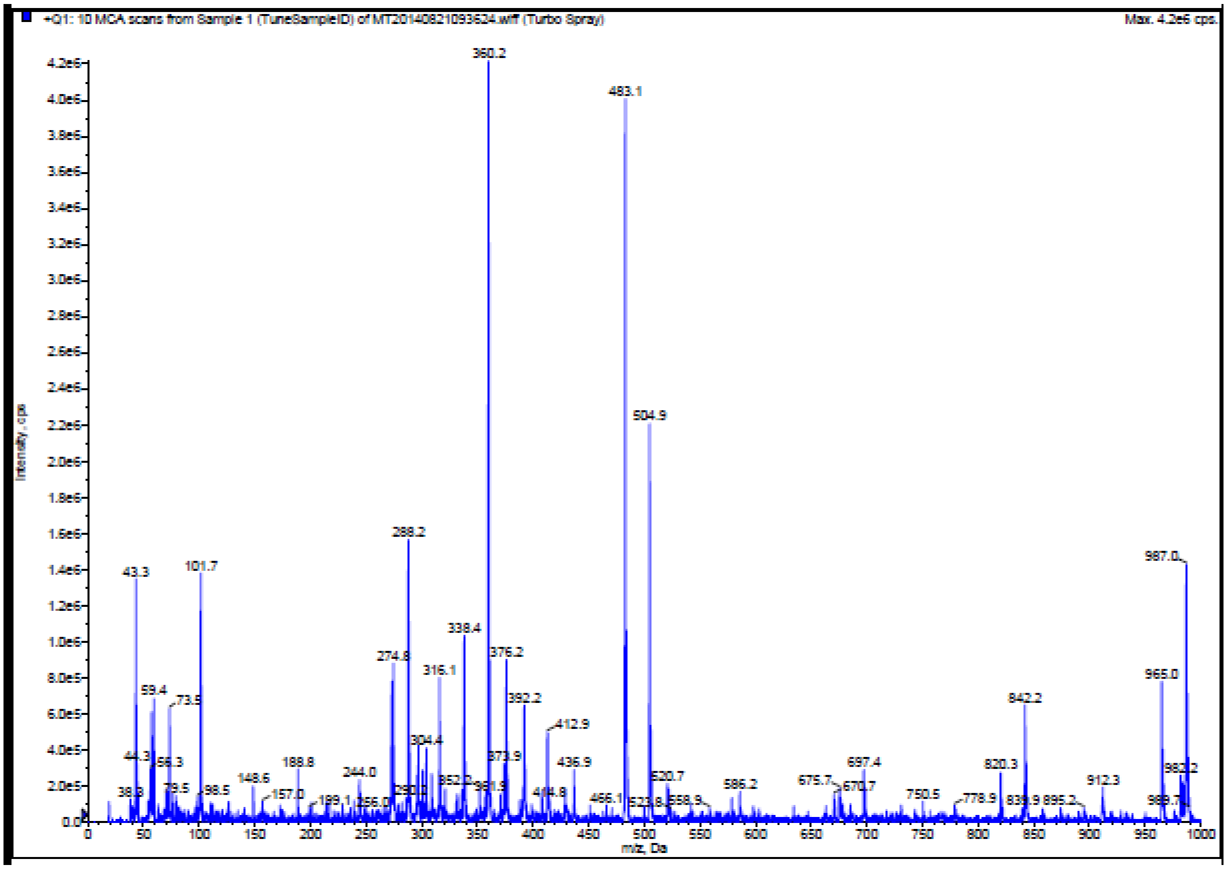

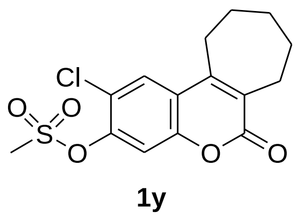
Cl
O
S
O
O
O
O
O

1y

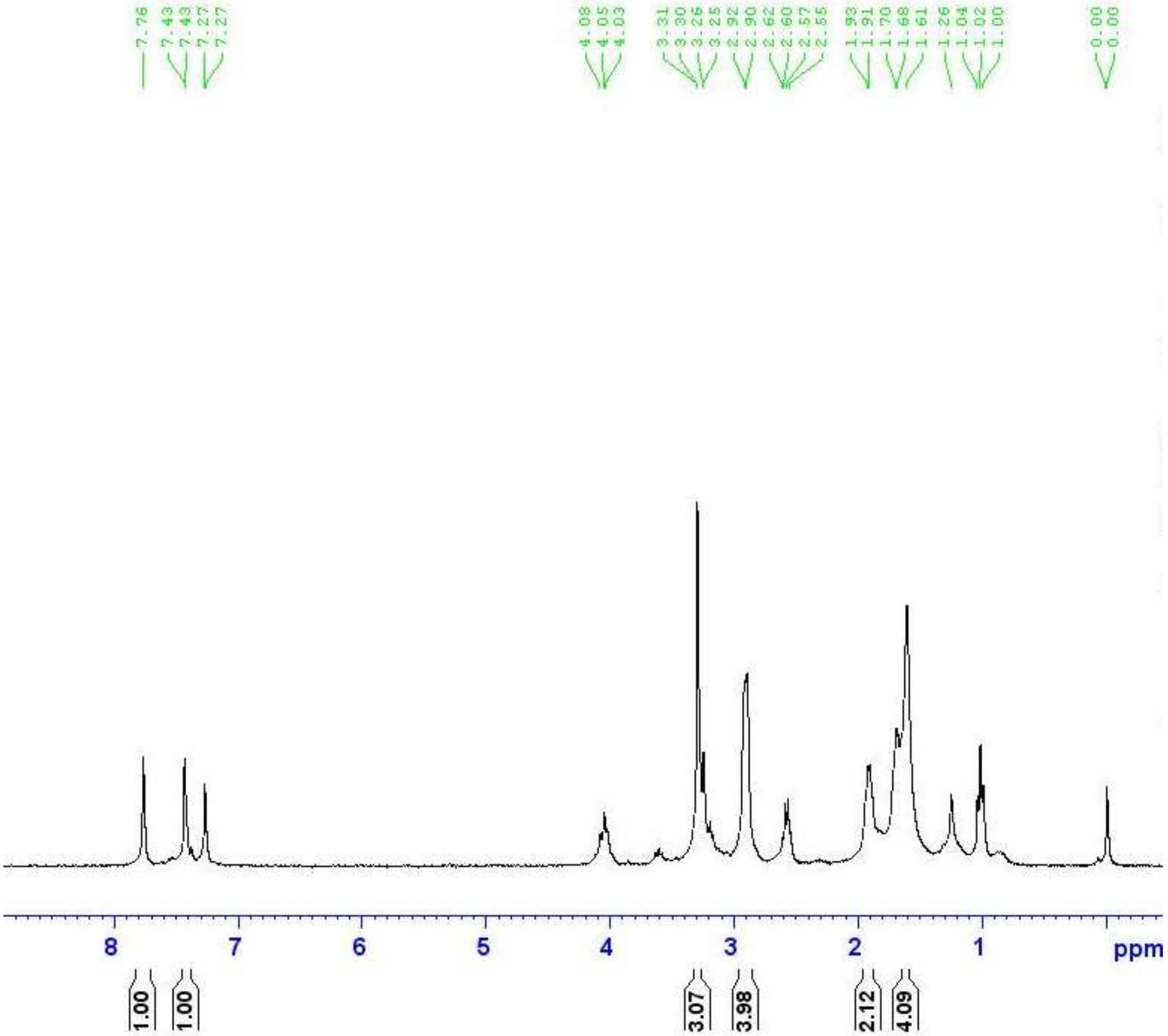
7.76
7.43
7.43
7.27
7.27
4.08
4.05
4.03
3.31
3.30
3.26
3.25
2.92
2.90
2.62
2.60
2.57
2.55
1.93
1.91
1.70
1.68
1.61
1.26
1.04
1.02
1.00
0.00
0.00
8
7
6
5
4
3
2
1
ppm
1.00
1.00
3.07
3.98
2.12
4.09

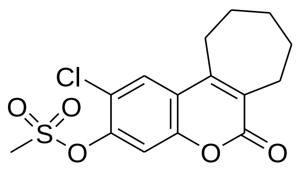

1y

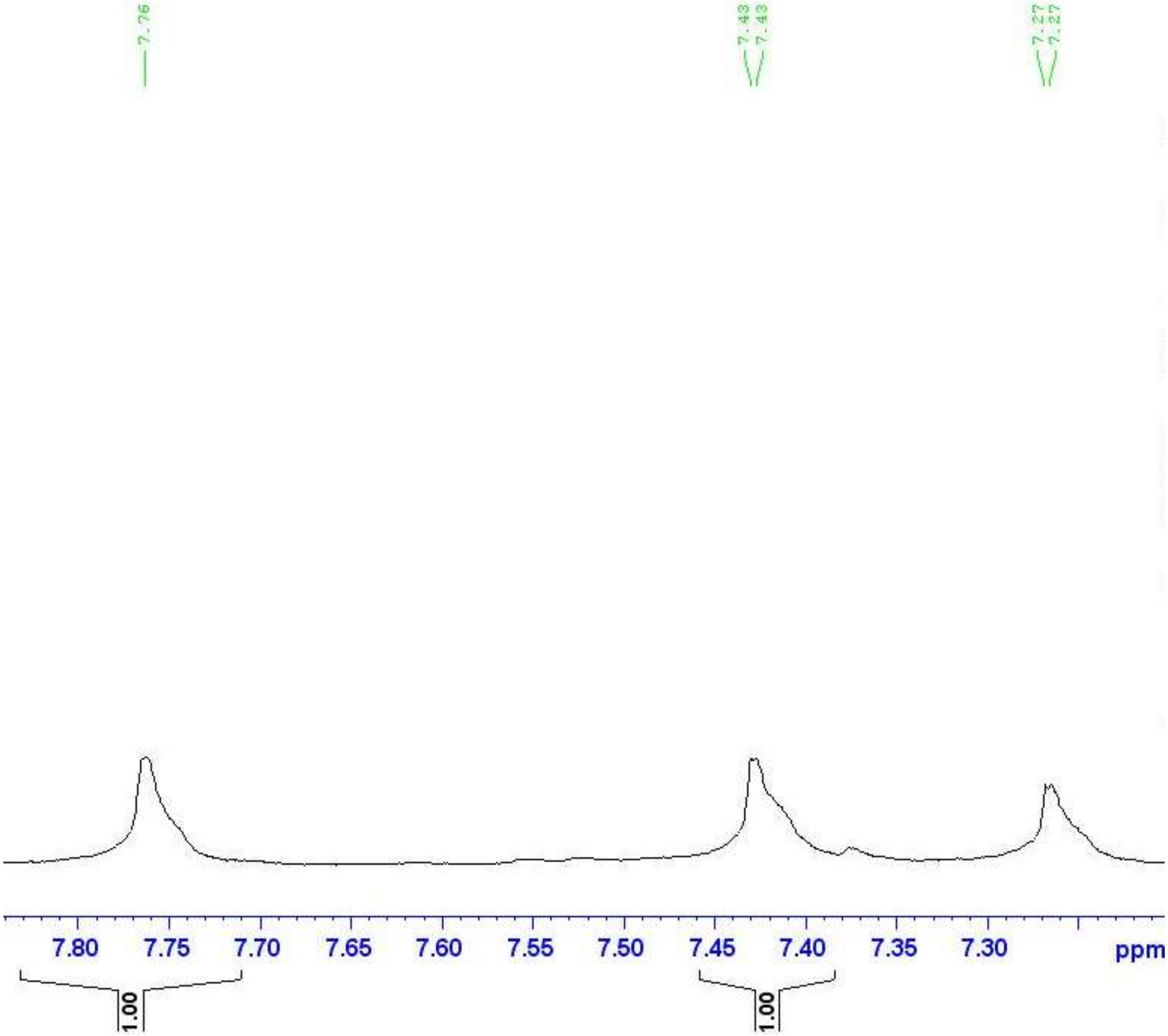
7.76
7.43
7.43
7.27
7.27
7.80
7.75
7.70
7.65
7.60
7.55
7.50
7.45
7.40
7.35
7.30
ppm
1.00
1.00

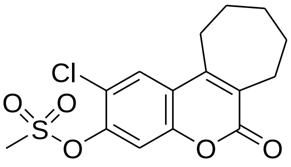
Cl
O
O
S
O
O
O

1y

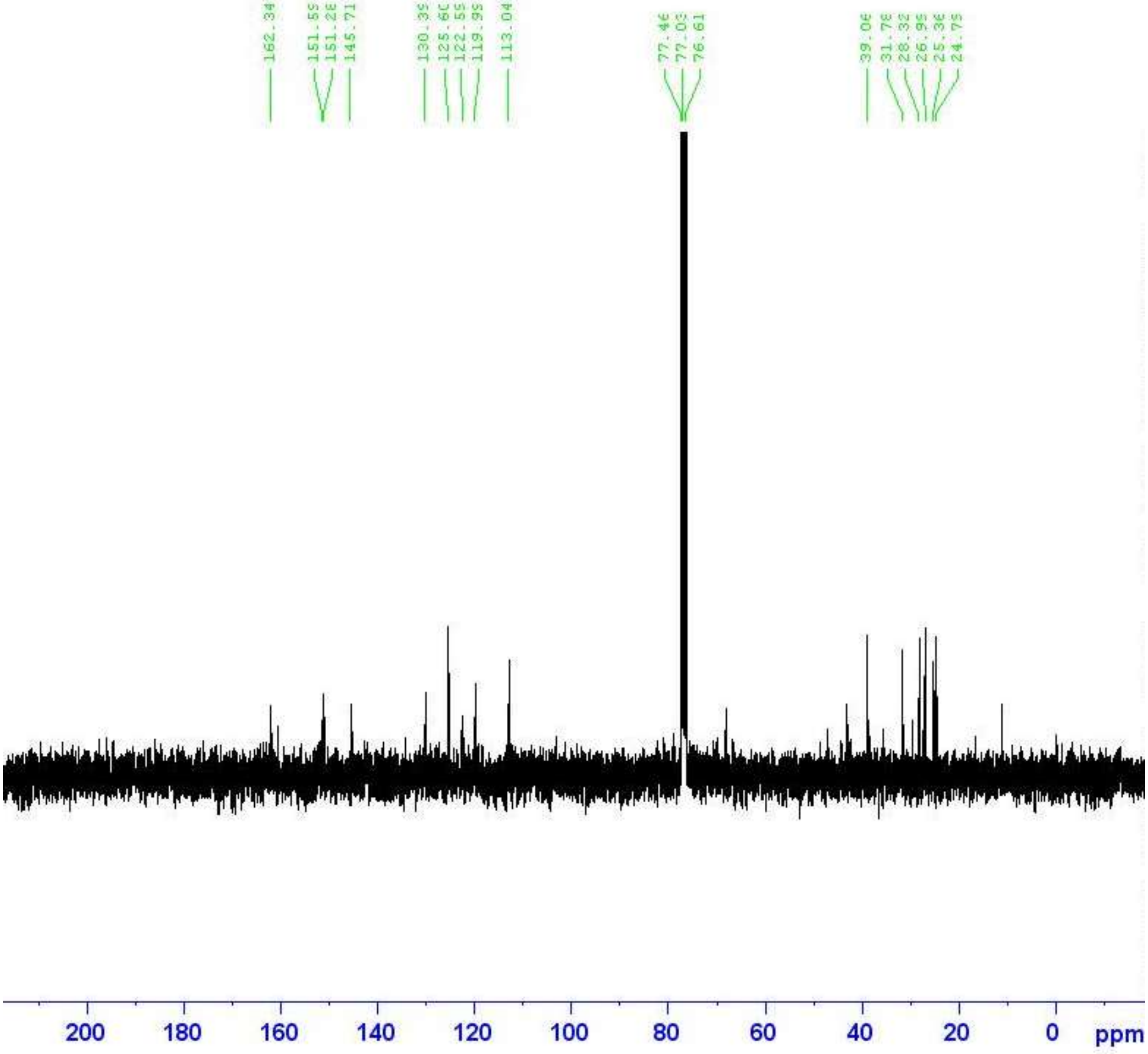
162.34
151.59
151.28
145.71
130.39
125.60
122.55
119.99
113.04
77.46
77.03
76.61
39.06
31.78
28.32
26.99
25.36
24.75
200
180
160
140
120
100
80
60
40
20
0
ppm

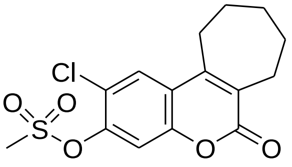

1y

Chemical Formula: $C_{15}H_{15}ClO_5S$
Exact Mass: 342.03
Molecular Weight: 342.79

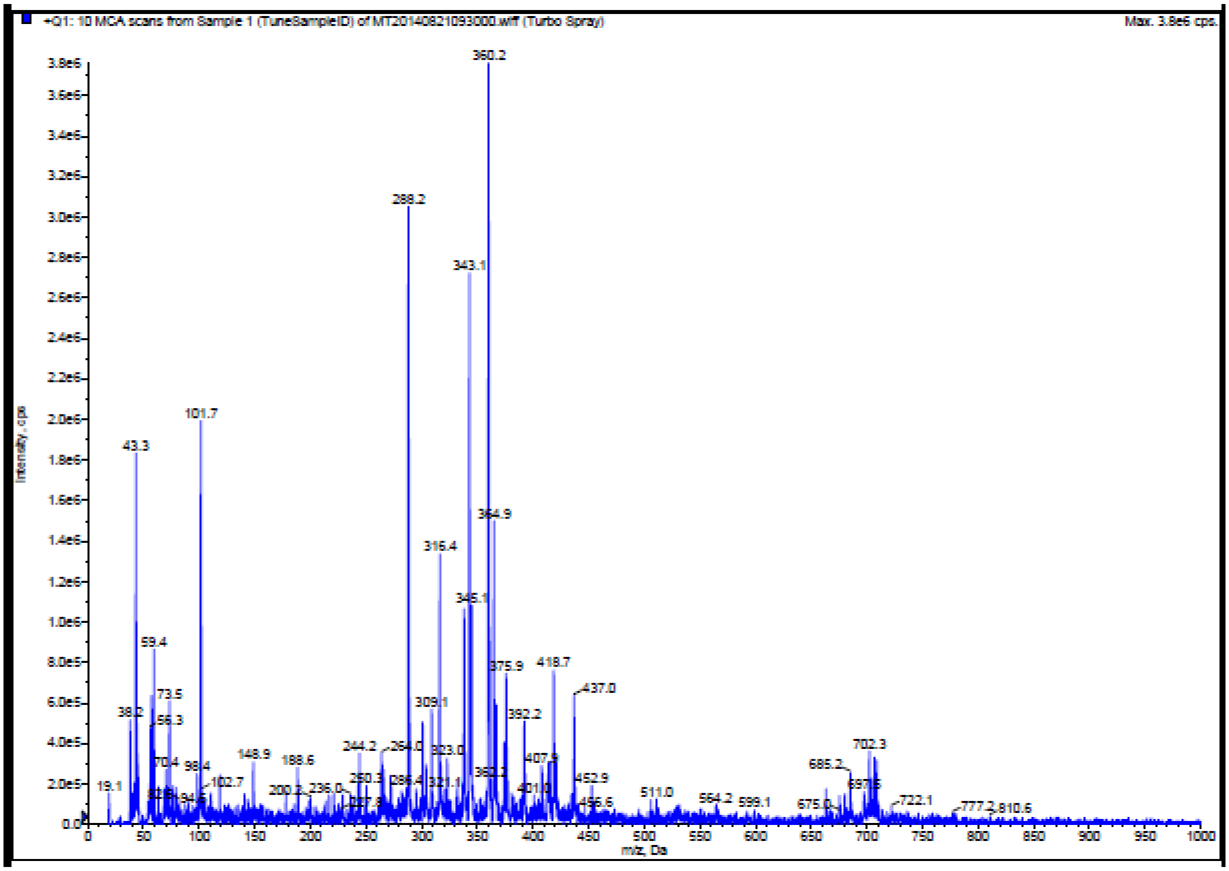

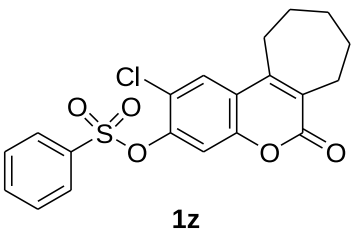
Cl
O
O
S
O
O
O

1z

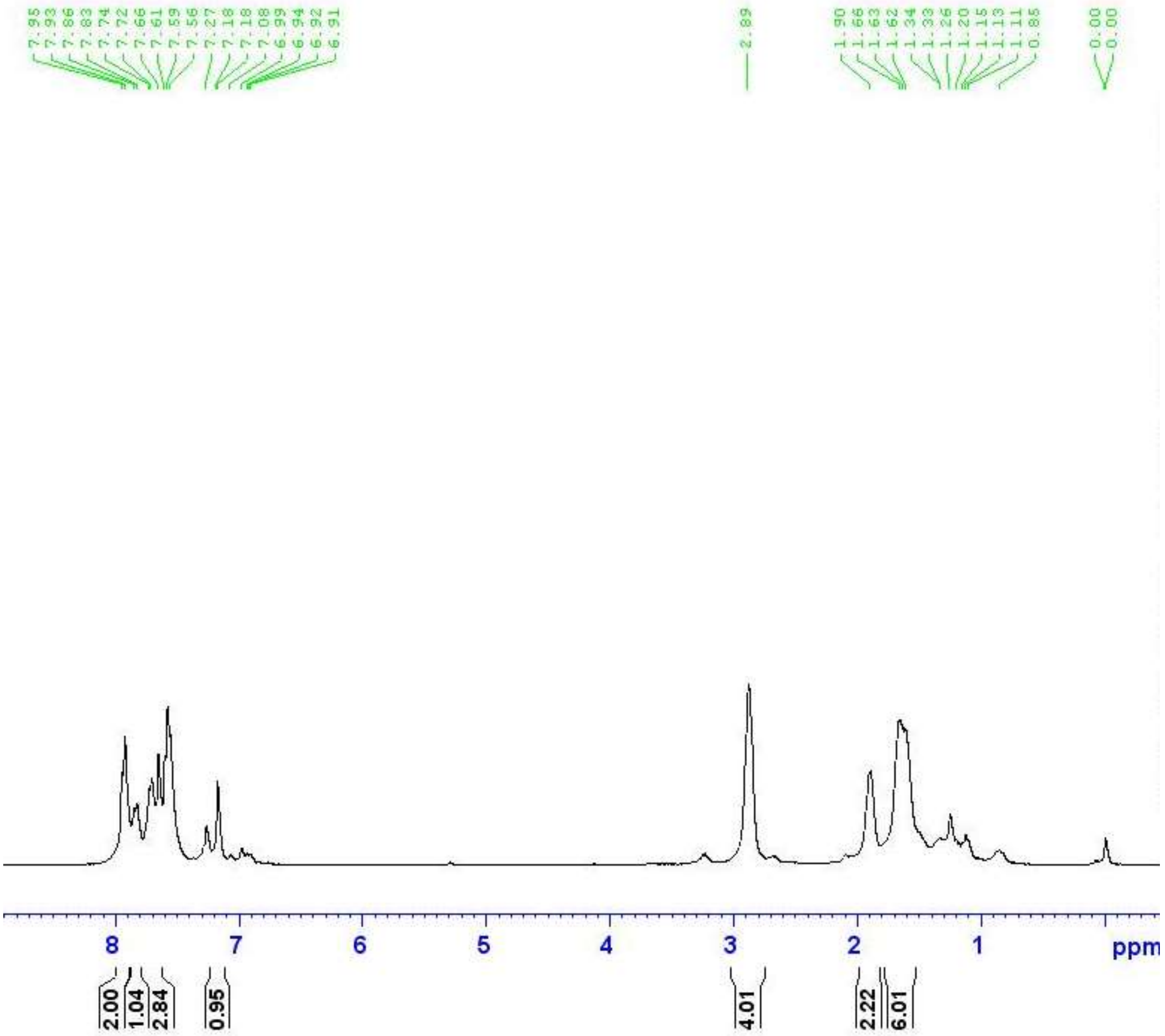
7.95 7.93 7.86 7.83 7.74 7.72 7.66 7.61 7.59 7.56 7.27 7.18 7.18 7.08 6.99 6.94 6.92 6.91
2.89
1.90 1.66 1.63 1.62 1.34 1.33 1.26 1.20 1.15 1.13 1.11 0.85
0.00 0.00
8 7 6 5 4 3 2 1 ppm
2.00 1.04 2.84 0.95 4.01 2.22 6.01

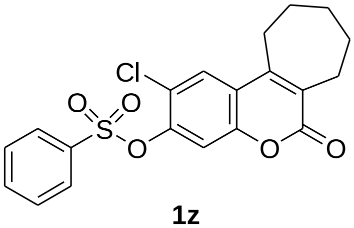
Cl
O
S
O
O
O
O

1z

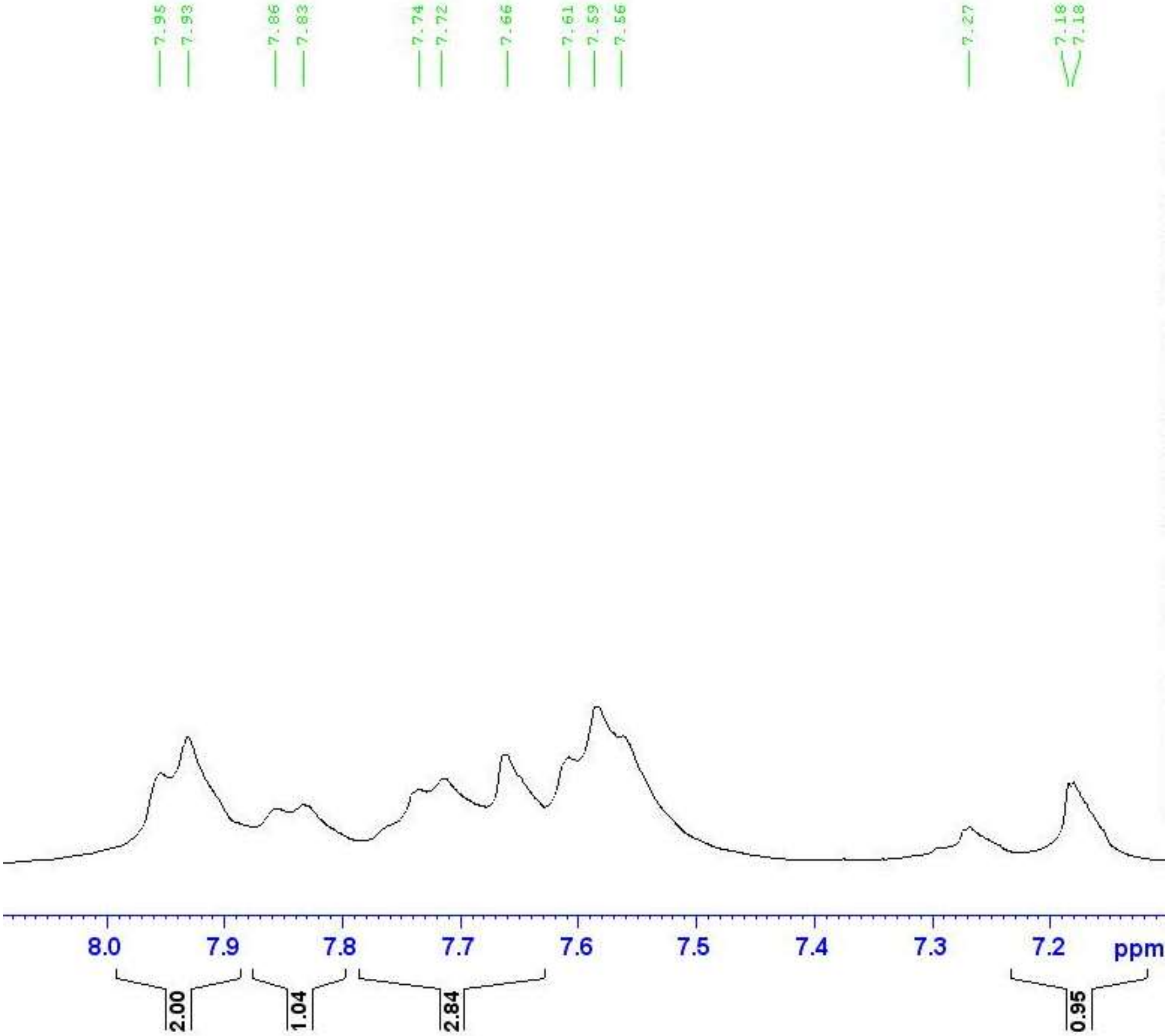
7.95
7.93
7.86
7.83
7.74
7.72
7.66
7.61
7.59
7.56
7.27
7.18
7.18
8.0
7.9
7.8
7.7
7.6
7.5
7.4
7.3
7.2
ppm
2.00
1.04
2.84
0.95

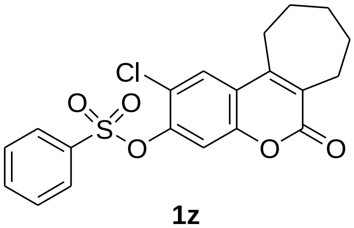
Cl
O
O
S
O
O
O

1z

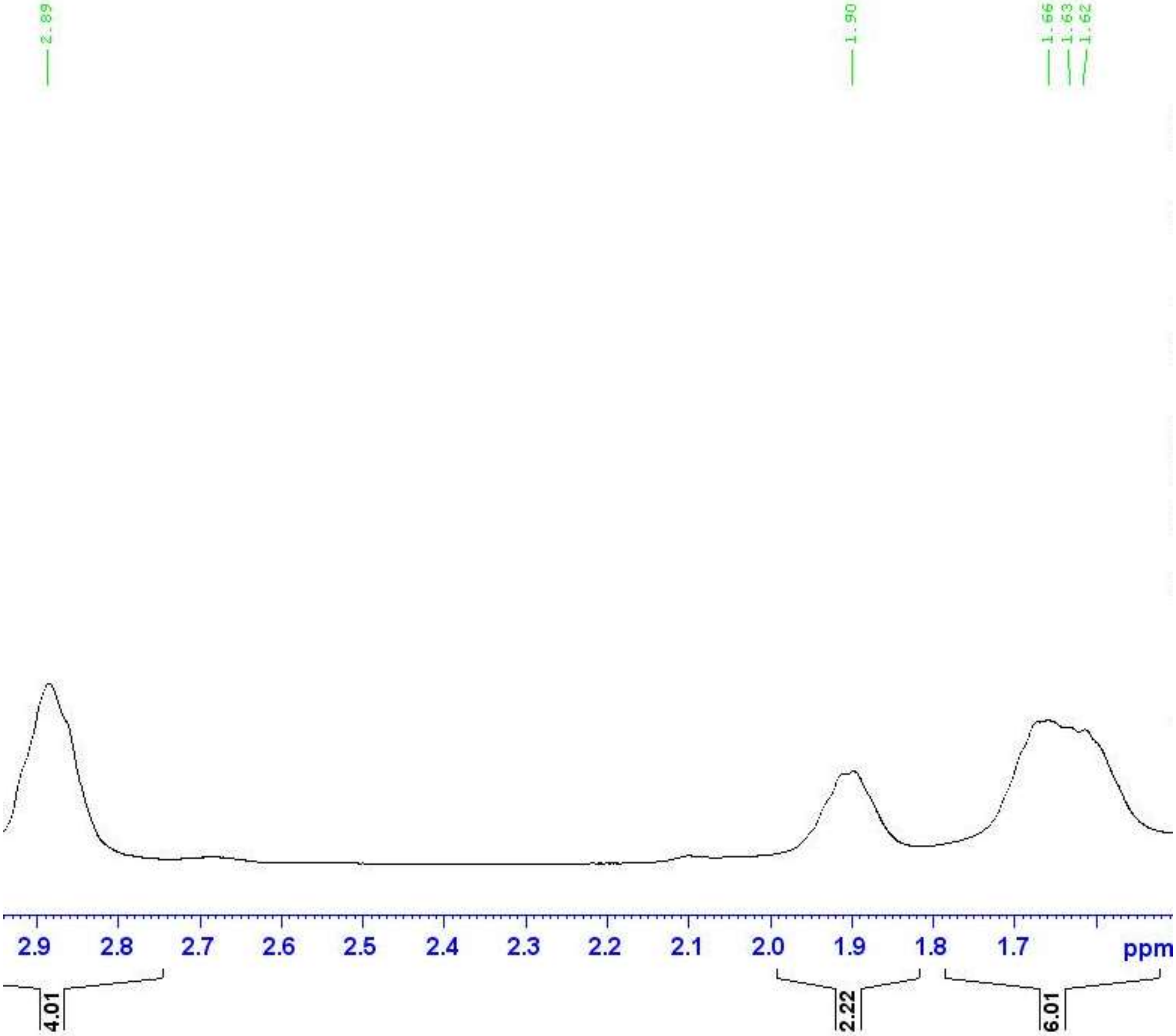
2.89
1.90
1.66
1.63
1.62
2.9 2.8 2.7 2.6 2.5 2.4 2.3 2.2 2.1 2.0 1.9 1.8 1.7 ppm
4.01
2.22
6.01

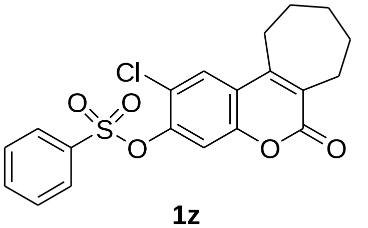

1z

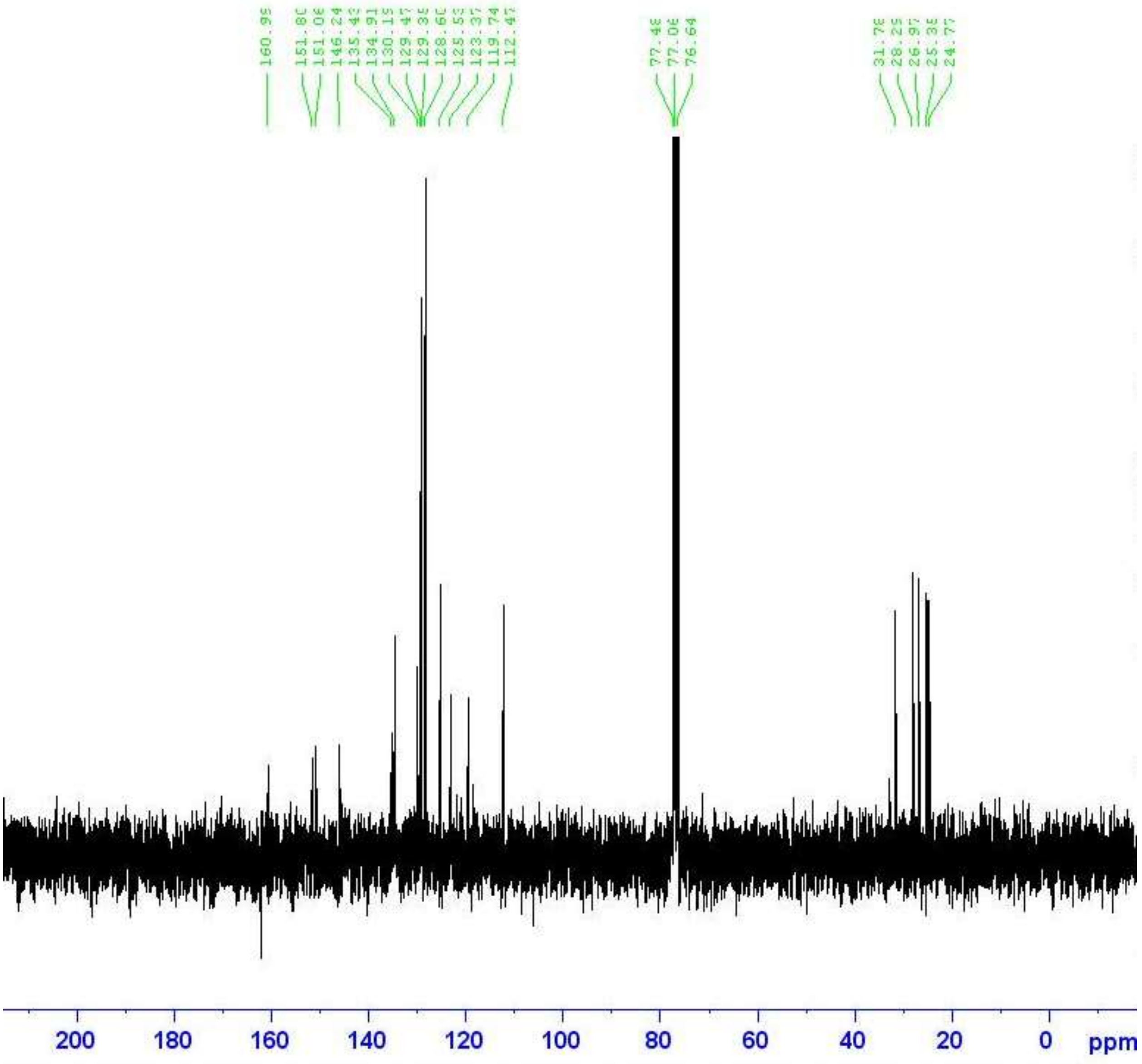
160.99
151.80
151.06
146.24
135.43
134.91
130.19
129.47
129.39
128.60
125.53
123.37
119.74
112.47
77.48
77.06
76.64
31.78
28.29
26.97
25.38
24.77
200
180
160
140
120
100
80
60
40
20
0
ppm

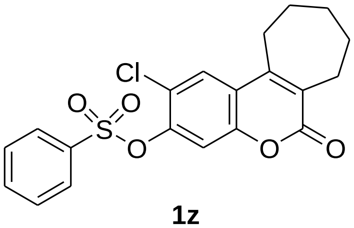

1z

Chemical Formula: $C_{20}H_{17}ClO_5S$
Exact Mass: 404.05
Molecular Weight: 404.86

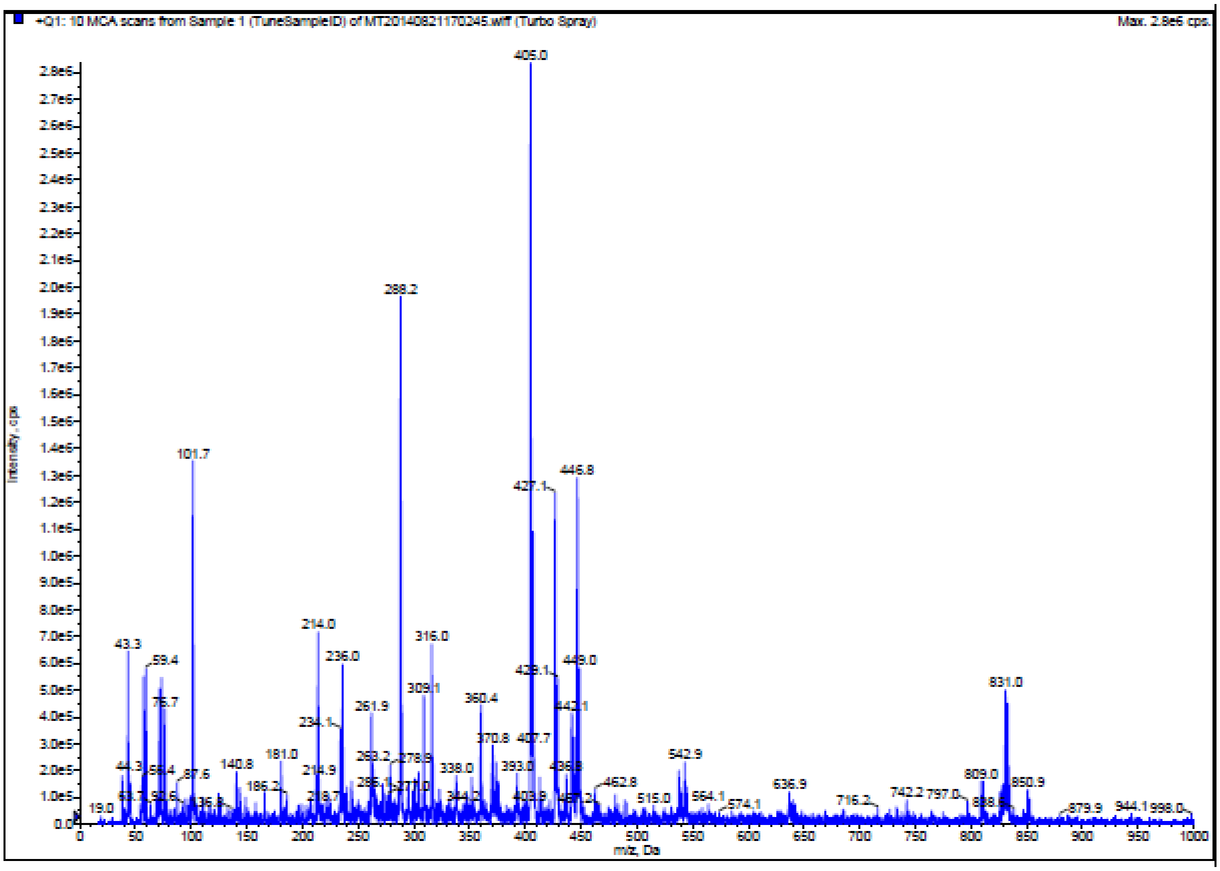

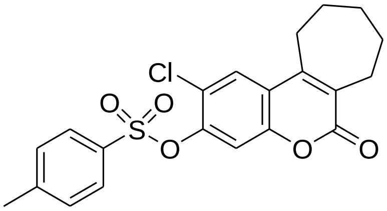
Cl
O
S
O
O
O
O

1za

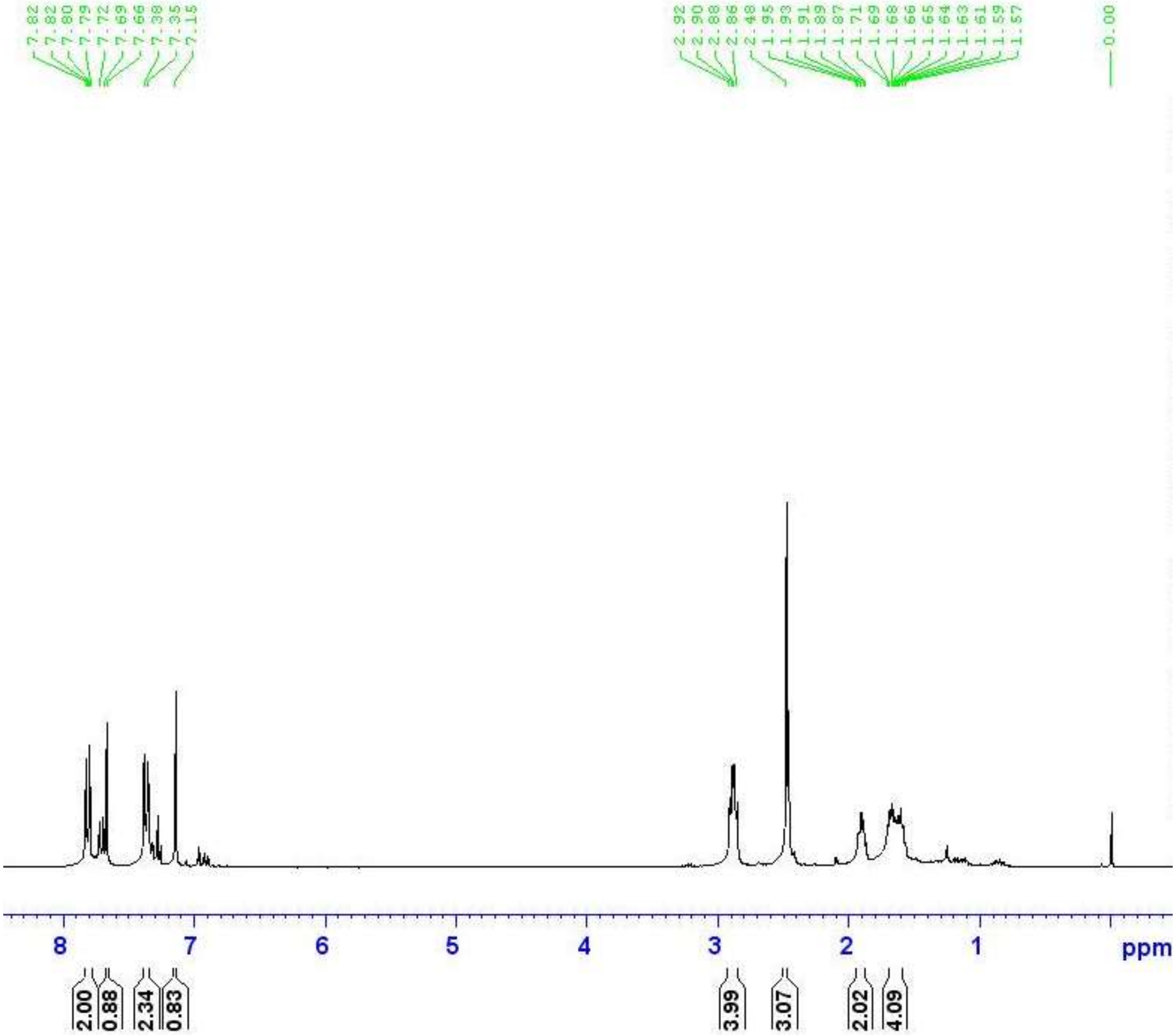
7.82
7.82
7.80
7.79
7.72
7.69
7.66
7.38
7.35
7.15
2.92
2.90
2.88
2.86
2.48
1.95
1.93
1.91
1.89
1.87
1.71
1.69
1.68
1.66
1.65
1.64
1.63
1.61
1.59
1.57
0.00
8
7
6
5
4
3
2
1
ppm
2.00
0.88
2.34
0.83
3.99
3.07
2.02
4.09

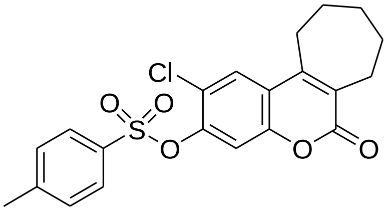

1za

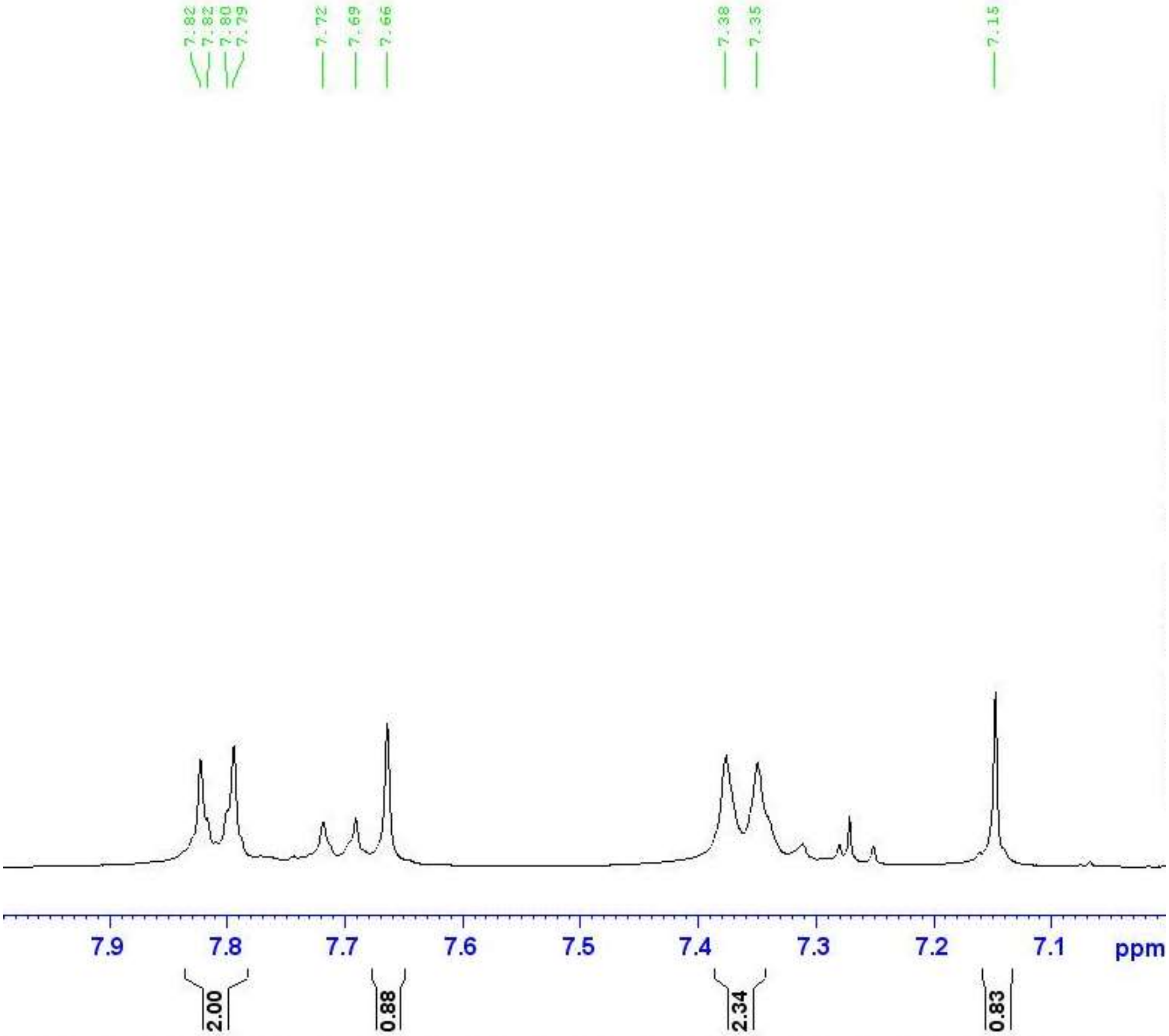
7.82
7.82
7.80
7.79
7.72
7.69
7.66
7.38
7.35
7.15
7.9
7.8
7.7
7.6
7.5
7.4
7.3
7.2
7.1
ppm
2.00
0.88
2.34
0.83

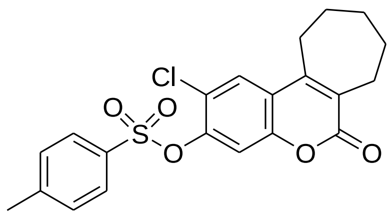

1za

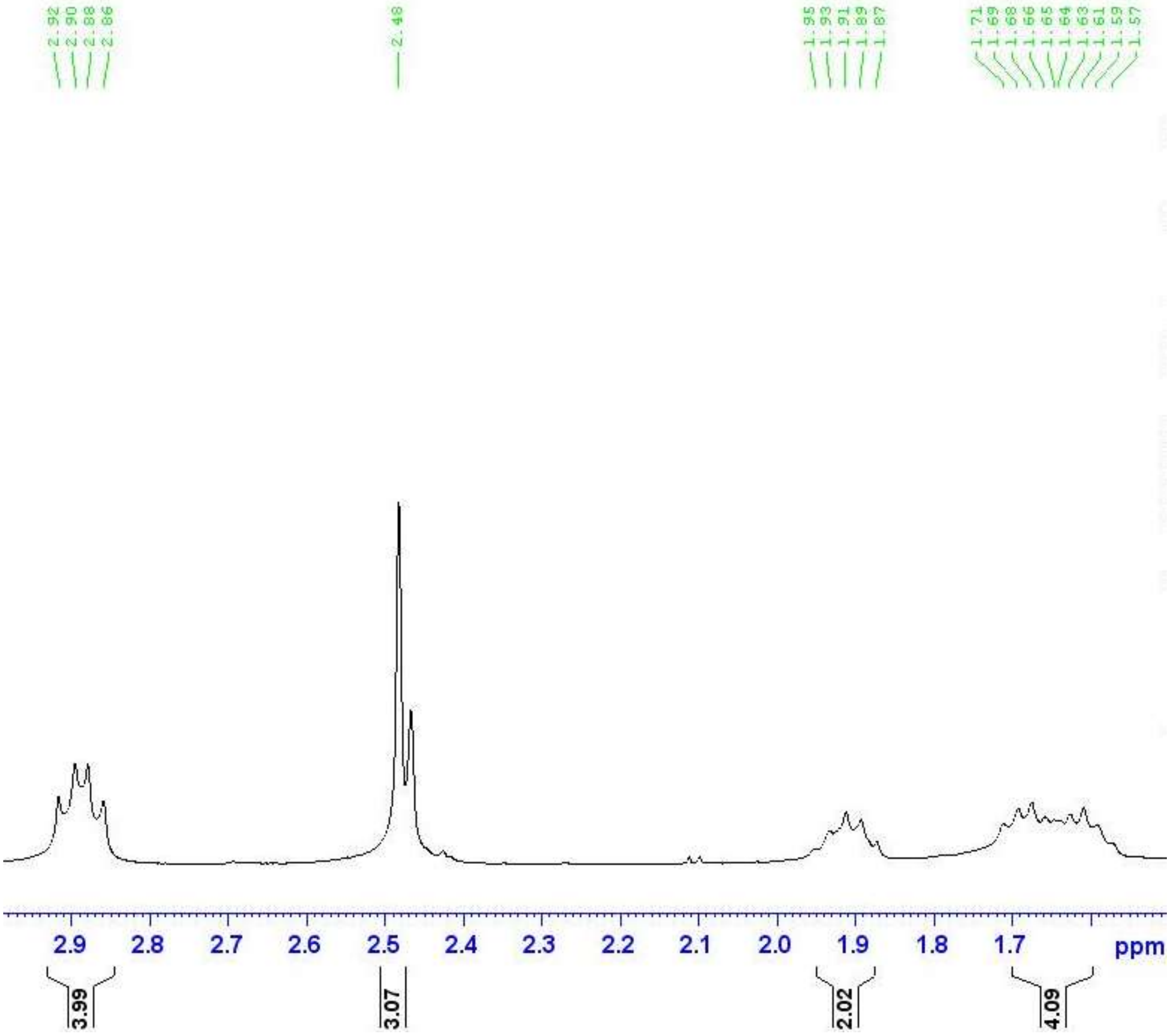

2.92
2.90
2.88
2.86
2.48
1.95
1.93
1.91
1.89
1.87
1.71
1.69
1.68
1.66
1.65
1.64
1.63
1.61
1.59
1.57
2.9
2.8
2.7
2.6
2.5
2.4
2.3
2.2
2.1
2.0
1.9
1.8
1.7
ppm
3.99
3.07
2.02
4.09

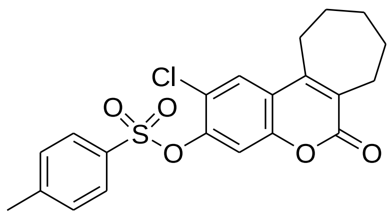
Cl
O
S
O
O
O
O

1za

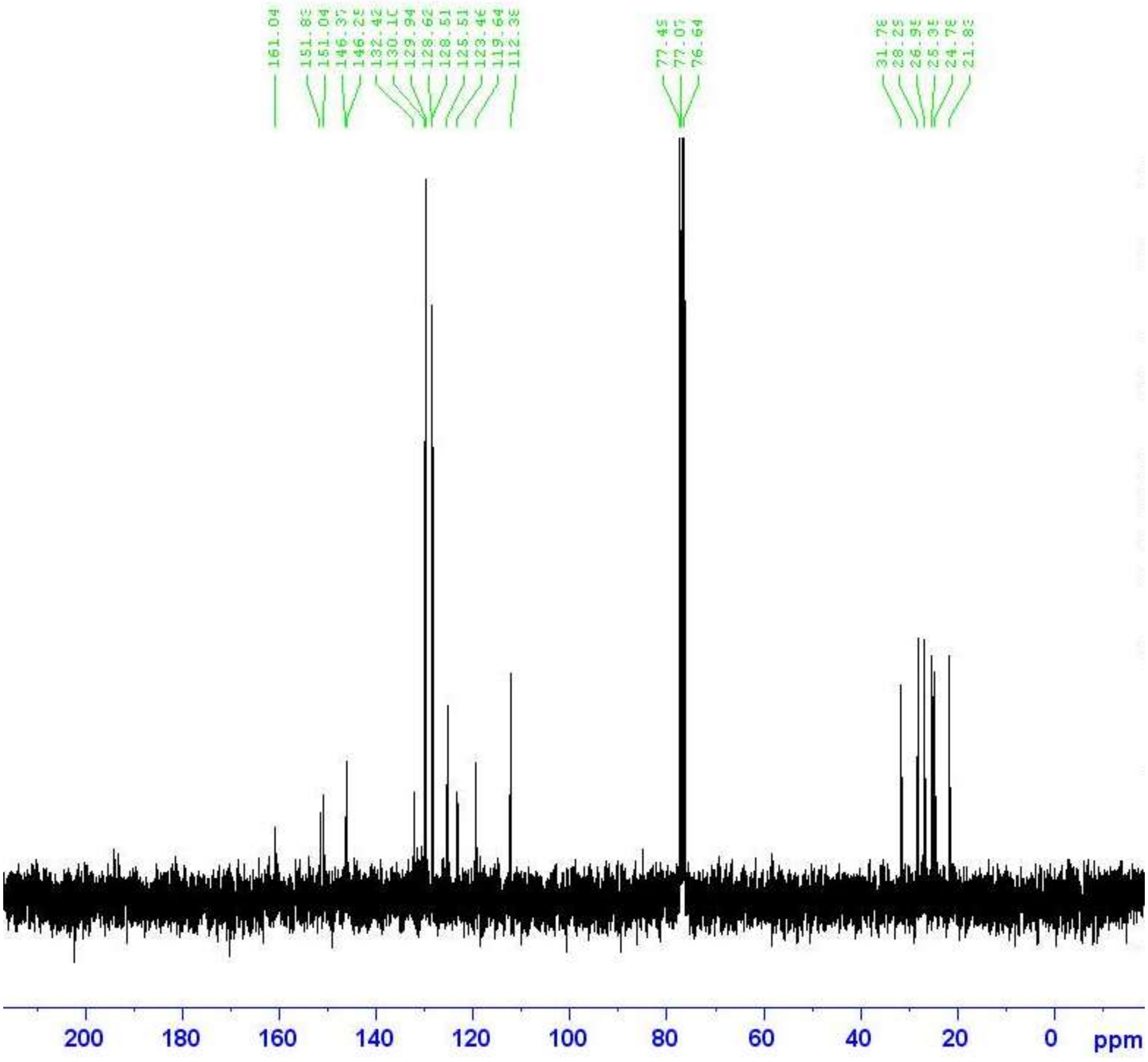
161.04
151.83
151.04
146.37
146.28
132.42
130.10
129.94
128.62
128.51
125.51
123.46
119.64
112.38
77.45
77.07
76.64
31.78
28.29
26.95
25.39
24.78
21.83
200
180
160
140
120
100
80
60
40
20
0
ppm

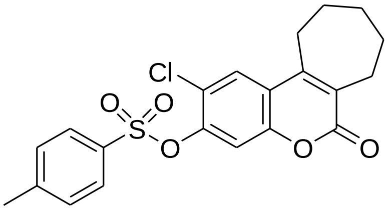

1za

Chemical Formula: $C_{21}H_{19}ClO_5S$
Exact Mass: 418.06
Molecular Weight: 418.89

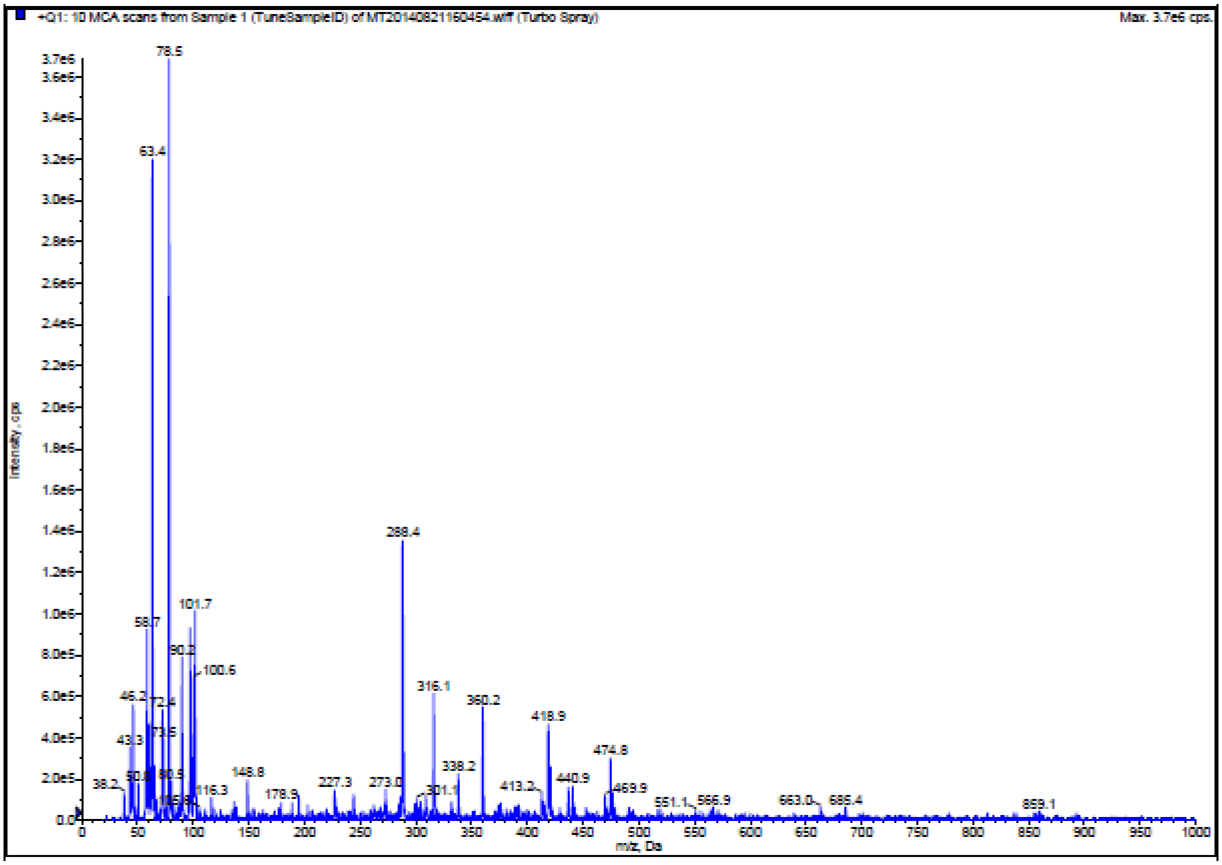

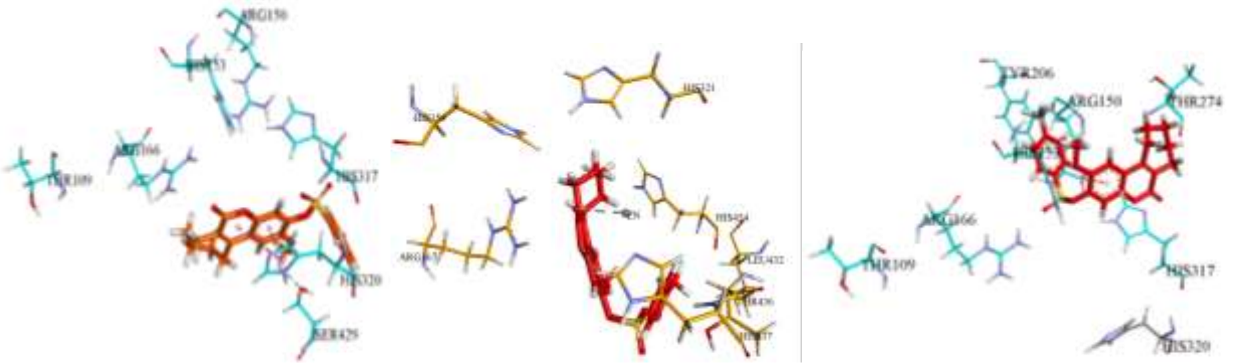

(**1q**; *h*-IAP) (**1w**; *h*-TNAP) (**1w**; *h*-IAP)

Docking poses of the inactive derivatives **1q** and **1w**
